# Gene Expression of Protein-Coding and Non-Coding RNAs Related to Polyembryogenesis in the Parasitic Wasp, *Copidosoma floridanum*

**DOI:** 10.1371/journal.pone.0114372

**Published:** 2014-12-03

**Authors:** Hiroki Inoue, Jin Yoshimura, Kikuo Iwabuchi

**Affiliations:** 1 Faculty of Agriculture, Tokyo University of Agriculture and Technology, Fuchu, Tokyo, Japan; 2 Graduate School of Science and Technology, and Department of Mathematical and Systems Engineering, Shizuoka University, Hamamatsu, Shizuoka, Japan; 3 Department of Environmental and Forest Biology, State University of New York College of Environmental Science and Forestry, Syracuse, New York, United States of America; 4 Marine Biosystems Research Center, Chiba University, Kamogawa, Chiba, Japan; NIAID, United States of America

## Abstract

Polyembryony is a unique form of development in which many embryos are clonally produced from a single egg. Polyembryony is known to occur in many animals, but the underlying genetic mechanism responsible is unknown. In a parasitic wasp, *Copidosoma floridanum*, polyembryogenesis is initiated during the formation and division of the morula. In the present study, cDNA libraries were constructed from embryos at the cleavage and subsequent primary morula stages, times when polyembryogenesis is likely to be controlled genetically. Of 182 and 263 cDNA clones isolated from these embryos, 38% and 70%, respectively, were very similar to protein-coding genes obtained from BLAST analysis and 55 and 65 clones, respectively, were stage-specific. In our libraries we also detected a high frequency of long non-coding RNA. Some of these showed stage-specific expression patterns in reverse transcription quantitative polymerase chain reaction (RT-qPCR) analysis. The stage-specificity of expression implies that these protein-coding and non-coding genes are related to polyembryogenesis in *C. floridanum*. The non-coding genes are not similar to any known non-coding RNAs and so are good candidates as regulators of polyembryogenesis.

## Introduction

Polyembryony is known to occur in many animals. In insects it has been thought that polyembryony has evolved independently in four families of Hymenoptera and one genus of Strepsiptera [1]–[3]. Development of all of these polyembryonic insects is characterized by prolonged embryonic stages and the production of genetically identical progeny. However, the detailed mechanisms of polyembryony differ among these phylogenetic groups. The most studied polyembryonic insect is *Copidosoma floridanum* (Hymenoptera: Encyrtidae), which is an egg-larval parasitoid of plusiine moths. The embryogenesis of *C. floridanum* is different from that of typical insects in that the syncytial (superficial or peripheral) cleavage results in the formation of the syncytial blastoderm. In *C. floridanum* the parasitoid egg cleaves nearly holoblastically to form a morula, which is a spherical mass of embryonic cells surrounded by an extra-embryonic syncytium [4]–[7]. The morulae then continuously divide to form polyembryos inside the growing host larva, and subsequently start morphogenesis. They then produce more than 2000 parasitoid larvae per host larva [3], [8]–[10]. The division of each *C. floridanum* morula into polyembryos is initiated by invagination of the extra-embryonic syncytium into the spherical mass of embryonic cells [2], [7], [11].

Developmental analyses of *C. floridanum* at the cellular level have revealed that cleavage-stage development shows several analogies to mammalian embryogenesis, including the early separation of extraembryonic and embryonic cell lineages, formation of a morula and embryonic compaction [7]. Thus, the embryogenesis of *C. floridanum* differs from that of most other insects, both at the cleavage and primary morula stages. These unusual states during early development might have provided favourable evolutionary conditions for polyembryony in *C. floridanum* or in its ancestors. Therefore, the analysis of the gene expression profile during the early embryogenesis of *C. floridanum* may provide valuable information allowing the molecular mechanism of polyembryony in this insect to be elucidated.

Gene expression profiles provide a powerful basis upon which to clarify the molecular mechanisms of life phenomena in organisms without complete genomic information. However, in *C. floridanum*, a gene expression profile of the cleavage and the primary morula stages has been unavailable. To date, two cDNA collections of *C. floridanum* have been registered in GenBank, one of which is derived from polyembryos composed of proliferation-stage and early-morphogenesis-stage embryos (NCBI BioProject Accession: PRJNA65673); the other is derived from two types of larvae (normal reproductive larvae and precocious soldier larvae) by the suppression subtractive hybridization approach [12]. For detecting stage-specific genes during the early development of *C. floridanum*, these cDNA data may be helpful in excluding constitutive expression genes.

It has been reported that non-coding RNAs (ncRNAs) act as regulators of gene expression during embryogenesis [13]. Most known ncRNAs are as short as miRNAs and some of them induce the degradation of maternal RNAs [14], [15]. Long ncRNAs (lncRNAs) have also been identified in a large number of expressed sequence tags (ESTs) from several model organisms [16]–[20]. Therefore, to elucidate the molecular mechanisms of polyembryony, ncRNAs as well as protein-coding RNAs should be included in the analysis of gene expression during the early embryonesis of *C. floridanum*.

In the present study, we constructed full-length cDNA libraries of two consecutive embryonic stages of the wasp, during which polyembryogenesis is suspected to be initiated and processed. We evaluated the clones according to their similarities to known protein-coding and non-coding transcripts. Subsequently, clones of potential significance to polyembryogenesis were selected by comparing with cDNA collections and from the quantity of each clone in a library. Clones selected from both libraries were confirmed according to their expression patterns determined by RT-PCR and RT-qPCR. Here we describe the properties of stage-specific cDNA libraries and the developmental gene expression profile during the polyembryogenesis of a wasp.

## Methods

### Insects

The polyembryonic parasitic wasp *C. floridanum* and the host *Thysanoplusia intermixta* were reared at 22°C under a 16 h L–8 h D photoperiodic regime [21].

### RNA purification and cDNA library construction

Female wasps were allowed to lay eggs in 24–48 h-old host eggs for 30 min. Wasp RNAs were isolated from more than 50 cleavage-stage embryos (4–8 h post-parasitism) and from more than 50 primary morula-stage embryos (12–24 h post-parasitism). We used two different methods to avoid contamination with host RNAs. For the cleavage stage, the wasp eggs were dissected from host eggs and simply washed with culture medium (modified MGM-450 medium). For the primary morula stage, we cultured the wasp eggs from the cleavage stage singly and *in vitro* for development into the primary morula, according to the method of Iwabuchi (1995) [21]. Here, morula formation *in vitro* was recognized by the shedding of the egg chorion.

These RNA samples (each starting amount: 150 ng total RNA) were used for cDNA synthesis by long-distance PCR, according to the protocol of the SMART cDNA library construction Kit (TaKaRa). In cDNA construction, cDNAs were ligated into the pDNR-LIB vector and transformed into competent cells (competent high DH5α, TOYOBO). The transformants were plated on LB agar plates supplemented with chloramphenicol (30 µg/ml final concentration) and incubated overnight at 37°C. Colonies containing individual cDNA clones were picked up randomly and used as templates for colony-PCR with Emeraldamp MAX PCR premix with dye (TaKaRa) and the M13 primer set (supplied by SMART cDNA library construction Kit) for 25 cycles under the following PCR conditions: 98°C, 10 s; 50°C, 30 s; 72°C, 4 min. The PCR products were checked by electrophoresis on a 1% agarose gel. Colonies containing inserts of over 500 bps were chosen and cultured overnight at 37°C in 2 ml LB–chloramphenicol medium. The plasmid DNA was harvested using a PureYield Plasmid Miniprep System (Promega) and stored at −40°C until use. The clones were sequenced using the M13 forward or reverse primer on an ABI3100xl capillary sequencer.

### Sequencing and analysis

Our workflow is composed of three steps (Fig. S1): Step I is dedicated to sequence preprocessing and assembly, Step II is dedicated to annotation at the nucleotide and protein levels, and Step III is dedicated to a comparison with customized datasets. For the processing of all the BLAST outputs indicated below, Perl programs were written.

In Step I, all clones were sequenced in the 3′ to 5′ direction. When this was denied by a long poly (A) tail or other repeats of nucleotide, the clone was sequenced in the reverse direction. Low quality segments at the 5′ and 3′ cDNA ends and vector regions were removed from the individual sequence reads on the TraceEditor of MEGA5 [22]. Sequence reads, which only had repeats of nucleotides or significant similarity (E<10^−6^) to known rRNA as a result of BLASTN searches on Blast2GO [23], were also removed from libraries. The remaining clones in libraries were also sequenced in the reverse direction, and the raw sequences were processed as with the primary sequencing. Among pairs of bidirectional sequences from a single clone, a pair sharing a consensus region was assembled into a contiguous consensus sequence (contig) with the Alignment Explorer of MEGA5. Sequencing reads that had no consensus region with any other reads were classified as singletons. These contigs and singletons made up the dataset of our libraries.

In Step II, all sequences were evaluated with Blast2GO using the BLASTX algorithm. BLASTX hits were cut off at a threshold level of E-value = 10^−6^. For performance improvements, we installed a working database to run Blast2GO in our server, according to the installation manual on the Blast2GO web page (http://www.blast2go.com/b2ghome). All of the known ncRNA sequences, which had been registered up until July 2013, are compiled into a dataset from the Functional RNA database in the Functional RNA Project (http://www.ncrna.org/). Programs from BLAST+ [24] were used for comparative analyses of customized datasets using the BLASTN algorithm. Our sequences were compared with the ncRNA dataset by BLAST+ (cut-off point, E-value = 10^−6^).

In Step III, we made a multi-fasta file containing the formally registered sequences of *C.floridanum* transcripts; 230 ESTs derived from larvae [12] and 15183 TSA sequences derived from embryos at the morula-proliferation stage and early stage of morphogenesis (NCBI BioProject Accession: PRJNA65673, Smith et al., directly submitted 2011). To detect overlapping sequences with other cDNA collections, our sequences were compared with the dataset by BLAST+ using the BLASTN algorithm (cut-off point, E-value = 10^−6^). To detect clone duplication in our libraries, we also compared our sequences with themselves by BLAST+. Each resulting cluster of clone duplication was aligned using the CLUSTALW algorithm on the Alignment Explorer of MEGA5 using the default settings. Alignment errors were removed in order to allow a fit with the consensus regions indicated by the homology search. The multiple alignment file was used, as necessary, for the prediction of RNA secondary structures by CentroidFold, an online prediction service of the Functional RNA Project (http://www.ncrna.org/).

### RT-PCR and RT-qPCR

Total RNAs were isolated in order to determine the expression patterns of clones in three replicates at various stages and in tissues. RNA isolation was performed by using NucleoSpin RNA XS (TaKaRa). Embryos at varying developmental stages (1, 4, 8, 12, 16 and 24 h post-parasitism) were used. Here, primary morulae had been formed by 12 h post-parasitism. In order to examine whether the selected clones were deposited maternally, the heads and abdomens were dissected from the adults seven–eight days after egress from the host carcass and treated separately. All samples were stored at −80°C until used. RNA samples were purified and then reverse transcribed according to the protocol of the SuperScript VILO cDNA Synthesis Kit (Invitrogen) with random hexamers. RT-PCR was performed using the resulting cDNAs as templates with Emeraldamp MAX PCR premix with dye (TaKaRa) and gene-specific primers (Table S1) for 35 cycles under the following PCR conditions: 98°C, 10 s; 58°C, 30 s; 72°C, 90 s. Amplification D2 fragment of a 28S ribosomal gene, by using a previously reported primer set [25], was used as an endogenous control. Reverse transcription quantitative PCR (RT-qPCR) was performed using the same cDNA samples as templates with THUNDERBIRD SYBR qPCR Mix (TOYOBO) and gene-specific primers (Table S2). All runs were carried out using MiniOpticon (BIO-RAD) system and the data were analyzed using CFX Manager 3.1 (BIO-RAD) software. The parameters of RT-qPCR experiment for each target gene were shown in Supporting Information S1. For absolute quantification, calibration curves were established using a dilution series of all the selected clones and glyceraldehyde 3-phosphate dehydrogenase (*gapdh*). *gapdh* has been reported as a suitable reference gene with stable expression levels in different developmental stages, castes and tissues in hymenopteran insects [26], [27]. We determined full- length sequence of *C.floridanum gapdh* in this study (M0090) and used it for RT-qPCR as an endogenous reference gene. Results were normalized from the expression of *gapdh* transcript in the same sample and then illustrated as means±SDs of the expression ratios. All statistical analyses were performed using R version 2.13.0 [28].

## Results

### Library construction

We constructed stage-specific full-length cDNA libraries of embryos at the cleavage and primary morula stages. From over 3000 clones preselected from each library by colony PCR, 419 and 563 clones of the cleavage-stage and primary morula-stage embryos, respectively, were sequenced in one direction. By removing the clones that consisted of only nucleotide repeats or sequences corresponding to known rRNAs, we isolated 182 and 263 clones from cleavage-stage and primary morula-stage embryos, respectively. All of these clones were resequenced in the reverse direction. Pairs of bidirectional consensus sequences derived from single clones were assembled into contigs. As a result, we acquired 63 single sequences and 137 contigs from the cleavage-stage cDNA library and 331 single sequences and 82 contigs from the primary morula-stage library. The singletons were deposited in EST division (HX954220-HX954613) and the contigs in the HTC division (AK442469-AK442687) of the DDBJ database, to be used for subsequent analyses.

### Annotation of total clones by sequence similarity

**Figure 1:**
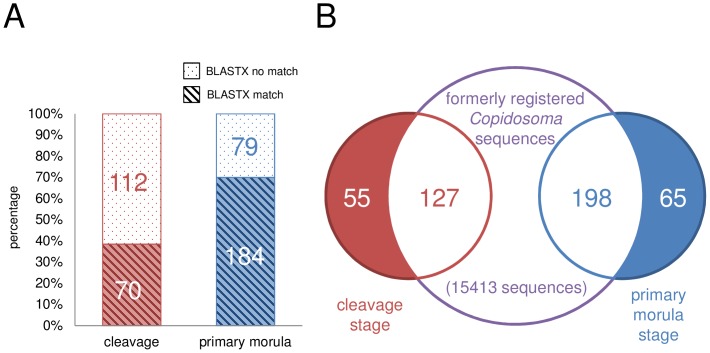
BLASTX analysis and comparative analysis. (A) Bar chart representing results of BLASTX analysis. The red bar indicates cleavage-stage clones with a BLASTX match (slant lines) or no match (dots), and the blue bar indicates primary morula-stage clones. Numbers indicate the number of clones represented by each bar. The primary morula-stage library (70%) contained far more clones which matched with known protein-coding genes than the cleavage-stage library (38%). (B) A Venn diagram showing the overlap of genes among three data sets (our two data sets from the cleavage and primary morula stages, and one set of registered data). The formerly registered sequence collection includes the proliferation stage and the early morphogenesis stage TSAs (JI831114–JI846296, NCBI BioProject Accession: PRJNA65673) and larval ESTs (DV181803–DV182032, Donnell and Strand, 2005) of *C.floridanum*. Each number indicates the number of clones contained within each intersection.

BLASTX analyses showed that 70 clones out of 182 isolated from cleavage-stage embryos and 184 clones out of 263 from the morula-stage embryos exhibited significant similarity to known protein-coding genes. Therefore, as many as 70% of the clones isolated in the primary morula-stage library, but only 38% in the cleavage-stage library matched with no known protein-coding genes (Fig. 1A). Most of those exhibiting siginificant similarity (approximately 80%) showed the best match to the sequences of hymenopteran genes. In particular, 61% matched with genes of the parasitoid, *Nasonia vitripennis*; however, only 5 clones (0.3%) showed matches with lepidopteran genes. In contrast, we found no significant similarities between all the isolated clones (182+263) from both the cleavage-stage and primary morula-stage libraries to known ncRNAs. The results of BLAST searches and GO mapping-annotation steps by Blast2GO are listed in Table S3–S6.

### Comparisons with formerly registered *C. floridanum* cDNAs

For comparative analysis, we compiled a dataset of previously registered *Copidosoma* sequences (15183 TSA sequences derived from polymorula and 230 EST sequences from larvae). The results of the comparison indicate that 127 clones of our cleavage-stage library and 198 of our primary morula-stage library overlapped with the sequences registered in the dataset (Fig. 1B, Table S4–S6). Of the 127 and 198 clones, seven clones including four ribosomal proteins (C0466, M0239, M3111, and M4940 in Table S6) were observed in all of these cDNA collections. The remaining 55 clones from the cleavage stage and 65 from the primary morula stage (Table S3) were considered stage-specific (Fig. 1B).

**Figure 2:**
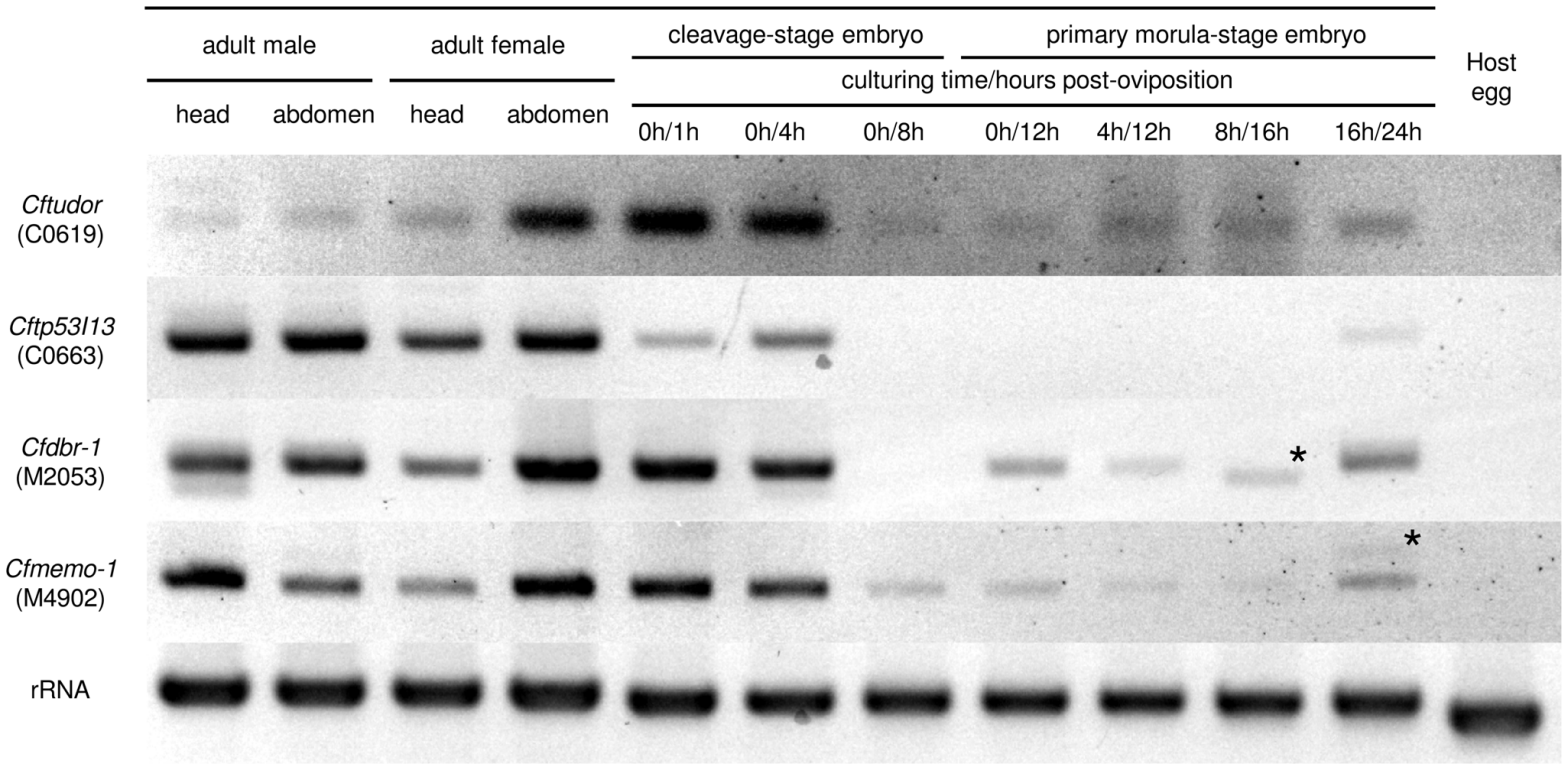
Results of RT-PCR for protein coding genes. Agarose gel electrophoresis of the RT-PCR products using primers (Table S1) specific for four clones (C0619, C0663, M2053 and M4902). All electrophoreses were performed on 3% agarose gel and run in an appropriate buffer. RNA templates were the total RNA fractions isolated from the heads or abdomens of the adults of both sexes (1st to 4th columns from the left), from embryos at various developmental stages (5th to 11th columns from the left), and from host eggs (12th column from the left). The samples from embryos less than 8 h post oviposition were taken from cleavage-stage embryos and the others from primary morula embryos, because primary morulae had been formed by 12 h post-parasitism. A 0 h culturing time corresponds with the sample from embryos having been immediately dissected from the host, and others indicate that samples were obtained from primary morula-stage embryos, which had been raised from cleavage-stage embryos *in vitro*. Amplification of a D2 fragment of the 28S ribosomal gene using a previously reported primer set [25] was used as an endogenous control. The gel images were cropped from original images, the color of which were inverted and auto-adjusted. For the original gel images, see in Figure S2. The label on the left-hand side of each row gives the name of clones used as a target. Unknown non-specific bands which could not be sequenced and reamplified were indicated by an asterisk.

**Figure 3:**
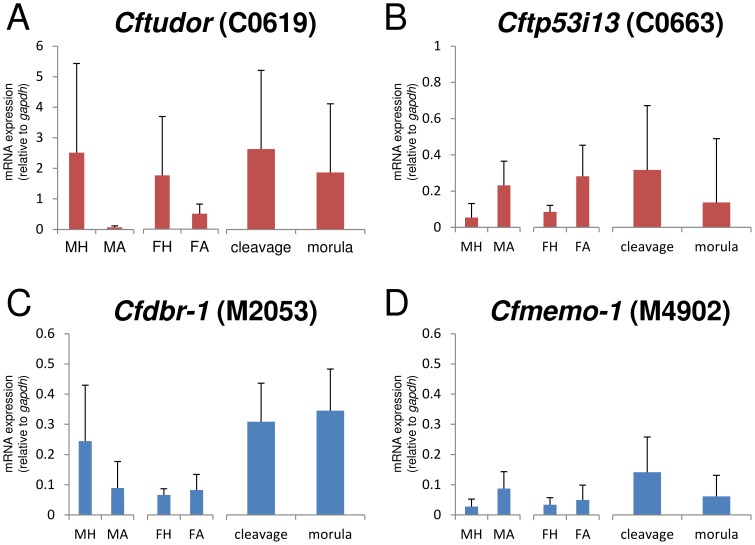
Results of RT-qPCR for protein coding genes. Gene specific primers for RT-qPCR were mentioned in Table S2. The detailes of PCR condition for each target gene were descrived in Supporting Information S1. (A)–(D): Bar graphs showing gene expression relative to *gapdh* (means; bars  =  S.D). MH: adult male head (n = 3), MA: adult male abdomen (n = 3), FH: adult female head (n = 3), FA; adult female abdomen (n = 3), cleavage: cleavage-stage embryo (1–8 h post-oviposition, n = 9), morula: primary morula-stage embryo (12–24 h post-oviposition, n = 12). No differences were determined to be statistically significant for any comparison of pairs at p = 0.05 (two-sample t-test or Welch's test).

Among the 55 stage-specific clones, some were found to correspond with protein-coding genes (Table S3). Among them, C0619 is similar to the maternal protein tudor, while others were proteins of uncertain function during development, such as C0663, which is similar to the tumor suppressor protein, p53 inducible protein 13 (TP53I13). Therefore, in this report, C0619 is named *Cftudor* and C0663, *Cftp53i13*. Subsequently, we characterized the expression of *Cftudor* and *Cftp53i13* by RT-PCR and RT-qPCR. Both clones had variable patterns of expression in all wasp samples, but were not expressed in host eggs (Fig. 2 & Fig. S2). The results of RT-qPCR showed that the clone *Cftudor* was more highly expressed than most of the other selected clones at both embryonic stages (Fig. 3A). *Cftudor* and *Cftp53i13* tended to be more highly expressed in cleavage-stage embryos (1–8 h post-oviposition) than in primary morula-stage embryos (12–24 h post-oviposition), but there was no significant difference in the expression ratios (Fig. 3A & Fig. 3B). In adults, both clones had varied expression patterns, but there were no significant differences between tissues (Fig. 3A & Fig. 3B). These RT-qPCR results present a high variability, especially in adult tissues (Fig. 3).

In the primary morula-stage-specific clones, we identified clones corresponding to many kinds of protein-coding genes, such as DNA repair proteins, signal transduction proteins, and transcription factor proteins (Table S3). From them we selected the following two clones for subsequent RT-PCR and RT-qPCR analyses: (1) M2053, which is similar to the gene coding RNA lariat debranching enzyme (DBR-1); (2) M4092, which is similar to the gene-coding mediator of cell motility (MEMO-1). Both M2053 (named here *Cfdbr-1*) and M4092 (named here *Cfmemo-1*) were expressed at both the cleavage and primary morula stages, but were not expressed in host eggs (Fig. 2 & Fig. S2). The results of qPCR showed that *Cfdbr-1* tended to be more highly expressed in morula-stage embryos than in cleavage-stage embryos (Fig. 3C). *Cfmemo-1* displayed comparatively low expression in all the PCR tests (Fig. 3D).

### Screening for frequency in cDNA libraries

**Figure 4:**
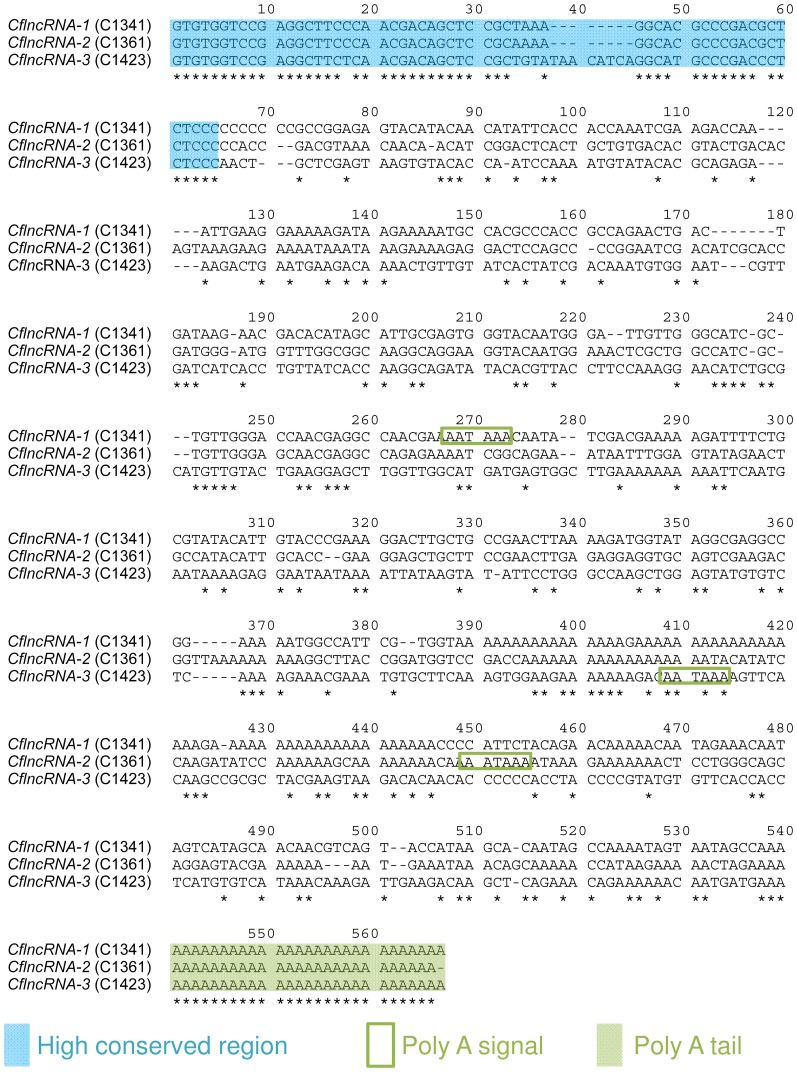
Alignment of selected protein non-coding RNAs (CflncRNAs) by CLUSTALW. The alignment of three sequences was performed on the Aligment Explorer of MEGA5 using the default settings. The conservation of nucleotides was indicated by an asterisk and dashes indicate gaps. The label on the left-hand side of each sequence gives the sequence name (a representational clone name). The alignment shows CflncRNAs have conserved regions among them (blue filled squares), poly A tails (filled green squares) and poly adenylation signals (AATAAA, green enclosure) at different points of each sequence.

**Figure 5:**
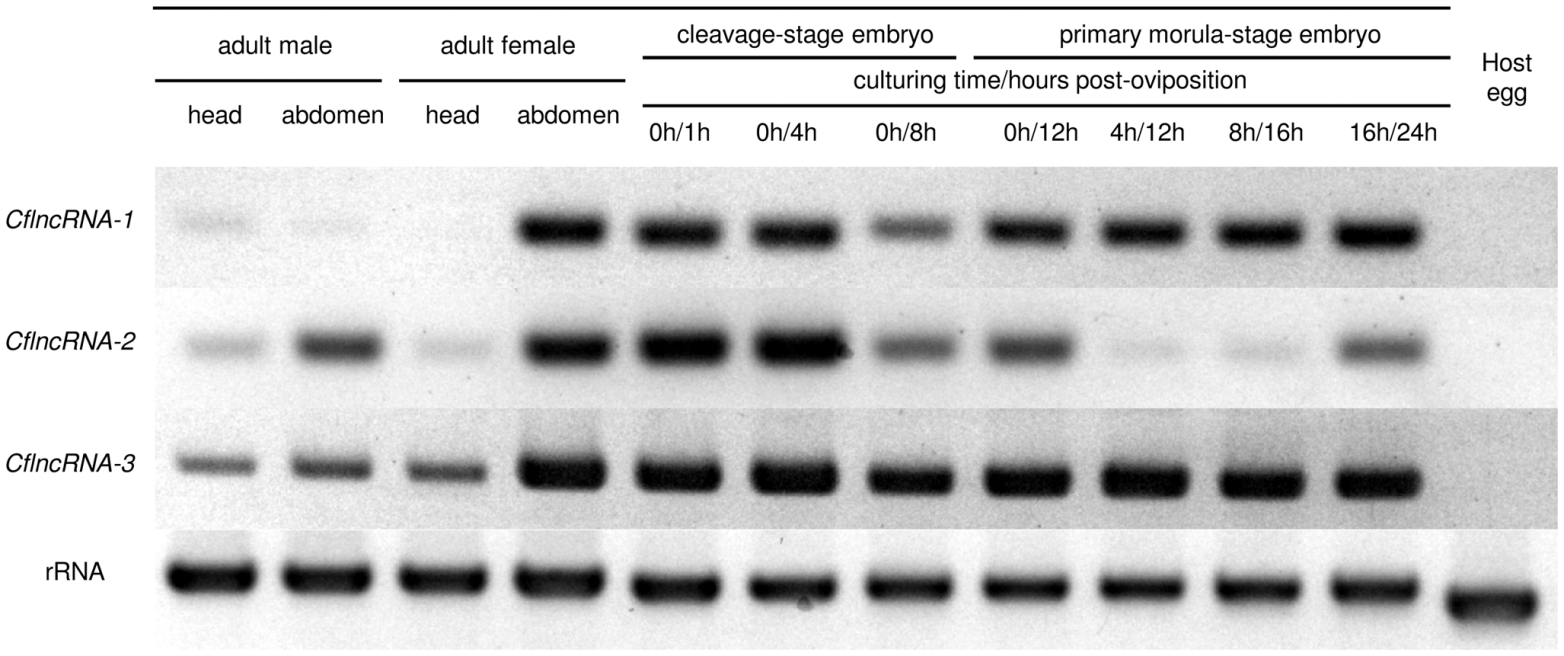
Results of RT-PCR for CflncRNAs. Agarose gel electrophoresis of RT-PCR products using primers (Table S1) specific for non-coding RNAs. All electrophoreses were performed on 3% agarose gel and run in an appropriate buffer. RNA templates were the total RNA fractions isolated from the heads or abdomens of the adults of both sexes (1st to 4th columns from the left), from embryos at various developmental stages (5th to 11th columns from the left), and from host eggs (12th column from the left). The samples from embryos less than 8 h post oviposition were taken from cleavage-stage embryos and the others from primary morula embryos, because primary morulae had been formed by 12 h post-parasitism. A 0 h culturing time corresponds with the sample from embryos having been immediately dissected from the host, and others indicate that samples were obtained from primary morula-stage embryos, which had been raised from cleavage-stage embryos *in vitro*. Amplification of a D2 fragment of the 28S ribosomal gene using a previously reported primer set [25] was used as an endogenous control. The gel images were cropped from original images, the color of which were inverted and auto-adjusted. For the original gel images, see in Figure S2. The label on the left-hand side of each row gives the name of the CflncRNAs used as a target.

**Figure 6:**
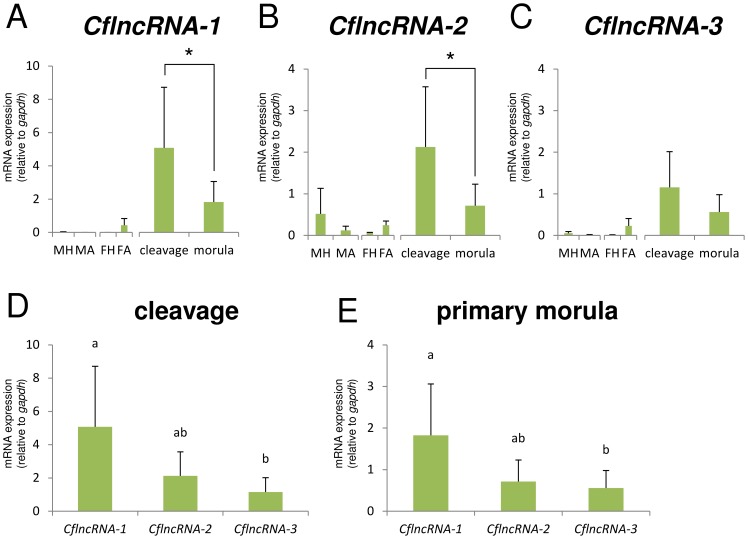
Results of RT-qPCR for CflncRNAs. Gene specific primers for RT-qPCR were mentioned in Table S2. The detailes of PCR condition for each target gene were descrived in Supporting Information S1. (A)–(C): Bar graphs showing the gene expression relative to *gapdh* (means, bars = S.D). MH: adult male head (n = 3), MA: adult male abdomen (n = 3), FH: adult female head (n = 3), FA; adult female abdomen (n = 3), cleavage: cleavage-stage embryo (1–8 h post-oviposition, n = 9), morula: primary morula-stage embryo (12–24 h post-oviposition, n = 12). For A and B, *indicates a statistically significant difference, p<0.05 (two-sample t-test). (D) and (E): Comparison of mRNA expression levels between the different CflncRNA variants in each embryonic stage. Bar graphs showing the gene expression relative to *gapdh* (means, bars  =  S.D). Bars allocated the same letters are not statistically different (p<0.05, Kruskal-Wallis analysis of variance).

To detect clone duplications, we compared all the clones in the two cDNA libraries. Results showed that 180 clones from both libraries were composed of 47 clusters. These clusters contained 34 pairs, eight triplets and five large clusters (Table 1 & Table S7). The largest cluster contained 58 clones, all of which had lost any similarities to any known protein-coding genes and long (>80 aa) open reading frames, suggesting that this cluster was composed of ncRNAs. This cluster was composed of three subclusters, including a highly conserved region and a variable region (Fig. 4). These sequences were termed CflncRNAs (*C. floridanum* long non-coding RNAs: *CflncRNA-1, CflncRNA-2* and *CflncRNA-3*). Representative sequences of each CflncRNA were tested in subsequent RT-PCR and RT-qPCR analyses. The results showed that all three of the CflncRNAs were not expressed in unparasitized host eggs (Fig. 5). The results of RT-qPCR showed that *CflncRNA-1* and *CflncRNA-3* were rarely observed in adult tissues, except for in the female abdomen (Fig. 6A & Fig. 6C). Furthermore, *CflncRNA-1* and *CflncRNA-2* were significantly more highly expressed in cleavage-stage embryos than in primary morula-stage embryos (Fig. 6A & Fig. 6B). Among the three ncRNAs, *CflncRNA-1* was comparatively more highly expressed at both embryonic stages (Fig. 6D & Fig. 6E). These RT-qPCR results present a high variability (Fig 6).

**Table 1:** Summary of screening for frequency in cDNA libraries.

rank	number of clones comprising clusters	number of clusters	sequence description by blast2go
1	58	1	not applicable
2	14	1	heat shock protein 70
3	7	1	not applicable
4	5	1	tubulin alpha-1b chain
5	4	1	polyadenylate-binding protein 1
6	3	8	*
7	2	34	*
sum	—	47	—

*For the details of two and more descriptions, see Table S7.

## Discussion

We successfully isolated 182 cDNA clones from the cleavage stage of *C. floridanum* embryos, and 263 from the subsequent primary morula stage. Most of the clones showed high similarities to hymenopteran protein-coding sequences. However, only a few of the clones showed relatively high similarities to both hymenopteran and lepidopteran sequences. These gene similarities might have been established in the wasps due to evolutionary interactions between the parasite and their lepidopteran hosts. The results, however, also suggest that, with the isolation techniques used at both the cleavage and primary morula stages, contamination with host lepidopteran RNAs was successfully prevented. In contrast, one fifth of all registered ncRNAs were identified from the fly, *Drosophila melanogaster*. However, no homologous ncRNA was detected in our sequences, even if the E-value cut-off for BLAST searches was changed from 10^−6^ to 10^−0^ (data not shown). Due to the higher mutation rates of ncRNAs than of protein-coding RNAs [29], it is hard to detect predicted ncRNAs from homology searches between evolutionarily distant species.

To compare the stage-specific clones here, the gene expression profiles in *C. floridanum* have been constructed from registered data of the polyembryos (NCBI BioProject Accession: PRJNA65673) and the larvae [12]. Comparisons with the existing data identified 55 stage-specific clones at the cleavage stage, and 65 at the subsequent morula stage.

Developing a molecular approach based on EST libraries implied that only a few targets were identified, compared with that based on RNA-seq data. Other very important molecular codings may be unlikely to be identified due to the limitations of EST libraries. To compensate for this bias in transcriptomic quantitative information, we performed real-time RT-qPCR analysis together with a simple RT-PCR method. The real-time RT-PCR technique provided information about precise relative expression by comparing it to the expression of a determined reference gene. Simple RT-PCR is useful for detecting the important variations observed in high intensity bands, but not adequate for detecting quantifiable accurate expression.

Two clones were chosen from the cleavage-stage-specific clones for the detailed characterization of expression by RT-PCR and RT-qPCR analyses. The results of RT-PCR analysis show that *Cftudor* (C0619) and *Cftp53i13* (C0663) were transcribed by *C. floridanum* due to their absence from the host egg (Fig. 2 & Fig. S2). The results of RT-qPCR indicated that the expression levels of *Cftudor* were comparatively high (about twice that of *gapdh*) at both embryonic stages (Fig. 3A). Therefore, wasp eggs may hold *Cftudor* in large amounts for early embryogenesis. In *Drosophila*, mRNA of *tudor* is maternally deposited in an egg for polar granule assembly and germ cell formation, and localized within only 2 h post-oviposition with an expression gradient [30], [31]. Because template RNA for RT-PCR was derived from a whole embryo and gene expression ratios were normalized from the expression levels of *gapdh*, the relative expression level of a gene was declined if expression was restricted to a small part of an embryo. *Cftudor* is possibly not localized, at least by primary morula formation, because there was no significant difference between the expression ratios in cleavage- and primary morula-stage embryos (Fig. 3A). However, the expression of *Cftp53i13* in the female abdomen was equal to that in cleavage-stage embryos (Fig. 3B). Therefore, *Cftp53i13* (C0663) may be maternally deposited in the wasp egg. In *C. floridanum*, early embryogenesis is potentially controlled by various previously unreported maternal factors.

In contrast to the cleavage-stage library, the primary morula-stage-specific clones mainly comprise various protein-coding genes. Two clones were chosen: *Cfdbr-1* (M2053) and *Cfmemo-1* (M4902). *Cfdbr-1* is similar to DBR-1, which has been reported to be related to RNA processing [32], [33] and essential for embryogenesis in *Arabidopsis thaliana* [34]. *Cfmemo-1* is similar to MEMO-1, which is required for cell migration in animals as it relays chemotactic signals [35]. Although the expression of these genes was observed throughout both the embryonic stages investigated, expression was not detected in host eggs (Fig. 2 & Fig. S2), suggesting our libraries to be highly accurate. As a result of qPCR, *Cfdbr-1* (M2053) tends to be up-regulated during the primary morula stage (Fig. 3C), suggesting that it is of potential importance during polyembryogenesis. *Cfmemo-1* (M4902) showed comparatively low expression in the primary morula-stage embryo (Fig. 3D). This suggests it has site-specificity and is closely related to polyembryogenesis, e.g., controlling the invagination of the syncytial extraembryonic membrane of the morula into the embryonic cell mass.

A high variability of the qPCR results in adult tissues may be originated mostly from sample conditions (very small samples and a hard cuticle of adults), but not because of age variability (emergence of adults in the host carcass is simultaneous in a single host). Because of very small size of insects (ca. 1 mm), the quantity of total RNAs isolated were too low to apply to quality assessment. A trace elements of RNA samples are likely to show differential degradation of individual mRNAs. A hard cuticle in adults may also cause variation in RNA isolation efficiency.

Our previous studies have indicated that the primary morula formed from an egg laid in the yolk of the host egg secondarily invades the host embryo [36], [37]. The primary morula actively invades the host embryo by extending the extraembryonic syncytial membrane and penetrating between the host's epithelial cells. In this study, two novel odorant receptors (M4960 and M5761 in Table S3) have been detected in morula-stage specific clones. It remains unclear whether *C. floridanum* morulae orient towards the host embryo before host invasion. Along with novel odorant receptors, *Cfmemo-1* may be required for host invasion during polyembryogenesis.

We did not normalize the current libraries so that we could retain a positive correlation between the number of clones in the library and the levels of gene expression. Using the sequence clustering and alignment method, we detected novel lncRNAs, named CflncRNA, in our libraries, which showed three patterns of paralogs or splicing variants (Fig. 4). Several lncRNAs are known to play essential roles during embryogenesis [38]–[41]. In insect embryogenesis, it has been confirmed that miRNAs [42], [43] and mRNA-like lncRNAs [29] show different expression patterns in specific tissues and cell types, but the roles of these ncRNAs have not yet been determined. In our comparison, CflncRNA expression varied; in particular, *CflncRNA-1* was specific to our libraries (Table 2), regardless of it being expressed most highly among three libraries of both embryonic stages (Fig. 6D & Fig. 6E). In addition, RT-qPCR showed that the expression pattern of *CflncRNA-1* was similar to that of *CflncRNA-3* in adult tissues (Fig. 6A & Fig. 6C), although phylogenetic analysis using the alignment represented in Fig. 4 showed that *CflncRNA-1* and *CflncRNA-2* were most closely-related (data not shown). These results suggest that CflncRNAs have different functions. *CflncRNA-1* may be responsible for cleavage- and morula-stage-specific phenomena. *CflncRNA-2* is possibly related to site-specific phenomena in the primary morula, while *CflncRNA-3* expression may be constantly required during the whole process of embryogenesis. To date the suggestion has been that the translation of many genes, including TP53I13 [44] and MEMO-1 [45], is regulated by miRNAs and that several miRNAs are encoded by a single lncRNA [46], [47]. Predictions of the secondary structure of RNAs from the multiple sequence alignments show that all of the CflncRNAs contain several double-stranded regions (Fig. S3). These double-stranded regions possibly work independently and play a role as regulators of stage-specific genes during polyembryogenesis in *C. floridanum*. Further investigations aimed at identifying the location or interaction targets of the CflncRNAs may lead to the discovery of the genes responsible for polyembryogenesis. We also believe that the cDNA sequence data in the present study will be extremely valuable to future investigations by aiding RNA-seq read mappings, because of the higher mutation rates [29], isoforms and paralogs of ncRNAs greatly decreasing the accuracy of sequence assembly. Using the sequence clustering and alignment methods, we also detected lncRNAs in cDNA collectons of other hymenoptera. The largest cluster contained 27 clones, which could be classified into three patterns of lncRNA isoforms (Fig. S4) in the library derived from the venom gland of the parasitic wasp, *Meteorus pulchricornis* (accession numbers FY736475–FY736909, Sano et al., direct submission in 2011). The results suggest that other insects have varying patterns of ncRNA, which are affected by gene duplication or alternative processing, and that the ncRNAs may show tissue-specificity.

**Table 2:** Summary of features of CflncRNAs in each cDNA collection.

lncRNAs	percentage (number of clones/total)		duplication with formary registered cDNA collection* ^1^ (number of sequences/total)	
	cleavage-stage	primary morula-stage	proliferation-stage and early- morphogenesis stage* ^2^	larval stage* ^3^
*CflncRNA-1*	12.0 (22/182)	2.3 (6/263)	no (0/15183)	no (0/230)
*CflncRNA-2*	2.7 (5/182)	0.8 (2/263)	yes (2/15183)	no (0/230)
*CflncRNA-3*	11.5 (21/182)	0.8 (2/263)	yes (1/15183)	no (0/230)

*1: no - absence of a consensus sequence (query cover>90%, e-value<10-6) in the collection, yes - presence of a concensus sequence.

*2: Accession: JI831114–JI846296 (NCBI BioProject Accession: PRJNA65673).

*3: Accession: DV181803–DV182032 [12].

We conclude that the clones in our libraries are efficient for the elucidation of the underlying molecular mechanisms of polyembryogenesis in *C. floridanum*. We characterized four candidates for protein-coding clones and three novel lncRNAs by RT-PCR and RT-qPCR. This is the first time that the relationship between lncRNA and polyembryogenesis has been reported. These genes may be good candidates for controllers of embryo development and polyembryony in *C. floridanum*. Further analyses of the localization of the associated mRNAs and/or the protein products will be useful for gaining a full understanding of the roles of these genes. Among the seven candidates, *CflncRNA-1* is the one with the most interesting expression profile, because it is almost exclusively expressed during the cleavage and primary morula stages.

## Supporting Information

Figure S1 **Schematic representation of sequencing and analysis in this study.**
(PDF)

Figure S2 **Original gel images including molecular size markers.**
(PDF)

Figure S3 **The RNA secondary structure of CflncRNAs predicted by CentroidFold.** The heat color gradation from blue to red on each predicted base-pair corresponds to the base-pairing probability from 0 to 1.
(PDF)

Figure S4 **Alignment of three ESTs in the largest cluster of venom grand ESTs of** ***M. pulchricornis*** **by clustalW.** ESTs of *M. pulchricornis* were collected from INSDC data base (accessions FY736475–FY736909, Sano et al., direct submission in 2011). The ESTs were compared with itself and the 27 ESTs of the resulting largest cluster were aligned. The result of alignment showed the ESTs in the largest cluster were classifiable as three sequence patterns (pattern 1: FY736509, FY736510, FY736527, FY736574, FY736649, FY736678, FY736688, FY736706, FY736734, FY736790, FY736855, FY736890; pattern 2: FY736556, FY736563, FY736573, FY736593, FY736638, FY736862, FY736906; pattern 3: FY736606, FY736653, FY736682, FY736747, FY736790, FY736793, FY736796, FY736893). The ESTs of alignment were omitted except for three and showed in this. The alignment of three sequences was performed on the Aligment Explorer of MEGA5 using the default settings. The conservation of nucleotides was indicated by an asterisk and dashes indicate gaps. The label on the left-hand side of each sequence gives an accession number of representational ESTs. The predicted lncRNAs of *M. pulchricornis* have highly conserved region among them but not stable open coding flame.
(PDF)

Table S1 **Listing of primers used for RT-PCR in this study.**
(PDF)

Table S2 **Listing of primers used for RT-qPCR in this study.**
(PDF)

Table S3 **Classification of stage-specific sequences derived from** ***C. floridanum*** **embryos.**
(PDF)

Table S4 **Classification of sequences derived from from** ***C. floridanum*** **embryos and duplicated with the formerly registered cDNA sequences derived from proliferation-stage and early- morphogenesis stage.**
(PDF)

Table S5 **Classification of sequences derived from from** ***C. floridanum*** **embryos and duplicated with the formerly registered cDNA sequences derived from larvae.**
(PDF)

Table S6 **Classification of sequences derived from from** ***C. floridanum*** **embryos and duplicated with all of the cDNA collections used for comparison.**
(PDF)

Table S7 **Listing of clones in the clusters as a result of screening for frequency.**
(PDF)

Supporting Information S1 **The run reports of RT-qPCR experiments for each target gene.**
(PDF)
