# Supplementary figures and images for: Gene Expression of Protein-Coding and Non-Coding RNAs Related to Polyembryogenesis in the Parasitic Wasp, Copidosoma floridanum
Source: PLoS One. 2014 Dec 3;9(12):e114372. doi: 10.1371/journal.pone.0114372 (PMC4255003; doi:10.1371/journal.pone.0114372)

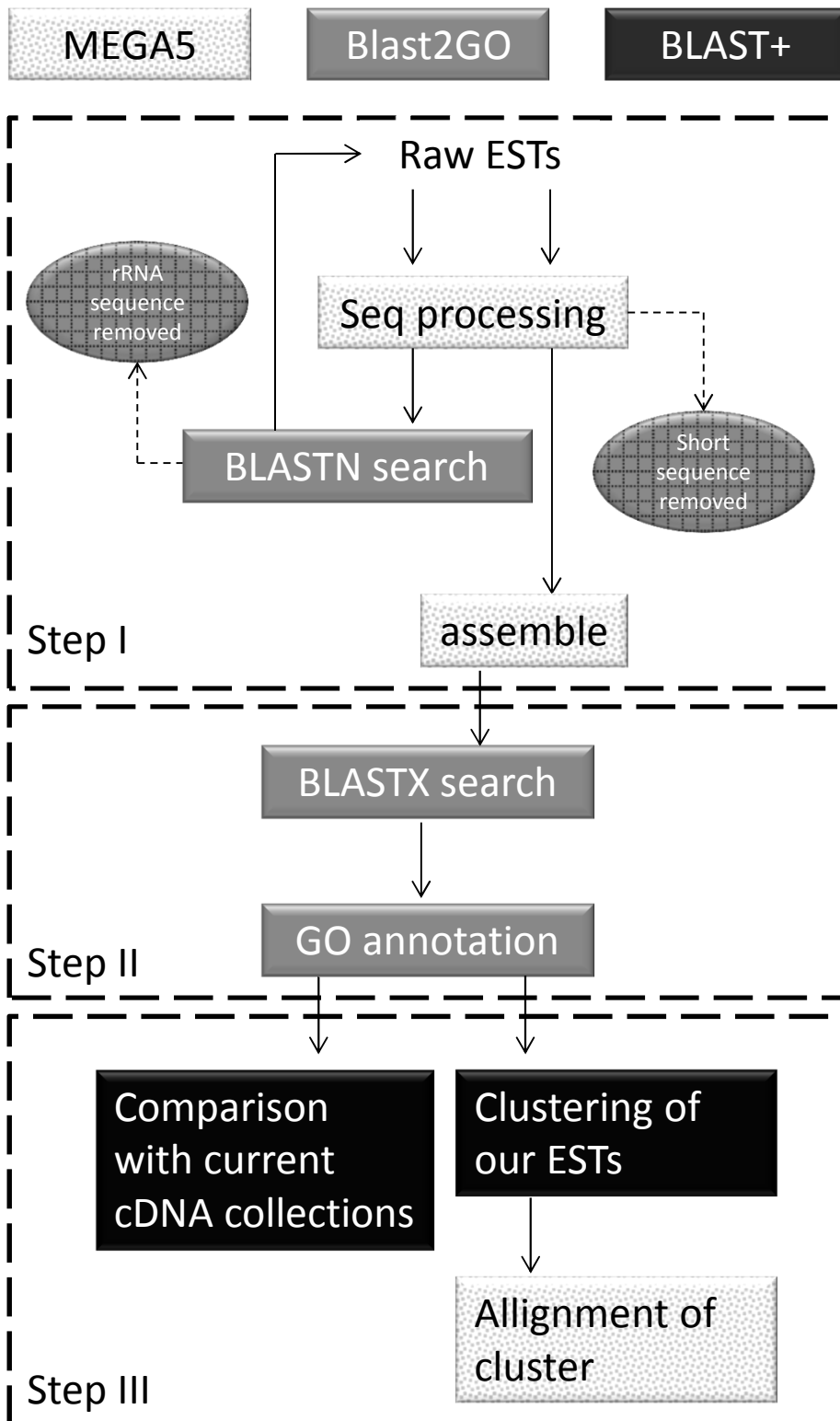

Figure S1

Supplement: Figure S1 — Schematic representation of sequencing and analysis in this study. (PDF) [file pone.0114372.s001.pdf]

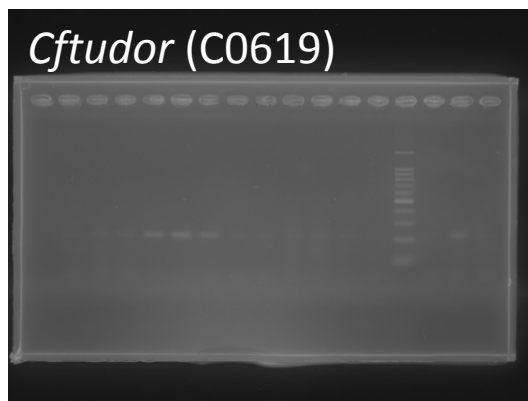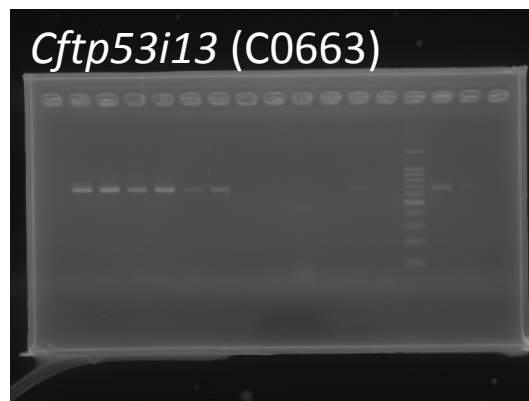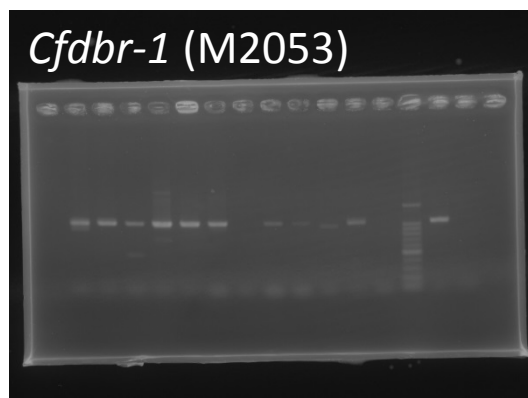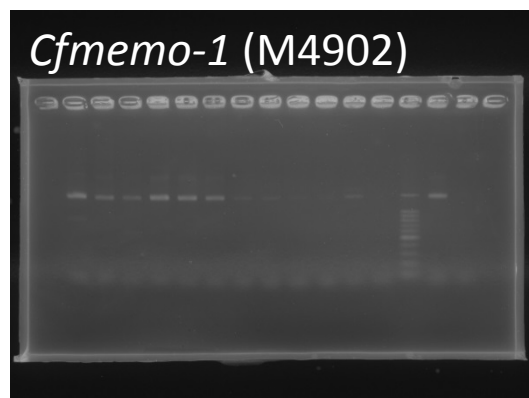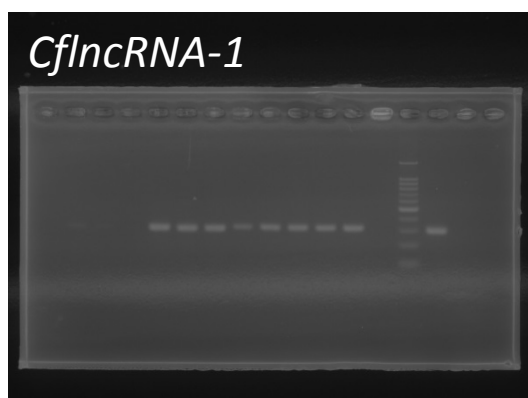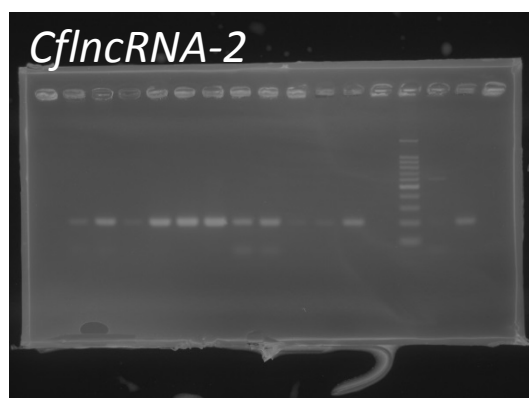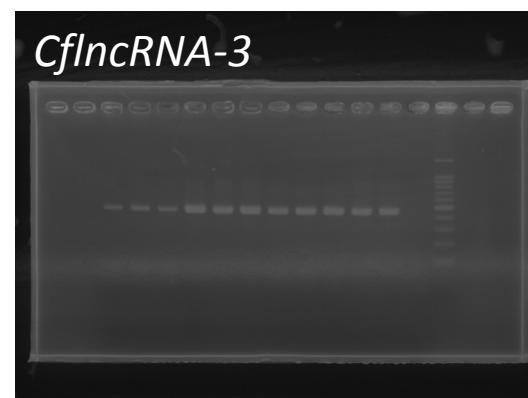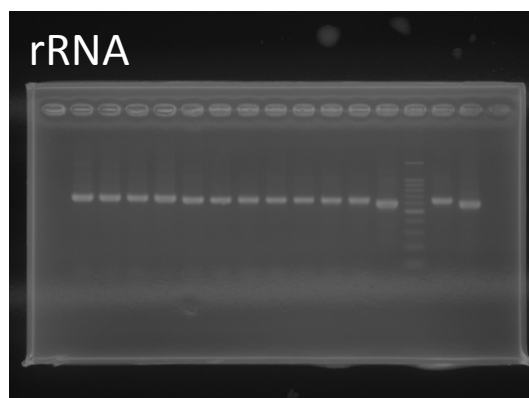

PCR test
 
 another experiment

size marker

Figure S2

Supplement: Figure S2 — Original gel images including molecular size markers. (PDF) [file pone.0114372.s002.pdf]

### *CflncRNA-1*

0.0 1.0

RT-qPCR primer

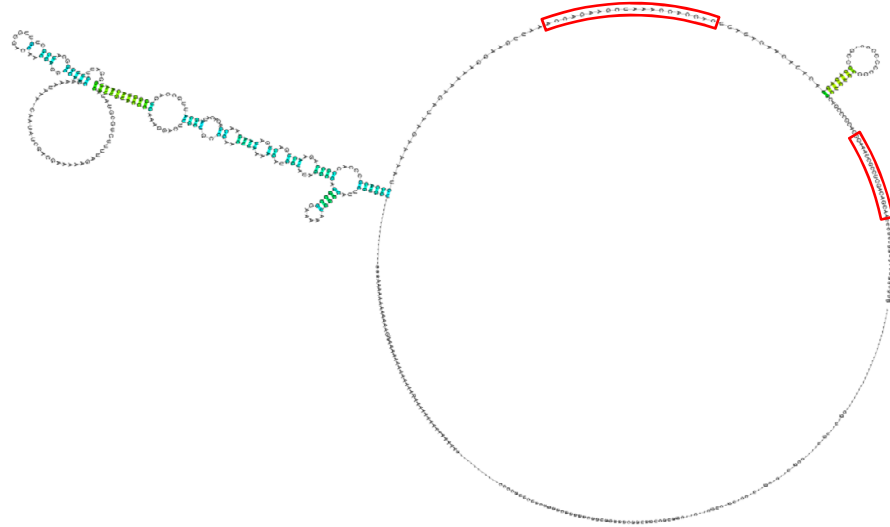

### *CflncRNA-2*

0.0 1.0

RT-qPCR primer

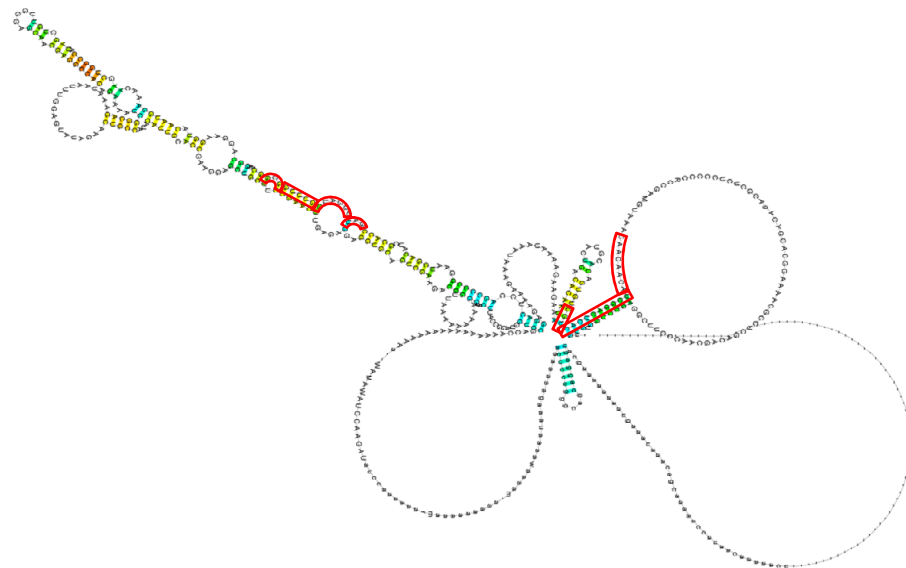

### *CflncRNA-3*

0.0 1.0

RT-qPCR primer

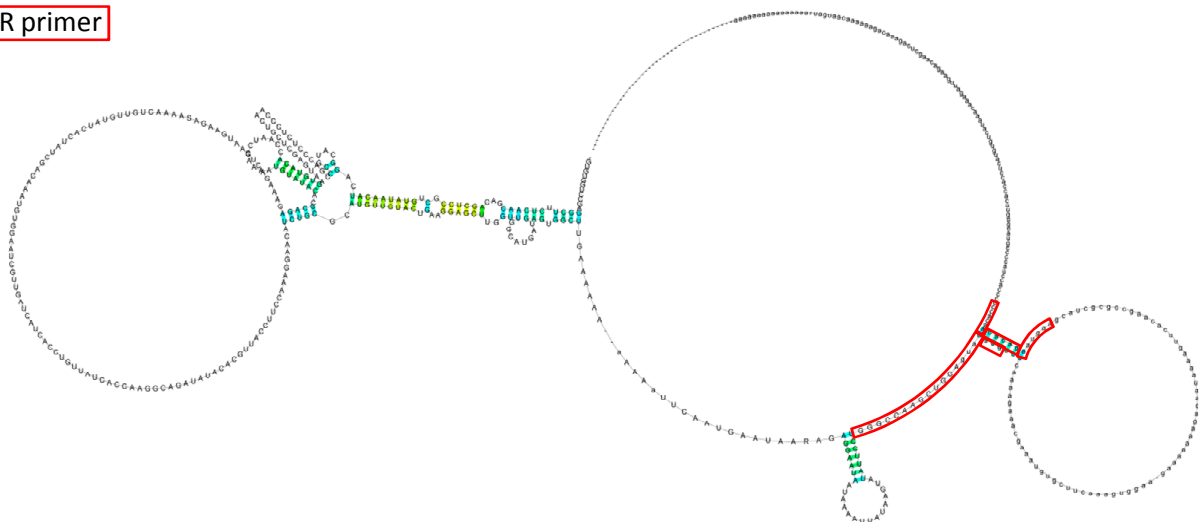

Figure S3

Supplement: Figure S3 — The RNA secondary structure of CflncRNAs predicted by CentroidFold. The heat color gradation from blue to red on each predicted base-pair corresponds to the base-pairing probability from 0 to 1. (PDF) [file pone.0114372.s003.pdf]
