# Supplementary material for: Gene Expression of Protein-Coding and Non-Coding RNAs Related to Polyembryogenesis in the Parasitic Wasp, Copidosoma floridanum
Source: PLoS One. 2014 Dec 3;9(12):e114372. doi: 10.1371/journal.pone.0114372 (PMC4255003; doi:10.1371/journal.pone.0114372)
Supplement: Figure S4 — Alignment of three ESTs in the largest cluster of venom grand ESTs of M. pulchricornis by clustalW. ESTs of M. pulchricornis were collected from INSDC data base (accessions FY736475–FY736909, Sano et al., direct submission in 2011). The ESTs were compared with itself and the 27 ESTs of the resulting largest cluster were aligned. The result of alignment showed the ESTs in the largest cluster were classifiable as three sequence patterns (pattern 1: FY736509, FY736510, FY736527, FY736574, FY736649, FY736678, FY736688, FY736706, FY736734, FY736790, FY736855, FY736890; pattern 2: FY736556, FY736563, FY736573, FY736593, FY736638, FY736862, FY736906; pattern 3: FY736606, FY736653, FY736682, FY736747, FY736790, FY736793, FY736796, FY736893). The ESTs of alignment were omitted except for three and showed in this. The alignment of three sequences was performed on the Aligment Explorer of MEGA5 using the default settings. The conservation of nucleotides was indicated by an asterisk and dashes indicate gaps. The label on the left-hand side of each sequence gives an accession number of representational ESTs. The predicted lncRNAs of M. pulchricornis have highly conserved region among them but not stable open coding flame. (PDF) [file pone.0114372.s004.pdf]

```
                  10         20         30         40         50         60
FY736678  GACATTGAAA TTCAAAATGT TAGCGTACTA TTTTCTCGCT ATTTGTGTGG TGGGACCTGT
FY736796  GACATTGAAA TACAAAATGT TGGCATACTA TTTTTTTCCT ATTTTCGTGG TGGGACCTCT
FY736573  GACATTGAAA TTCAAAATGT TGGCATACTA TTTCGTCGCT ATTTTTGTGG TGGGACCTCT
          ********** * ******** * ** ***** ***  *  ** ****  **** ******** *

                  70         80         90        100        110        120
FY736678  CATCTTCATG ACTGAAGTGA TGGGCGCTGG ATCGACCGGA AAAACTGAAA CTACTAGTAC
FY736796  CATCATGACG ACTAAAGTGA TGGGCG---- ---------- -------TAG CACCGACTGA
FY736573  CATCTTGACA ACTGAAGTGG TGGGCC---- ---------- -------AGA CATCAAGCAC
          **** * *   *** *****  *****                            *  * *

                 130        140        150        160        170        180
FY736678  TACTACTACT ACTACAGAAA TTTCCAGAAC CGATTTGGGA GGAAAAATTA CGGGCATTAC
FY736796  AACACCTGCT GACGGCGTGG CT---AGGGC C--------A TCGACAATCT G----CCAAA
FY736573  AGCGATTCCC GATGACAAAA T----AGAAT CAA----AGA TCAATAATTT G----ACTGG
            *   * *                  **    *        *    * ***

                 190        200        210        220        230        240
FY736678  GATATACGAT ACTGGTAAAA TTGAAGACAT TAAACAATTG CTGAAACATA TTTGGCAAAG
FY736796  TCCTTTTAAC GCCGAGAAGA TAAAAGAC-- -------GCC TTGAACTTCT TGTTAGACAG
FY736573  AACATACGCC TCGGATTTAA CCGCAGCC-- -AAACAAGTG CTGAAAGATT TCTTACTGAA
              *       * *     *     ** *               ****      * *     *

                 250        260        270        280        290        300
FY736678  AATTAGAAAT GACAAAACTG CCATATCAAA AAATCTTACA GTATCAGAGC AAATCGGCAA
FY736796  AATTGATAAT GACAGAAAGA ACCTAACACA AATAGCTGAA GACATTAACC AAGGATCGGA
FY736573  AATTGAAAAT GATAGATTGA ACATAACAAA GTTACATGAA GCATCGAAAA AAGCATTGAA
          ****   *** ** * *      * ** ** *       *  * *      *   **       *

                 310        320        330        340        350        360
FY736678  TCAGTCTGTA ATATTAGAAA AAGTTGCAAA TAGCGCGCAT ATAAAACTGA GCGATGTAGT
FY736796  GAAGTCGGAA GCAGTAAAGA AGATTACAGA TGATTTACTG AAAGAATTAG GCGAAGCAGC
FY736573  CGAATTAGCA AATGCAGAGG TGGTATCAAA CAACCTTATC AAACCAGTAA AGGAGGCATT
            * *  * *      * *      *  ** *            * *  * *     ** * *

                 370        380        390        400        410        420
FY736678  AAGTGACGTA CATCGCGCAA AAGGAGAGGC AGAAAGTGCT TTGACTTCTG GTAAGGCAGC
FY736796  ACAAAAATCA GGTTATGCAG AAGCTCAGGC AAAAAGTGCA TCAATTCTTA TTGCAAAAGC
FY736573  GGTAGATGCG ACTATCGCAA AGGGACAGGG AAGTAATGCA ATGGCTAGTG GAATAAACGC
               *       *   ***  * *   ***  *   * ***       *  *          **

                 430        440        450        460        470        480
FY736678  AGAAGCTAAA GCTAAAGTTG CATCAGACGA GGCTGTTAAA GCACGGAAAA AGGCGAGAGA
FY736796  AGAAGAACCT GCTGCATACG CAGCGGCAGC TGCAAAAACA GCACAGTTTG AAGCAGAAGA
FY736573  AGAGGTGCTA GCTGCAGATG TTGCTGCATC TGCAAAGGCA GCAGAAGATA GAGCAAGAAC
          *** *      ***  *   *    * *       **      * ***          **   *

                 490        500        510        520        530        540
FY736678  CGCATTTAAA AACAGCTCTT CTGTCACTAT ATTTGCATAC GCATACTTAT ATCTCGTTAT
FY736796  GTCTAACAGG CGCAACTCTG GTGTCGTCAC ATCACCATAC GCATACCTAT ATACTTTCAT
FY736573  AGCTTATGAG CGCAGCTCTG GTGTCACCAC ATCACCATAC ACATACTTAC ATCTATTTAT
            *          ** ****   ****   *  **   *****  ***** **  **    * **

                 550        560        570        580        590        600
FY736678  GTGTATGTAT GTATTTCTGT ATATTTAACT GATATACAAA TT---TACAT GTACATGGAA
FY736796  ATGTGTGTAT GCTTATCTTT ATATTTAACC GGAAACTAAA ATAGTTTCAT GTACTTCTAC
FY736573  ATGTGTATAT TTTTATCTAT ATATTTAACT GATATATAGT CA---TACAT GGATACAAAT
           *** * ***    * *** * *********  *  *   *        * *** * *     *
```

High conserved region

Figure S4
