# Supplementary material for: Gene Expression of Protein-Coding and Non-Coding RNAs Related to Polyembryogenesis in the Parasitic Wasp, Copidosoma floridanum
Source: PLoS One. 2014 Dec 3;9(12):e114372. doi: 10.1371/journal.pone.0114372 (PMC4255003; doi:10.1371/journal.pone.0114372)
Supplement: Table S1 — Listing of primers used for RT-PCR in this study. (PDF) [file pone.0114372.s005.pdf]

Table S1 Listing of primers used for RT-PCR in this study.

Primer name	Primer sequence (5'–3')	Amplicon size (bp)
Cftudor (C0619)		
C0619F	ATC CAA CTA TCG GAC GAG GG	223
C0619R	GCC GTT CTT CCG TTA TCT CG	
Cftp53i13 (C0663)		
C0663F0	ATC CTA GTG GGA CGC TCC TTT G	671
C0663R	GGA GTG ATA ATG TGC CTG CG	
Cfdbr-1 (M2053)		
M2053F	GCA TGG GAG TGA AAC AGC AA	1053
M2053R2	GGT AAG CTC TCA TCG TGT TCT ACC TC	
Cfmemo-1 (M4902)		
M4902F	CCC TTT CAA CTG CTA CGG TC	1485
M4902R	CAC AGT ACG TTT AGG CGC TG	
CflncRNA-1		
CflncRNA-1F	AAC GAC AGC TCC GCW AAA	290*1
CflncRNA-1R	TAA GTT CGG CAG CAA GTC CT	
CflncRNA-2		
CflncRNA-2F	CCC GCC GAA GTA AAC AAC AA	197*2
CflncRNA-2R	ATT TTC TCT GGC CTC GTT GC	
CflncRNA-3		
CflncRNA-3F	AGG CTT CTC AAC GAC AGC	451*3
CflncRNA-3R2	GGT GGT GAA CAC ATA CGG GGT A	
rRNA D2*4		
D2-3551 F	CGT GTT GCT TGA TAG TGC AGC	640*5
D2-4057 R	TCA AGA CGG GTC CTG AAA GT	593*6

*1 in case of using the clone C1341 as template
*2 in case of using the clone C1361 as template
*3 in case of using the clone C1423 as template
*4 cited in Gillespie et al., [25]
*5 in case of using samples derived from *C. floridanum* (wasp) as template
*6 in case of using samples derived from *T. intermixta* (host) as template
