# Supplementary material for: Gene Expression of Protein-Coding and Non-Coding RNAs Related to Polyembryogenesis in the Parasitic Wasp, Copidosoma floridanum
Source: PLoS One. 2014 Dec 3;9(12):e114372. doi: 10.1371/journal.pone.0114372 (PMC4255003; doi:10.1371/journal.pone.0114372)
Supplement: Table S2 — Listing of primers used for RT-qPCR in this study. (PDF) [file pone.0114372.s006.pdf]

Table S2 Listing of primers used for RT-qPCR in this study.

Primer name	Primer sequence (5'–3')	Amplicon size (bp)
Cftudor (C0619)		
C0619rtF	CGG ACG AGG GTG CTA CTA TTT T	148
C0619rtR	GCA ACG ACC TCA TCT TCC ACT T	
Cftp53i13 (C0663)		
C0663rtF2	GGT AAT GGA TCA CAT CCT GGT AG	149
C0663rtR2	CTG AGG TTA GGT CTT GTC ATT TTC C	
Cfdbr-1 (M2053)		
M2053rtF2	TTA CTC CAA GCG AAG AGG AAA A	120
M2053rtR2	CGT GTT TAG TGG GTG TTT GAG G	
Cfmemo-1 (M4902)		
M4902rtF2	TGA TTG GAT GGA TAT GGA TAC TG	158
M4902rtR2	GTT TCC CAT ACA TAG CTT CAC GTT C	
CflncRNA-1		
CflncRNA-1rtF	AAC GAC AGC TCC GCT AAA GG	89*1
CflncRNA-1rtR	TGG TCT TCG ATT TGG TGG TG	
CfincRNA-2		
CflncRNA-2rtF	CAA CAA CAT CGG ACT CAC TGC	115*2
CflncRNA-2R	ATT TTC TCT GGC CTC GTT GC	
CflncRNA-3		
CflncRNA-3rtF1	GGG CCA AGC TGG AGT ATG TG	109*3
CflncRNA-3R	GGG TGT TGT GTC TTA CTT CG	
gapdh (M0090)		
M0090rtF2	GCG AGC GGT TCT ATC ATT CC	130
M0090rtR2	CTC AGC ACC AGA CGA CCA ATA C	

*1 in case of using the clone C1341 as template
*2 in case of using the clone C1361 as template
*3 in case of using the clone C1423 as template
