# Supplementary material for: Gene Expression of Protein-Coding and Non-Coding RNAs Related to Polyembryogenesis in the Parasitic Wasp, Copidosoma floridanum
Source: PLoS One. 2014 Dec 3;9(12):e114372. doi: 10.1371/journal.pone.0114372 (PMC4255003; doi:10.1371/journal.pone.0114372)
Supplement: Table S3 — Classification of stage-specific sequences derived from C. floridanum embryos. (PDF) [file pone.0114372.s007.pdf]

Table S3 Classification of stage-specific sequences derived from *C. floridanum* embryos.

Sequence Name	Accession Number	Sequence Description	Min. eValue	Mean Similarity	Gene ontology terms*
cleavage-stage library					
C0052	AK442470	not applicable			-
C0098f	HX954404	probable palmitoyltransferase zdhhc16-like	9.41E-38	71.00%	F:zinc ion binding
C0098r	HX954221	probable palmitoyltransferase zdhhc16-like	1.00E-81	75.30%	F:zinc ion binding; F:transferase activity
C0182	AK442475	not applicable			-
C0222	AK442479	PREDICTED: hypothetical protein LOC100679333	8.15E-116	57.38%	-
C0311	AK442485	not applicable			-
C0359	AK442489	not applicable			-
C0380	AK442490	not applicable			-
C0452	AK442492	not applicable			-
C0544f	HX954412	protein suppressor of hairy wing-like	3.27E-115	82.60%	F:metal ion binding; F:nucleic acid binding
C0570	AK442500	not applicable			-
C0573f	HX954413	not applicable			-
C0580	AK442503	not applicable			-
C0581	AK442504	not applicable			-
C0597	AK442509	not applicable			-
C0606	AK442512	not applicable			-
C0610	AK442513	forkhead-associated protein	4.47E-13	57.90%	P:regulation of cellular process; P:cellular process; F:catalytic activity
C0613	AK442514	not applicable			-
C0617	AK442516	not applicable			-
C0619	AK442517	maternal protein tudor	2.49E-19	55.80%	-
C0627f	HX954414	not applicable			-
C0629f	HX954415	not applicable			-
C0629r	HX954225	not applicable			-
C0630	AK442520	not applicable			-
C0635	AK442522	not applicable			-
C0640f	HX954416	protein suppressor of hairy wing-like	1.77E-114	82.60%	F:metal ion binding; F:nucleic acid binding
C0658	AK442533	not applicable			-
C0662	AK442534	not applicable			-
C0663	AK442535	tumor protein p53-inducible protein 13	5.83E-124	62.80%	-
C0681	AK442540	not applicable			-
C0706	AK442544	not applicable			-
C0721	AK442545	not applicable			-
C0734	AK442546	not applicable			-
C0874f	HX954424	josephin-like protein	2.74E-84	87.10%	F:omega peptidase activity
C0907f	HX954425	not applicable			-
C1017f	HX954428	PREDICTED: nicalin-1-like	6.20E-08	81.00%	-
C1107r	HX954230	not applicable			-
C1138	AK442557	not applicable			-
C1166	AK442559	not applicable			-
C1189	AK442561	not applicable			-
C1214f	HX954430	ap2-associated protein kinase 1	1.66E-11	91.50%	P:protein phosphorylation; F:ATP binding; F:protein serine/threonine kinase activity

* P: biological process, C: cellular component, F: molecular function

Table S3 continued

Sequence Name	AccessionNumber	Sequence Description	Min. eValue	Mean Similarity	Gene ontology terms*
cleavage-stage library					
C1214r	HX954231	not applicable			-
C1259	AK442566	nck-interacting protein with sh3 domain-like	2.60E-66	76.60%	P:regulation of transcription, DNA-dependent; P:translational initiation; F:translation initiation factor activity; C:nucleus
C1266f	HX954432	chromobox protein 5	1.34E-20	75.88%	C:nucleus
C1270f	HX954433	not applicable			-
C1270r	HX954233	not applicable			-
C1311	AK442575	not applicable			-
C1317	AK442576	not applicable			-
C1354	AK442580	not applicable			-
C1375	AK442583	not applicable			-
C1376	AK442584	not applicable			-
C1415	AK442590	not applicable			-
C1435	AK442592	not applicable			-
C1436	AK442593	not applicable			-
C1507	AK442596	not applicable			-
C1606f	HX954443	not applicable			-
C1631	AK442602	not applicable			-
C2118	AK442604	not applicable			-
C2119f	HX954445	not applicable			-
primary morula-stage library					
M0063	AK442612	dna repair protein complementing xp-a cells homolog	9.48E-61	60.70%	P:regulation of cellular process; P:cellular response to stimulus
M0073	AK442614	not applicable			-
M0075f	HX954449	ccr4-not transcription complex subunit 10-like	1.30E-21	62.10%	-
M0075r	HX954244	ccr4-not transcription complex subunit 10-like	3.06E-56	66.80%	P:mRNA metabolic process; P:gene expression; C:intracellular part
M0076f	HX954450	pol-like protein	2.17E-43	52.20%	F:RNA binding; P:RNA-dependent DNA replication; F:RNA-directed DNA polymerase activity; F:nucleic acid binding; F:ribonuclease H activity; F:zinc ion binding
M0081	AK442615	dappu_58732-like	6.75E-49	68.85%	F:binding
M0089	AK442619	not applicable			-
M0103	AK442621	not applicable			-
M0167f	HX954453	disco-interacting protein 2-like	1.25E-80	94.00%	F:catalytic activity; P:metabolic process
M0167r	HX954247	not applicable			-
M0231f	HX954457	protein smg9-like	8.06E-51	76.73%	-
M0300f	HX954463	tld domain-containing protein kiaa1609	9.10E-71	70.63%	F:structural molecule activity; C:tight junction
M0300r	HX954256	not applicable			-
M0314f	HX954465	protein asteroid-like	2.13E-62	71.45%	F:copper ion binding; P:larval somatic muscle development; P:imaginal disc-derived wing vein morphogenesis; P:nucleic acid metabolic process; P:compound eye photoreceptor fate commitment; F:neuropeptide receptor binding; F:hormone activity; F:catalytic activity; P:neuropeptide signaling pathway; P:epidermal
M0314r	HX954258	protein asteroid-like	3.06E-13	62.40%	F:metal ion binding
M0416f	HX954476	not applicable			-

* P: biological process, C: cellular component, F: molecular function

Table S3 continued

Sequence Name	AccessionNumber	Sequence Description	Min. eValue	Mean Similarity	Gene ontology terms*
primary morula-stage library					
M0416r	HX954268	PREDICTED: hypothetical protein LOC100678247	4.11E-52	53.83%	-
M0592f	HX954482	mediator of rna polymerase ii transcription subunit	6.99E-86	75.45%	P:regulation of transcription, DNA-dependent; P:transcription, DNA-dependent; C:nucleus
M0592r	HX954274	mediator of rna polymerase ii transcription subunit	2.33E-86	68.85%	P:regulation of transcription, DNA-dependent; P:transcription, DNA-dependent; C:nucleus
M0622f	HX954483	not applicable			-
M0622r	HX954275	not applicable			-
M0890f	HX954487	not applicable			-
M0890r	HX954279	not applicable			-
M0902f	HX954488	arf gtpase-activating protein git2-like	6.97E-49	83.85%	P:regulation of ARF GTPase activity; F:ARF GTPase activator activity; F:zinc ion binding
M0902r	HX954280	not applicable			-
M0903r	HX954281	not applicable			-
M0921f	HX954490	mitochondrial dicarboxylate carrier-	1.41E-92	80.70%	P:transmembrane transport; C:integral to membrane
M0946f	HX954491	probable serine threonine-protein kinase ddb_g0283337-like isoform x2	1.63E-46	59.50%	-
M0946r	HX954283	not applicable			-
M1022f	HX954493	upf0586 protein c9orf41 homolog	2.60E-66	74.45%	F:molecular_function; P:biological_process; C:cellular_component
M1349f	HX954496	protein king tubby-like	1.81E-64	68.21%	-
M1349r	HX954287	not applicable			-
M1430f	HX954499	not applicable			-
M1430r	HX954290	sex-determining protein fem-1	1.13E-13	67.85%	-
M1621f	HX954501	rna pseudouridylate synthase domain-containing protein 2-like	7.05E-59	90.55%	F:pseudouridine synthase activity; P:riboflavin biosynthetic process; F:RNA binding; P:pseudouridine synthesis; F:diaminohydroxyphosphoribosylaminopyrimidine deaminase activity
M1621r	HX954293	not applicable			-
M1880f	HX954505	not applicable			-
M1880r	HX954299	PREDICTED: hypothetical protein LOC100680082	3.28E-10	76.00%	-
M1932f	HX954510	cyclin-k	4.35E-29	81.20%	P:regulation of cyclin-dependent protein serine/threonine kinase activity; F:protein kinase binding; P:regulation of transcription, DNA-
M1932r	HX954303	not applicable			-
M1943f	HX954512	protein lethal giant larvae-like	1.91E-44	78.95%	-
M1943r	HX954305	protein lethal giant larvae-like	3.79E-25	81.25%	-
M1945f	HX954513	not applicable			-
M1945r	HX954306	not applicable			-
M2053	AK442636	lariat debranching enzyme	0	72.85%	F:hydrolase activity, acting on ester bonds; P:mRNA processing

* P: biological process, C: cellular component, F: molecular function

Table S3 continued

Sequence Name	AccessionNumber	Sequence Description	Min. eValue	Mean Similarity	Gene ontology terms*
primary morula-stage library					
M2062f	HX954524	PREDICTED: hypothetical protein LOC100677809	2.22E-17	76.00%	-
M2062r	HX954318	PREDICTED: hypothetical protein LOC100677809	2.29E-24	68.10%	P:nucleus-associated proteasomal ubiquitin-dependent protein catabolic process; P:proteasome localization; C:nucleus
M3131	AK442641	not applicable			-
M3141	AK442642	not applicable			-
M3143f	HX954534	not applicable			-
M3143r	HX954329	inactive ubiquitin carboxyl-terminal hydrolase 54	2.15E-88	68.05%	F:hydrolase activity
M3211	AK442645	structural maintenance of chromosomes protein 5-like	6.45E-56	69.75%	P:DNA repair
M4028f	HX954537	not applicable			-
M4036	AK442647	not applicable			-
M4123f	HX954542	serine threonine-protein kinase plk4	3.66E-92	88.95%	F:protein tyrosine kinase activity; P:protein phosphorylation; F:ATP binding; F:protein serine/threonine kinase activity; C:centriole
M4123r	HX954336	not applicable			-
M4268f	HX954547	not applicable			-
M4268r	HX954341	not applicable			-
M4326	AK442652	not applicable			-
M4392f	HX954552	adenylyltransferase and sulfurtransferase mocs3-like	1.38E-72	82.40%	F:nucleotidyltransferase activity; C:cytosol; P:Mo-molybdopterin cofactor biosynthetic process; F:thiosulfate sulfurtransferase activity; F:metal ion binding; P:tRNA processing; F:ATP
M4411f	HX954554	not applicable			-
M4411r	HX954349	glycerophosphocholine phosphodiesterase gpcpd1-like	1.17E-49	83.15%	P:glycerol metabolic process; F:glycerophosphodiester phosphodiesterase activity; P:lipid metabolic process; F:starch
M4688f	HX954565	PREDICTED: hypothetical protein LOC100120723	2.04E-09	55.00%	-
M4688r	HX954359	helicase senataxin	1.98E-56	62.00%	F:hydrolase activity
M4729f	HX954566	cell division cycle protein 27 homolog	2.40E-26	53.00%	C:anaphase-promoting complex; P:cell division; P:anaphase-promoting complex-dependent proteasomal ubiquitin-dependent protein catabolic process
M4736f	HX954567	not applicable			-
M4736r	HX954360	not applicable			-
M4745	AK442657	rpii140-upstream gene	2.27E-35	65.30%	P:multicellular organismal development
M4767f	HX954569	not applicable			-
M4767r	HX954362	not applicable			-
M4808	AK442660	apolipoprotein d-like	1.59E-36	50.20%	F:pigment binding; C:extracellular region; F:lipid binding; P:transport; F:transporter
M4902	AK442663	protein memo1-like	1.68E-175	86.55%	-
M4934	AK442667	not applicable			-
M4939	AK442668	not applicable			-
M4948	AK442672	not applicable			-
M4960f	HX954580	odorant receptor 48	1.44E-10	60.00%	-
M4960r	HX954371	odorant receptor 48	2.64E-35	50.14%	-
M4989f	HX954584	not applicable			-

* P: biological process, C: cellular component, F: molecular function

Table S3 continued

Sequence Name	AccessionNumber	Sequence Description	Min. eValue	Mean Similarity	Gene ontology terms*
primary morula-stage library					
M4989r	HX954375	not applicable			-
M5155f	HX954593	not applicable			-
M5155r	HX954381	not applicable			-
M5162r	HX954382	nadh dehydrogenase subunit 1	6.80E-10	70.00%	P:oxidation-reduction process; C:mitochondrion; F:oxidoreductase activity; C:integral to membrane; C:membrane; F:NADH dehydrogenase (ubiquinone) activity
M5203	AK442677	not applicable			-
M5206f	HX954596	josephin-like protein	7.77E-92	85.55%	F:omega peptidase activity
M5206r	HX954385	not applicable			-
M5207f	HX954597	transposable element tc3 transposase	1.84E-11	54.85%	-
M5207r	HX954386	not applicable			-
M5213f	HX954598	not applicable			-
M5213r	HX954387	not applicable			-
M5288f	HX954603	not applicable			-
M5288r	HX954392	not applicable			-
M5340	AK442682	not applicable			-
M5497f	HX954607	solute carrier family 35 member e1 homolog	1.73E-46	82.95%	C:integral to membrane
M5497r	HX954396	not applicable			-
M5761f	HX954611	odorant receptor 48	5.01E-16	61.00%	-
M5761r	HX954400	odorant receptor 48	9.25E-18	61.00%	-
M5764	AK442687	not applicable			-
M5835f	HX954612	atp-dependent rna helicase ddx54-like	4.47E-88	86.90%	F:ATP-dependent helicase activity; F:RNA binding; F:ATP binding; C:nucleus
M5835r	HX954401	not applicable			-
M5952f	HX954613	not applicable			-
M5952r	HX954402	not applicable			-

* P: biological process, C: cellular component, F: molecular function
