# Supplementary material for: Gene Expression of Protein-Coding and Non-Coding RNAs Related to Polyembryogenesis in the Parasitic Wasp, Copidosoma floridanum
Source: PLoS One. 2014 Dec 3;9(12):e114372. doi: 10.1371/journal.pone.0114372 (PMC4255003; doi:10.1371/journal.pone.0114372)
Supplement: Table S4 — Classification of sequences derived from from C. floridanum embryos and duplicated with the formerly registered cDNA sequences derived from proliferation-stage and early- morphogenesis stage. (PDF) [file pone.0114372.s008.pdf]

Table S4 Classification of sequences derived from from *C. floridanum* embryos and duplicated with the formerly registered cDNA sequences derived from proliferation-stage and early- morphogenesis stage[*1].

Sequence Name	AccessionNumber	Sequence Description	Min. eValue	Mean Similarity	Gene ontology terms[*2]
cleavage-stage library					
C0017	AK442469	not applicable			-
C0056	AK442471	not applicable			-
C0057	AK442472	ras-related protein rab-1a-like	1.59E-134	90.65%	P:negative regulation of dendrite morphogenesis; P:protein secretion; P:actin filament organization; P:regulation of cell shape; P:small GTPase mediated signal transduction; P:regulation of cell migration; C:vesicle; P:cell adhesion; P:ER to Golgi vesicle-mediated transport; C:neuronal cell body; F:GTPase
C0086	AK442473	cob yrinic acid -diamide mitochondrial-	2.19E-93	70.40%	F:transferase activity
C0092f	HX954403	transcriptional repressor protein yy1-	5.57E-101	83.35%	F:metal ion binding; F:nucleic acid binding
C0092r	HX954220	transcriptional repressor protein yy1-	1.07E-26	93.30%	F:metal ion binding; F:nucleic acid binding
C0170f	HX954405	tubulin alpha-1b partial	3.47E-94	98.60%	P:protein polymerization; C:cytoplasmic microtubule; P:GTP catabolic process; P:microtubule-based process; F:GTPase activity; F:structural constituent of cytoskeleton; F:GTP
C0177	AK442474	prefoldin subunit 2	4.77E-63	81.90%	P:protein folding; C:prefoldin complex; F:unfolded protein binding; C:intracellular membrane-bounded organelle
C0185	AK442476	not applicable			-
C0211	AK442477	not applicable			-
C0220	AK442478	not applicable			-
C0244f	HX954406	26s protease regulatory subunit 6b	5.12E-68	87.95%	C:cytoplasm; F:peptidase activity; P:protein catabolic process; F:ATP binding; F:microtubule-severing ATPase activity; C:proteasome complex; P:proteolysis; C:nucleus
C0259	AK442481	not applicable			-
C0277	AK442482	not applicable			-
C0293	AK442483	not applicable			-
C0297	AK442484	not applicable			-
C0324	AK442486	ferritin light chain	2.02E-11	73.10%	P:single-organism process; F:transition metal ion binding
C0329	AK442487	replication protein a 14 kda subunit	9.47E-61	61.90%	C:cytoplasm
C0352	AK442488	nadh dehydrogenase	3.33E-18	62.40%	-
C0389	AK442491	not applicable			-
C0422f	HX954407	histone deacetylase	3.07E-51	97.25%	P:histone H4 deacetylation; F:NAD-dependent histone deacetylase activity (H3-K18 specific); P:regulation of transcription, DNA-dependent; F:NAD-dependent histone deacetylase activity (H3-K14 specific); P:histone H3 deacetylation; P:transcription, DNA-dependent; F:NAD-dependent histone deacetylase activity (H4-K16 specific); F:NAD-dependent histone deacetylase activity (H3-K9 specific); C:nucleus

*1 JI831114–JI846296 (NCBI BioProject Accession: PRJNA65673)

*2 P: biological process, C: cellular component, F: molecular function

Table S4 continued

Sequence Name	AccessionNumber	Sequence Description	Min. eValue	Mean Similarity	Gene ontology terms*
cleavage-stage library					
C0422r	HX954222	histone deacetylase rpd3	1.27E-19	85.54%	P:histone H4 deacetylation; F:NAD-dependent histone deacetylase activity (H3-K18 specific); P:regulation of transcription, DNA-dependent; F:NAD-dependent histone deacetylase activity (H3-K14 specific); P:histone H3 deacetylation; P:transcription, DNA-dependent; F:NAD-dependent histone deacetylase activity (H4-K16 specific); F:NAD-dependent histone deacetylase activity (H3-K9 specific); C:nucleus
C0430f	HX954408	isoform a	4.60E-23	82.15%	F:hydrogen-exporting ATPase activity, phosphorylative mechanism; P:ATP hydrolysis coupled proton transport; C:integral to membrane; P:ATP synthesis coupled proton transport; F:lipid binding; C:mitochondrial proton-transporting ATP synthase complex,
C0430r	HX954223	not applicable			-
C0439f	HX954409	dna-directed rna polymerase iii subunit rpc9	2.73E-42	73.00%	F:DNA-directed RNA polymerase activity; P:response to stimulus; P:single-organism cellular process; F:nucleic acid binding; P:transcription from RNA polymerase III promoter; F:G-protein coupled receptor activity; F:nucleotide binding; C:DNA polymerase III
C0451f	HX954410	heat shock protein 70	1.88E-102	94.45%	F:ATP binding; P:response to stress
C0455	AK442493	something about silencing protein 10-	9.76E-26	84.85%	-
C0456	AK442494	not applicable			-
C0505f	HX954411	histone	2.65E-60	98.00%	C:nucleoplasm; C:polytene chromosome; P:regulation of cell shape; P:osteoblast differentiation; P:cell adhesion; C:integral to membrane; F:oxidoreductase activity, acting on the CH-CH group of donors; C:extracellular region; C:nucleosome; C:cytoplasm; F:protein heterodimerization activity; P:regulation of centromere complex assembly; P:brain development; P:blood coagulation; P:lipid metabolic process; F:DNA binding; P:nucleus organization; P:male meiosis; P:muscle cell differentiation; P:oxidation-reduction process;
C0505r	HX954224	not applicable			-
C0524	AK442496	superoxide dismutase	3.60E-87	87.70%	F:metal ion binding; P:superoxide metabolic process; F:superoxide dismutase activity; F:peroxidase activity; P:oxidation-reduction
C0530	AK442497	cytochrome b-c1 complex subunit mitochondrial-like	2.26E-31	72.00%	C:mitochondrial inner membrane; F:ubiquinol-cytochrome-c reductase activity; C:respiratory chain; P:mitochondrial electron transport, ubiquinol to cytochrome c
C0531	AK442498	not applicable			-
C0569	AK442499	not applicable			-
C0578	AK442501	PREDICTED: hypothetical protein LOC100122415	5.62E-08	66.00%	-
C0579	AK442502	PREDICTED: hypothetical protein LOC100122415	5.55E-08	66.00%	-

* P: biological process, C: cellular component, F: molecular function

Table S4 continued

Sequence Name	AccessionNumber	Sequence Description	Min. eValue	Mean Similarity	Gene ontology terms*
cleavage-stage library					
C0586	AK442505	not applicable			-
C0587	AK442506	not applicable			-
C0594	AK442507	not applicable			-
C0596	AK442508	not applicable			-
C0598	AK442510	not applicable			-
C0600	AK442511	not applicable			-
C0615	AK442515	not applicable			-
C0621	AK442518	not applicable			-
C0628	AK442519	not applicable			-
C0633	AK442521	not applicable			-
C0636	AK442523	dna-directed rna polymerases and iii subunit rpabc5	9.08E-39	97.80%	P:transcription elongation from RNA polymerase III promoter; P:transcription initiation from RNA polymerase II promoter; P:7-methylguanosine mRNA capping; P:positive regulation of type I interferon production; F:DNA-directed RNA polymerase activity; F:zinc ion binding; F:protein kinase activity; P:transcription-coupled nucleotide-excision repair; P:termination of RNA polymerase III transcription; C:cytosol; P:protein phosphorylation; C:DNA-directed RNA polymerase II, core complex; F:DNA binding; P:positive regulation of viral transcription; P:viral process; P:mRNA splicing, via spliceosome; P:transcription elongation from
C0637	AK442524	not applicable			-
C0638	AK442525	not applicable			-
C0642	AK442526	transmembrane protein 258	1.56E-20	92.15%	C:integral to membrane
C0643	AK442527	not applicable			-
C0644	AK442528	atp synthase subunit mitochondrial	1.77E-50	77.10%	F:hydrogen-exporting ATPase activity, phosphorylative mechanism; P:ATP synthesis coupled proton transport; C:lipid particle; C:mitochondrial proton-transporting ATP synthase complex, coupling factor F(o)
C0645	AK442529	not applicable			-
C0646	AK442530	not applicable			-
C0649	AK442531	not applicable			-
C0651	AK442532	not applicable			-
C0665	AK442536	not applicable			-
C0666	AK442537	not applicable			-
C0669	AK442538	not applicable			-
C0672	AK442539	not applicable			-
C0675f	HX954417	baculoviral iap repeat-containing protein 6	5.52E-73	75.80%	F:acid-amino acid ligase activity
C0682	AK442541	not applicable			-
C0701	AK442542	not applicable			-
C0703	AK442543	eukaryotic translation initiation factor 1a	1.63E-67	98.00%	P:mitotic spindle elongation; F:translation initiation factor activity; P:translational initiation; C:cytosol; P:smoothened signaling pathway; C:eukaryotic 43S preinitiation
C0758f	HX954418	caax prenyl protease 1 homolog	3.05E-26	61.75%	F:peptidase activity
C0765	AK442547	not applicable			-

* P: biological process, C: cellular component, F: molecular function

Table S4 continued

Sequence Name	AccessionNumber	Sequence Description	Min. eValue	Mean Similarity	Gene ontology terms*
cleavage-stage library					
C0794f	HX954419	PREDICTED: hypothetical protein LOC100678844	2.39E-16	80.00%	-
C0794r	HX954226	PREDICTED: hypothetical protein LOC100678844	4.94E-67	58.33%	-
C0798	AK442548	not applicable			-
C0806	AK442549	not applicable			-
C0809f	HX954420	heavy subunit	2.66E-49	68.75%	P:single-organism process; F:transition metal ion binding
C0809r	HX954227	not applicable			-
C0810	AK442550	not applicable			-
C0829f	HX954421	not applicable			-
C0829r	HX954228	not applicable			-
C0844f	HX954422	heavy subunit	1.16E-53	65.75%	F:metal ion binding
C0844r	HX954229	not applicable			-
C0851f	HX954423	ubiquitin-conjugating enzyme e2 g1-like	2.14E-120	97.75%	F:DNA-directed DNA polymerase activity; P:transcription, DNA-dependent; F:DNA binding; P:protein ubiquitination; F:ubiquitin-protein ligase activity
C0879	AK442551	not applicable			-
C0908f	HX954426	bag family molecular chaperone regulator 2-	3.49E-58	78.60%	F:kinase activity; P:phosphorylation
C0911f	HX954427	bladder cancer-associated	1.70E-48	90.75%	F:molecular_function; P:biological_process; C:cellular_component
C0941	AK442552	not applicable			-
C0946	AK442553	not applicable			-
C1019	AK442554	not applicable			-
C1020	AK442555	not applicable			-
C1112f	HX954429	prohibitin protein wph	3.27E-83	91.35%	C:membrane
C1123	AK442556	not applicable			-
C1146	AK442558	PREDICTED: hypothetical protein LOC100114386	1.76E-31	61.36%	-
C1182	AK442560	regulator of chromosome	5.25E-55	77.75%	-
C1191	AK442562	not applicable			-
C1201	AK442563	not applicable			-
C1208	AK442564	atp synthase subunit mitochondrial	3.26E-64	82.00%	C:proton-transporting ATP synthase complex, catalytic core F(1); P:ATP synthesis coupled proton transport; F:proton-transporting ATPase activity, rotational mechanism; F:proton-transporting ATP synthase activity, rotational
C1221	AK442565	not applicable			-
C1233f	HX954431	not applicable			-
C1233r	HX954232	tudor domain-containing protein	2.88E-22	70.95%	F:metal ion binding; F:nucleic acid binding
C1272	AK442567	endothelial differentiation-related factor 1 homolog	6.32E-59	90.70%	P:central nervous system development; F:protein binding, bridging; P:positive regulation of transcription from RNA polymerase II promoter; F:methyl-CpG binding; F:transcription coactivator activity; P:open tracheal system development; C:cytoplasm; P:dendrite

* P: biological process, C: cellular component, F: molecular function

Table S4 continued

Sequence Name	AccessionNumber	Sequence Description	Min. eValue	Mean Similarity	Gene ontology terms*
cleavage-stage library					
C1273f	HX954434	transmembrane emp24 domain-containing	4.65E-62	72.60%	C:membrane
C1277	AK442568	ribosomal protein l39	4.98E-27	96.90%	C:ribosome; F:structural constituent of ribosome; P:translation
C1279	AK442569	not applicable			-
C1282	AK442570	not applicable			-
C1284	AK442571	nucleotide-binding protein 1	0	87.75%	F:metal ion binding; P:iron-sulfur cluster assembly; F:ATP binding; F:4 iron, 4 sulfur cluster binding; F:nucleoside-triphosphatase
C1289	AK442572	atp synthase f0 subunit 6	4.40E-23	81.10%	F:hydrogen ion transmembrane transporter activity; C:mitochondrial inner membrane; P:ATP synthesis coupled proton transport; C:proton-transporting ATP synthase complex,
C1291	AK442573	60s ribosomal protein l34-like	3.43E-66	92.90%	C:ribosome; F:structural constituent of ribosome; P:translation
C1294	AK442574	40s ribosomal protein s15-like	1.51E-52	98.50%	P:mitotic spindle elongation; P:translation; F:RNA binding; C:nuclear chromosome; C:nucleolus; F:structural constituent of ribosome; C:cytosolic small ribosomal subunit
C1307f	HX954435	not applicable			-
C1307r	HX954234	g2 mitotic-specific cyclin-b3-like	1.09E-35	61.00%	P:regulation of cell cycle
C1315f	HX954436	cyclin-dependent kinase 8-like	2.12E-112	90.45%	P:protein phosphorylation; F:RNA polymerase II carboxy-terminal domain kinase activity; P:regulation of transcription, DNA-dependent; P:cell division; F:ATP binding; F:cyclin-dependent protein serine/threonine kinase activity; P:G1/S transition of mitotic cell cycle; P:transcription, DNA-dependent; C:nucleus
C1315r	HX954235	cyclin-dependent kinase 8-like	2.37E-28	77.45%	P:protein phosphorylation; F:RNA polymerase II carboxy-terminal domain kinase activity; P:regulation of transcription, DNA-dependent; F:ATP binding; F:cyclin-dependent protein serine/threonine kinase activity; P:G1/S transition of mitotic cell cycle; P:transcription,
C1322f	HX954437	26s protease regulatory subunit 4	2.38E-41	75.10%	C:cytoplasm; P:protein catabolic process; F:ATP binding; F:nucleoside-triphosphatase activity
C1322r	HX954236	26s protease regulatory subunit 4	5.36E-27	91.50%	P:proteasomal ubiquitin-dependent protein catabolic process; P:mitotic spindle elongation; C:proteasome regulatory particle, base subcomplex; F:endopeptidase activity; F:ATP binding; F:microtubule-severing ATPase activity; P:cell proliferation; C:cytoplasm;
C1333	AK442577	not applicable			-
C1341	AK442578	not applicable			-
C1346f	HX954438	ribosomal protein l39	7.79E-28	96.60%	C:ribosome; F:structural constituent of ribosome; P:translation
C1352	AK442579	not applicable			-
C1361	AK442581	not applicable			-
C1370	AK442582	not applicable			-
C1378	AK442585	not applicable			-
C1381	AK442586	not applicable			-
C1393	AK442587	not applicable			-
C1404f	HX954439	not applicable			-

* P: biological process, C: cellular component, F: molecular function

Table S4 continued

Sequence Name	AccessionNumber	Sequence Description	Min. eValue	Mean Similarity	Gene ontology terms*
cleavage-stage library					
C1404r	HX954237	coiled-coil-helix-coiled-coil-helix domain-containing protein mitochondrial-	3.69E-10	82.75%	-
C1406	AK442588	not applicable			-
C1414	AK442589	not applicable			-
C1423	AK442591	not applicable			-
C1438	AK442594	not applicable			-
C1448	AK442595	probable rna helicase armi-like	6.17E-27	68.22%	F:helicase activity
C1508f	HX954440	ubiquitin-conjugating enzyme e2-17 kda-like	4.65E-39	99.60%	P:mitotic spindle organization; P:centrosome separation; P:protein ubiquitination; F:ubiquitin-protein ligase activity; C:nucleus; P:DNA repair
C1508r	HX954238	not applicable			-
C1518	AK442597	twinfilin-like isoform 2	0	87.55%	F:actin binding; C:intracellular
C1525	AK442598	not applicable			-
C1527	AK442599	acylphosphatase-1-like	7.94E-56	75.45%	F:acylphosphatase activity
C1536f	HX954441	not applicable			-
C1536r	HX954239	28s ribosomal protein mitochondrial-like	7.60E-108	73.55%	C:ribonucleoprotein complex
C1541	AK442600	46 kda fk506-binding nuclear protein	1.24E-17	68.85%	F:isomerase activity; P:protein peptidyl-prolyl isomerization
C1555	AK442601	not applicable			-
C1603f	HX954442	not applicable			-
C1624f	HX954444	atp-dependent rna helicase dhx8	2.01E-32	91.65%	F:RNA binding; F:ATP-dependent RNA helicase activity; C:nucleolus; P:mRNA splicing, via spliceosome; F:ATP binding; F:protein binding; C:catalytic step 2 spliceosome
C2103	AK442603	not applicable			-
C2133	AK442605	not applicable			-
primary morula-stage library					
M0003r	HX954240	protein phosphatase 1b-like	5.85E-107	82.35%	P:protein dephosphorylation; F:protein serine/threonine phosphatase activity; F:manganese ion binding; F:magnesium ion
M0007	AK442606	s-phase kinase-associated protein 1	3.25E-37	73.10%	P:cellular metabolic process
M0010	AK442607	not applicable			-
M0016	AK442608	transmembrane protein	1.34E-99	80.25%	C:integral to membrane
M0056	AK442609	cdgsh iron-sulfur domain-containing protein 2 homolog	7.96E-42	78.55%	F:metal ion binding; C:integral to membrane; C:endoplasmic reticulum membrane; F:2 iron, 2 sulfur cluster binding
M0059	AK442610	la protein homolog	7.83E-84	71.05%	F:nucleic acid binding; C:intracellular part
M0060	AK442611	not applicable			-
M0064	AK442613	protein kri1 homolog	3.81E-85	68.25%	-
M0065f	HX954446	cdk5 regulatory subunit-associated	1.83E-119	84.00%	P:transport; C:integral to membrane
M0065r	HX954241	cdk5 regulatory subunit-associated	7.87E-44	66.35%	C:integral to membrane; P:transport
M0071f	HX954447	low quality protein: nuclear valosin-	6.03E-08	55.00%	-
M0071r	HX954242	nuclear valosin-	2.79E-57	82.90%	F:nucleoside-triphosphatase activity; F:ATP
M0074f	HX954448	not applicable			-
M0074r	HX954243	not applicable			-

* P: biological process, C: cellular component, F: molecular function

Table S4 continued

Sequence Name	AccessionNumber	Sequence Description	Min. eValue	Mean Similarity	Gene ontology terms*
primary morula-stage library					
M0082	AK442616	not applicable			-
M0083f	HX954451	longitudinals lacking isoforms n o w x y-like isoform x66	5.68E-77	98.00%	-
M0083r	HX954245	zinc finger protein 282	1.04E-17	82.50%	F:metal ion binding; F:nucleic acid binding
M0084	AK442617	not applicable			-
M0087	AK442618	cytochrome c oxidase subunit mitochondrial-	3.87E-28	69.25%	F:cytochrome-c oxidase activity
M0090	AK442620	glyceraldehyde-3-phosphate dehydrogenase	0	93.70%	F:NAD binding; P:glucose metabolic process; F:glyceraldehyde-3-phosphate dehydrogenase (NAD+) (phosphorylating) activity; P:oxidation-reduction process; F:NADP binding
M0101f	HX954452	bleomycin hydrolase	1.98E-70	79.70%	F:cysteine-type endopeptidase activity;
M0101r	HX954246	bleomycin hydrolase	5.88E-106	80.45%	F:cysteine-type endopeptidase activity;
M0172r	HX954248	not applicable			-
M0176f	HX954454	regulator of chromosome	9.86E-30	78.00%	-
M0176r	HX954249	not applicable			-
M0217f	HX954455	tubulin alpha-1b chain-like	5.62E-101	98.65%	P:protein polymerization; C:cytoplasmic microtubule; P:GTP catabolic process; P:microtubule-based process; F:GTPase activity; F:structural constituent of cytoskeleton; F:GTP
M0228f	HX954456	not applicable			-
M0228r	HX954250	not applicable			-
M0232f	HX954458	protein aatf-like	5.32E-16	66.67%	-
M0232r	HX954251	protein aatf-like	4.30E-68	83.85%	C:nucleus
M0238f	HX954459	not applicable			-
M0238r	HX954252	not applicable			-
M0240	AK442623	atp synthase lipid-binding mitochondrial	4.73E-32	83.65%	F:hydrogen-exporting ATPase activity, phosphorylative mechanism; P:ATP hydrolysis coupled proton transport; C:integral to membrane; P:ATP synthesis coupled proton transport; F:lipid binding; C:mitochondrial proton-transporting ATP synthase complex,
M0241	AK442624	histone h3	4.94E-42	68.95%	F:binding; C:chromosome
M0243f	HX954460	protein phosphatase 1b-like	9.32E-09	73.20%	F:phosphoprotein phosphatase activity; F:metal ion binding
M0243r	HX954253	protein phosphatase 1b-like	1.78E-55	76.65%	P:protein dephosphorylation; F:protein serine/threonine phosphatase activity; F:manganese ion binding; F:magnesium ion
M0244f	HX954461	eukaryotic translation initiation factor 3 subunit l-like	1.21E-16	59.25%	C:cytoplasmic part; P:translation; C:macromolecular complex
M0244r	HX954254	eukaryotic translation initiation factor 3 subunit l-like	2.92E-116	83.15%	F:translation initiation factor activity; P:formation of translation preinitiation complex; C:eukaryotic 43S preinitiation complex; C:eukaryotic translation initiation factor 3 complex; P:neuron projection morphogenesis; C:eukaryotic 48S preinitiation complex;

* P: biological process, C: cellular component, F: molecular function

Table S4 continued

Sequence Name	AccessionNumber	Sequence Description	Min. eValue	Mean Similarity	Gene ontology terms*
primary morula-stage library					
M0245	AK442625	ras-related protein rab-11a-like	7.02E-154	96.45%	F:GTP binding; P:germ-line stem cell division; P:rhabdomere development; P:Golgi vesicle fusion to target membrane; C:trans-Golgi network transport vesicle; P:oocyte microtubule cytoskeleton polarization; P:actin cytoskeleton reorganization; C:recycling endosome; P:actomyosin contractile ring contraction; P:protein transport; P:cortical actin cytoskeleton organization; P:regulation of trichome morphogenesis; P:spermatocyte division; P:cellularization; C:fusome; P:regulation of pole plasm oskar mRNA localization; P:sensory organ precursor cell fate determination; P:regulation of synapse organization; P:dorsal closure, amnioserosa morphology change; C:terminal bouton; C:lipid particle; C:perinuclear region of cytoplasm; C:cleavage furrow; P:small GTPase mediated signal transduction; P:endocytosis; F:protein binding; C:spindle envelope; P:ovarian fusome organization; C:nuclear envelope; P:border follicle cell migration; P:chaeta development; P:dorsal closure, elongation of leading edge cells; P:antimicrobial humoral response; F:GTPase activity
M0284f	HX954462	polyubiquitin-b-like isoform 1	6.06E-114	99.20%	-
M0284r	HX954255	polyubiquitin-a- partial	5.14E-82	94.50%	P:female meiosis I; C:nucleoplasm; P:toll-like receptor 4 signaling pathway; P:translesion synthesis; P:seminiferous tubule development; P:energy homeostasis; P:toll-like receptor 2 signaling pathway; P:male meiosis I; P:female gonad development; P:hypothalamus gonadotrophin-releasing hormone neuron development; P:toll-like receptor 7 signaling pathway; C:cytoplasm; P:toll-like receptor 15 signaling pathway; P:toll-like receptor 5 signaling pathway; P:activation of MAPK activity involved in innate immune response; P:activation of NF-kappaB-inducing kinase activity; P:toll-like receptor 3 signaling pathway; P:fat pad development; P:ubiquitin homeostasis
M0285	AK442626	not applicable			-
M0286	AK442627	PREDICTED: hypothetical protein LOC100120938	1.02E-90	58.28%	-
M0290	AK442628	protein dr1-like	6.48E-88	89.15%	P:regulation of transcription from RNA polymerase II promoter; F:sequence-specific DNA binding; F:transcription cofactor activity; C:nucleus; F:protein heterodimerization activity
M0312f	HX954464	protein fam40a-like	1.57E-16	83.05%	-
M0312r	HX954257	not applicable			-

* P: biological process, C: cellular component, F: molecular function

Table S4 continued

Sequence Name	AccessionNumber	Sequence Description	Min. eValue	Mean Similarity	Gene ontology terms*
primary morula-stage library					
M0320f	HX954466	eukaryotic translation initiation factor 4e-binding protein 2	1.16E-39	73.50%	F:binding
M0333f	HX954467	not applicable			-
M0333r	HX954259	low quality protein: neogenin-like	2.23E-85	83.15%	C:integral to membrane
M0334f	HX954468	myosin heavy non-muscle	1.68E-47	91.10%	F:actin binding; C:myosin complex; F:ATP binding; F:motor activity
M0334r	HX954260	myosin heavy non-	5.53E-67	85.10%	C:myosin complex; F:ATP binding; F:motor
M0345f	HX954469	mrna cap guanine-n7 methyltransferase	1.57E-34	82.45%	P:7-methylguanosine mRNA capping; F:mRNA (guanine-N7-)-methyltransferase activity; F:RNA binding; P:RNA (guanine-N7)-
M0345r	HX954261	mrna cap guanine-n7 methyltransferase-like	2.39E-23	70.95%	P:mRNA processing; P:methylation; P:7-methylguanosine RNA capping; F:methyltransferase activity
M0350f	HX954470	heat shock protein 70 partial	1.29E-75	94.05%	F:ATP binding; P:response to stress
M0350r	HX954262	not applicable			-
M0352f	HX954471	cop9 signalosome complex subunit 4-like	1.62E-53	86.20%	-
M0352r	HX954263	cop9 signalosome complex subunit 4-like	1.40E-102	88.55%	C:cytoplasm; C:COP9 signalosome
M0361f	HX954472	not applicable			-
M0361r	HX954264	not applicable			-
M0362f	HX954473	myeloid leukemia factor-like	1.87E-25	78.75%	-
M0362r	HX954265	not applicable			-
M0383f	HX954474	casein kinase i isoform alpha	4.60E-55	94.50%	C:cytosol; P:protein phosphorylation; P:Wnt receptor signaling pathway; F:protein serine/threonine kinase activity; C:nuclear speck; C:centrosome; P:cell division; F:ATP binding; F:protein binding; P:mitosis; C:condensed chromosome kinetochore; C:mRNA cleavage and polyadenylation specificity factor complex
M0383r	HX954266	casein kinase i isoform alpha-like	1.07E-56	95.40%	C:cytosol; F:peptide binding; P:protein phosphorylation; P:Wnt receptor signaling pathway; F:protein serine/threonine kinase activity; F:glycoprotein binding; P:cell morphogenesis; C:nuclear speck; C:centrosome; P:cell division; F:ATP binding; F:magnesium ion binding; P:mitosis; F:phosphoprotein binding; C:ribonucleoprotein complex; C:condensed chromosome kinetochore; C:mRNA cleavage and polyadenylation
M0408f	HX954475	not applicable			-
M0408r	HX954267	serine arginine repetitive matrix	3.17E-28	85.75%	-
M0425f	HX954477	u3 small nucleolar rna-associated protein 6 homolog	6.18E-41	72.35%	P:RNA processing; C:intracellular
M0425r	HX954269	u3 small nucleolar rna-associated protein 6 homolog	1.77E-19	61.30%	P:RNA processing; C:intracellular
M0473f	HX954478	out at first	2.53E-83	79.85%	P:multicellular organismal development

* P: biological process, C: cellular component, F: molecular function

Table S4 continued

Sequence Name	AccessionNumber	Sequence Description	Min. eValue	Mean Similarity	Gene ontology terms*
primary morula-stage library					
M0473r	HX954270	not applicable			-
M0476f	HX954479	upf0668 protein c10orf76 homolog	5.81E-40	62.45%	-
M0476r	HX954271	upf0668 protein c10orf76 homolog	6.98E-39	79.05%	-
M0572f	HX954480	not applicable			-
M0572r	HX954272	not applicable			-
M0573f	HX954481	lamin-b receptor	6.23E-28	55.67%	C:membrane
M0573r	HX954273	not applicable			-
M0621	AK442629	cation transport regulator-like protein 2-	3.72E-108	68.85%	-
M0631	AK442630	not applicable			-
M0636f	HX954484	heat shock protein 70	7.93E-148	96.30%	F:ATP binding; P:response to stress
M0636r	HX954276	heat shock cognate 70 protein	1.72E-10	89.50%	F:ATP binding; P:response to stress
M0664f	HX954485	not applicable			-
M0664r	HX954277	low quality protein: neogenin-like	7.60E-54	85.25%	C:integral to membrane
M0697	AK442631	not applicable			-
M0719f	HX954486	not applicable			-
M0719r	HX954278	not applicable			-
M0877	AK442632	eukaryotic translation initiation factor 4e-binding protein 2	4.22E-38	73.50%	F:binding
M0914f	HX954489	dna replication factor cdt1-like	5.35E-11	53.00%	-
M0914r	HX954282	not applicable			-
M0954f	HX954492	not applicable			-
M0954r	HX954284	low quality protein: neogenin-like	3.83E-80	80.15%	C:integral to membrane
M0990	AK442633	dnaj homolog subfamily c member	2.31E-25	85.00%	-
M1002	AK442634	protein kinase c	4.82E-76	90.20%	F:kinase activity; P:phosphorylation
M1028f	HX954494	not applicable			-
M1028r	HX954285	not applicable			-
M1338f	HX954495	heat shock protein 70	3.26E-141	96.30%	F:ATP binding; P:response to stress
M1338r	HX954286	not applicable			-
M1356f	HX954497	not applicable			-
M1356r	HX954288	not applicable			-
M1368f	HX954498	pleckstrin homology domain-containing family f member 2-like	7.23E-09	45.60%	F:phospholipid binding; F:metal ion binding
M1368r	HX954289	not applicable			-
M1455f	HX954500	60 kda heat shock mitochondrial-like	5.43E-17	82.35%	C:cytoplasm; P:response to stress; F:solute:hydrogen antiporter activity; P:transmembrane transport; F:ATP binding; P:cation transport; P:protein refolding;
M1455r	HX954291	60 kda heat shock mitochondrial-like	9.11E-31	89.90%	C:cytoplasm; P:response to stress; F:solute:hydrogen antiporter activity; P:transmembrane transport; F:ATP binding; P:cation transport; P:protein refolding;
M1500r	HX954292	not applicable			-

* P: biological process, C: cellular component, F: molecular function

Table S4 continued

Sequence Name	AccessionNumber	Sequence Description	Min. eValue	Mean Similarity	Gene ontology terms*
primary morula-stage library					
M1827r	HX954294	dna damage-binding protein 1-like	3.72E-47	83.80%	P:proteasomal ubiquitin-dependent protein catabolic process; P:cell cycle checkpoint; P:Wnt receptor signaling pathway; F:damaged DNA binding; P:DNA repair; C:Cul4A-RING ubiquitin ligase complex; C:Cul4B-RING ubiquitin ligase complex; P:protein ubiquitination involved in ubiquitin-dependent
M1839r	HX954295	not applicable			-
M1849f	HX954502	heat shock cognate protein 70	7.53E-120	96.60%	P:axon guidance; P:transcription, DNA-dependent; P:neurotransmitter secretion; P:mRNA processing; C:melanosome; P:negative regulation of transcription, DNA-dependent; C:nucleolus; C:cytosol; P:membrane organization; C:spliceosomal complex; C:cell surface; C:clathrin-sculpted gamma-aminobutyric acid transport vesicle membrane; P:RNA splicing; P:regulation of cell cycle; F:unfolded protein binding; P:chaperone mediated protein folding requiring cofactor; C:extracellular vesicular exosome; C:Prp19 complex; P:modulation by virus of host morphology or physiology
M1849r	HX954296	heat shock protein 70	7.56E-22	81.65%	F:ATP binding; P:response to stress
M1860f	HX954503	golgi phosphoprotein 3-	5.10E-15	63.30%	-
M1860r	HX954297	not applicable			-
M1863f	HX954504	not applicable			-
M1863r	HX954298	arginine serine-rich coiled-coil protein 2-like isoform x1	1.24E-25	80.60%	-
M1895f	HX954506	structural maintenance of chromosomes protein 2-like	2.18E-16	90.70%	P:DNA recombination; P:DNA repair; P:mitotic chromosome condensation; F:ATP binding; P:sister chromatid cohesion; C:condensin
M1897f	HX954507	dna damage-binding protein 1-like	5.82E-67	91.55%	C:nucleus; F:nucleic acid binding
M1897r	HX954300	dna damage-binding protein 1-like	1.58E-43	86.45%	P:proteasomal ubiquitin-dependent protein catabolic process; P:cell cycle checkpoint; P:Wnt receptor signaling pathway; F:damaged DNA binding; P:DNA repair; C:Cul4A-RING ubiquitin ligase complex; C:Cul4B-RING ubiquitin ligase complex; P:protein ubiquitination involved in ubiquitin-dependent
M1900f	HX954508	truncated actin-4	1.77E-71	99.00%	C:cytoskeleton; C:cytoplasm; F:ATP binding
M1900r	HX954301	not applicable			-
M1913f	HX954509	trna (cytosine -c)-methyltransferase-like	7.44E-97	77.00%	F:RNA binding; F:tRNA (cytosine-5-)-methyltransferase activity; P:methylation
M1913r	HX954302	trna (cytosine -c)-methyltransferase-like	2.97E-66	67.05%	F:transferase activity
M1938f	HX954511	cation transport regulator-like protein 2-	4.23E-09	85.00%	-
M1938r	HX954304	cation transport regulator-like protein 2-	2.96E-13	84.00%	-
M1958f	HX954514	an1-type zinc finger protein 1-like	3.67E-39	75.40%	F:zinc ion binding

* P: biological process, C: cellular component, F: molecular function

Table S4 continued

Sequence Name	AccessionNumber	Sequence Description	Min. eValue	Mean Similarity	Gene ontology terms*
primary morula-stage library					
M1958r	HX954307	not applicable			-
M1962f	HX954515	serine hydroxymethyltransferase	4.33E-19	87.60%	F:pyridoxal phosphate binding; P:L-serine metabolic process; P:tetrahydrofolate interconversion; P:methylation; F:methyltransferase activity; F:glycine hydroxymethyltransferase activity; P:glycine
M1962r	HX954308	not applicable			-
M1986f	HX954516	not applicable			-
M1986r	HX954309	not applicable			-
M1994f	HX954517	negative elongation factor d	2.43E-69	79.95%	F:translation elongation factor activity; P:negative regulation of transcription, DNA-dependent; C:nucleus; P:translational elongation
M1994r	HX954310	negative elongation factor d	2.77E-54	91.05%	F:translation elongation factor activity; P:negative regulation of transcription, DNA-dependent; C:nucleus; P:translational elongation
M2006f	HX954518	tubulin alpha-1b chain-like	9.91E-97	98.70%	P:cellular response to interleukin-4; P:protein polymerization; C:cytoplasmic microtubule; P:GTP catabolic process; P:microtubule-based process; F:GTPase activity; F:structural constituent of cytoskeleton; F:GTP binding
M2006r	HX954311	tubulin alpha-1c chain-like	5.01E-101	96.75%	C:microtubule; P:protein polymerization; P:GTP catabolic process; P:microtubule-based process; F:GTPase activity; F:structural constituent of cytoskeleton; C:cytoplasm; F:GTP binding
M2009	AK442635	PREDICTED: hypothetical protein LOC100120938	4.23E-67	69.38%	-
M2015r	HX954312	negative elongation factor d	1.05E-49	88.95%	F:translation elongation factor activity; P:negative regulation of transcription, DNA-dependent; C:nucleus; P:translational elongation
M2022f	HX954519	mannose-1-phosphate guanyltransferase beta-like	1.27E-20	93.10%	F:mannose-1-phosphate guanylyltransferase activity; P:biosynthetic process
M2022r	HX954313	mannose-1-phosphate guanyltransferase beta-like	4.37E-63	88.10%	P:GDP-mannose biosynthetic process; F:GTP binding; F:mannose-1-phosphate guanylyltransferase activity
M2027f	HX954520	mannose-1-phosphate guanyltransferase beta-like	3.39E-25	91.55%	F:mannose-1-phosphate guanylyltransferase activity; P:biosynthetic process
M2027r	HX954314	mannose-1-phosphate guanyltransferase beta-like	1.13E-63	88.10%	P:GDP-mannose biosynthetic process; F:GTP binding; F:mannose-1-phosphate guanylyltransferase activity
M2037f	HX954521	carrier protein 2-like	7.57E-66	93.00%	C:mitochondrial inner membrane; P:transmembrane transport; C:integral to membrane; F:transporter activity
M2037r	HX954315	adp atp translocase	2.18E-42	93.85%	C:mitochondrial inner membrane; P:transmembrane transport; C:integral to membrane; F:transporter activity
M2044f	HX954522	not applicable			-
M2044r	HX954316	guanine nucleotide-binding protein g g g subunit beta-1-like	4.49E-86	97.45%	F:histone acetyltransferase activity; P:histone acetylation
M2060f	HX954523	serine arginine repetitive matrix	6.74E-24	67.17%	-

* P: biological process, C: cellular component, F: molecular function

Table S4 continued

Sequence Name	AccessionNumber	Sequence Description	Min. eValue	Mean Similarity	Gene ontology terms*
primary morula-stage library					
M2060r	HX954317	ring and phd-finger domain-containing protein kiaa1542	9.66E-21	84.30%	F:zinc ion binding
M2063f	HX954525	dual specificity mitogen-activated protein kinase kinase	3.62E-70	80.40%	P:protein phosphorylation; F:ATP binding; F:protein serine/threonine kinase activity
M2063r	HX954319	map kinse-erk kinase	8.25E-38	92.05%	P:protein phosphorylation; F:ATP binding; F:protein serine/threonine kinase activity
M2083f	HX954526	pyruvate kinase	4.82E-59	83.90%	F:pyruvate kinase activity; P:phosphorylation; F:potassium ion binding; F:magnesium ion binding; P:glycolysis
M2083r	HX954320	not applicable			-
M2089f	HX954527	not applicable			-
M2089r	HX954321	protein phosphatase 1b-like	1.11E-104	82.35%	P:protein dephosphorylation; F:protein serine/threonine phosphatase activity; F:manganese ion binding; F:magnesium ion
M2099f	HX954528	PREDICTED: hypothetical protein LOC100678553	5.21E-07	52.00%	-
M2099r	HX954322	PREDICTED: hypothetical protein LOC100678553	5.28E-12	57.33%	-
M2103f	HX954529	not applicable			-
M2103r	HX954323	not applicable			-
M2111r	HX954324	not applicable			-
M2117	AK442637	not applicable			-
M2601f	HX954530	dcn1-like protein 1-like	8.14E-72	81.30%	F:molecular_function; P:biological_process
M2601r	HX954325	not applicable			-
M2622f	HX954531	not applicable			-
M2622r	HX954326	not applicable			-
M2629f	HX954532	not applicable			-
M2629r	HX954327	not applicable			-
M2640	AK442638	atp synthase lipid-binding mitochondrial	7.57E-38	88.35%	F:hydrogen-exporting ATPase activity, phosphorylative mechanism; P:ATP hydrolysis coupled proton transport; C:integral to membrane; P:ATP synthesis coupled proton transport; F:lipid binding; C:mitochondrial proton-transporting ATP synthase complex,
M2654f	HX954533	soluble nsf attachment	2.90E-44	88.35%	P:intracellular protein transport
M2654r	HX954328	soluble nsf attachment protein	1.19E-26	88.10%	P:intracellular protein transport
M3121	AK442640	not applicable			-
M3142	AK442643	eukaryotic translation initiation factor 6-like	1.32E-75	93.60%	C:cytoplasm; C:nucleolus; P:ribosomal large subunit biogenesis; P:translational initiation; P:mature ribosome assembly; F:translation initiation factor activity; F:ribosomal large subunit binding; F:ribosome binding
M3156	AK442644	not applicable			-
M4014f	HX954535	not applicable			-
M4014r	HX954330	not applicable			-
M4016	AK442646	not applicable			-
M4023f	HX954536	protein max-like	9.06E-24	84.80%	F:sequence-specific DNA binding transcription factor activity; F:protein dimerization activity; C:nucleus; P:regulation of transcription, DNA-

* P: biological process, C: cellular component, F: molecular function

Table S4 continued

Sequence Name	AccessionNumber	Sequence Description	Min. eValue	Mean Similarity	Gene ontology terms*
primary morula-stage library					
M4042	AK442648	not applicable			-
M4045f	HX954538	peptidyl-prolyl cis-trans isomerase-like	3.12E-45	90.55%	P:protein folding; P:protein peptidyl-prolyl isomerization; F:peptidyl-prolyl cis-trans isomerase activity
M4045r	HX954331	peptidyl-prolyl cis-trans isomerase-like	1.95E-92	92.30%	P:protein folding; P:protein peptidyl-prolyl isomerization; F:peptide binding; C:cytoplasm; F:peptidyl-prolyl cis-trans isomerase activity
M4050f	HX954539	not applicable			-
M4050r	HX954332	not applicable			-
M4075f	HX954540	heat shock protein 70	2.48E-120	95.20%	F:ATP binding; P:response to stress
M4075r	HX954333	not applicable			-
M4077	AK442649	not applicable			-
M4080	AK442650	atp synthase f0 subunit	4.83E-34	74.50%	C:mitochondrion; C:membrane
M4097f	HX954541	not applicable			-
M4097r	HX954334	not applicable			-
M4115r	HX954335	not applicable			-
M4176	AK442651	ribosomal protein s21	2.72E-47	87.40%	P:endonucleolytic cleavage to generate mature 3'-end of SSU-rRNA from (SSU-rRNA, 5.8S rRNA, LSU-rRNA); P:regulation of translation; F:structural constituent of ribosome; P:translational elongation; C:cytosolic small ribosomal subunit; F:ribosome binding; P:lymph gland development; P:regulation of cell proliferation; P:endonucleolytic cleavage in ITS1 to separate SSU-rRNA from 5.8S rRNA and LSU-rRNA from tricistronic rRNA
M4200f	HX954543	transcription initiation factor tfiid subunit 3	9.79E-23	89.55%	F:protein heterodimerization activity; F:zinc ion binding; P:translational initiation; F:translation initiation factor activity
M4200r	HX954337	PREDICTED: hypothetical protein LOC100679728	3.06E-13	51.00%	-
M4230f	HX954544	heat shock protein 70	5.56E-61	89.05%	C:cell surface; P:negative regulation of transcription, DNA-dependent; C:extracellular vesicular exosome; P:post-Golgi vesicle-mediated transport; P:modulation by virus of host morphology or physiology; C:spliceosomal complex; F:unfolded protein binding; C:clathrin-sculpted gamma-aminobutyric acid transport vesicle membrane; C:CatSper complex; P:axon guidance; P:synaptonemal complex disassembly; C:cytosol; C:Prp19 complex; F:glycolipid binding; P:transcription, DNA-dependent; P:chaperone mediated protein folding requiring cofactor; P:RNA splicing; P:male meiosis I; P:response to unfolded protein; F:ATPase activity, coupled; P:response to heat; P:spermatid development; C:mitochondrion; P:positive regulation of cyclin-dependent protein serine/threonine kinase activity involved in G2/M transition of mitotic cell cycle; F:ATP binding
M4230r	HX954338	not applicable			-

* P: biological process, C: cellular component, F: molecular function

Table S4 continued

Sequence Name	AccessionNumber	Sequence Description	Min. eValue	Mean Similarity	Gene ontology terms*
primary morula-stage library					
M4238f	HX954545	protein phosphatase inhibitor 2-like	2.31E-50	78.00%	P:regulation of phosphoprotein phosphatase activity; P:regulation of signal transduction; F:protein phosphatase inhibitor activity
M4238r	HX954339	protein phosphatase inhibitor 2-like	3.67E-50	77.80%	P:regulation of phosphoprotein phosphatase activity; P:regulation of signal transduction; F:protein phosphatase inhibitor activity
M4267f	HX954546	elongation factor 1 alpha	2.74E-127	97.00%	F:translation elongation factor activity; C:eukaryotic translation elongation factor 1 complex; P:neurogenesis; P:translational elongation; P:GTP catabolic process; F:GTPase activity; C:lipid particle; F:GTP binding
M4267r	HX954340	not applicable			-
M4303r	HX954342	not applicable			-
M4338r	HX954343	not applicable			-
M4351f	HX954548	tudor and kh domain-containing	6.76E-16	58.33%	-
M4351r	HX954344	not applicable			-
M4358f	HX954549	zinc transporter zip3-like	1.09E-65	62.35%	C:membrane; P:transmembrane transport; P:metal ion transport; F:metal ion transmembrane transporter activity
M4358r	HX954345	not applicable			-
M4372f	HX954550	traf-interacting protein	1.88E-48	69.45%	F:metal ion binding; F:zinc ion binding
M4372r	HX954346	not applicable			-
M4385f	HX954551	not applicable			-
M4385r	HX954347	not applicable			-
M4405f	HX954553	not applicable			-
M4405r	HX954348	not applicable			-
M4417	AK442653	endothelial differentiation-related factor 1 homolog	1.09E-75	89.40%	P:central nervous system development; F:protein binding, bridging; P:positive regulation of transcription from RNA polymerase II promoter; F:methyl-CpG binding; F:transcription coactivator activity; P:open tracheal system development; C:cytoplasm; P:dendrite
M4430f	HX954555	t-complex protein 1 subunit gamma	2.09E-76	92.70%	C:microtubule associated complex; P:terminal branching, open tracheal system; P:trachea morphogenesis; P:protein folding; F:ATPase activity, coupled; C:chaperonin-containing T-complex; P:mitotic spindle organization; F:ATP binding; F:unfolded protein binding
M4430r	HX954350	t-complex protein 1 subunit gamma	2.14E-61	88.20%	P:'de novo' posttranslational protein folding; C:microtubule; C:chaperonin-containing T-complex; P:binding of sperm to zona pellucida; F:ATP binding; F:unfolded protein binding; C:plasma membrane; C:zona pellucida receptor
M4453f	HX954556	ribose-phosphate pyrophosphokinase 1-like isoform 1	3.64E-135	88.70%	P:ribonucleoside monophosphate biosynthetic process; P:nucleotide biosynthetic process; P:imaginal disc-derived wing morphogenesis; P:nucleoside metabolic process; F:magnesium ion binding; P:phosphorylation; F:kinase activity; F:ribose phosphate diphosphokinase
M4453r	HX954351	not applicable			-
M4472f	HX954557	not applicable			-
M4472r	HX954352	not applicable			-

* P: biological process, C: cellular component, F: molecular function

Table S4 continued

Sequence Name	AccessionNumber	Sequence Description	Min. eValue	Mean Similarity	Gene ontology terms*
primary morula-stage library					
M4517f	HX954558	polyadenylate-binding protein 1-like isoform 1	5.14E-90	98.50%	F:nucleotide binding; F:RNA binding
M4517r	HX954353	polyadenylate-binding protein 1-like isoform 1	2.14E-49	90.95%	P:translation; C:cytoplasmic stress granule; P:RNA processing; F:poly(U) RNA binding; P:RNA catabolic process; F:poly(C) RNA binding; F:poly(A) RNA binding; F:protein binding; F:nucleotide binding; C:nucleus;
M4521f	HX954559	fact complex subunit ssrp1	3.01E-74	84.75%	C:nucleus; F:DNA binding
M4591f	HX954560	not applicable			-
M4591r	HX954354	bromo adjacent-like proteiny domain-containing 1 protein	1.21E-70	78.20%	F:DNA binding
M4607f	HX954561	not applicable			-
M4607r	HX954355	not applicable			-
M4610	AK442654	28s ribosomal protein mitochondrial	2.48E-78	64.15%	C:ribosome
M4642f	HX954562	e3 ubiquitin-protein ligase uhrf1-like	1.69E-45	60.90%	F:binding
M4642r	HX954356	e3 ubiquitin-protein ligase uhrf1-like	2.25E-77	80.20%	P:positive regulation of cellular protein metabolic process; P:protein autoubiquitination; P:negative regulation of transcription from RNA polymerase II promoter; P:histone monoubiquitination; F:zinc ion binding; P:protein ubiquitination involved in ubiquitin-dependent protein catabolic process; P:cell proliferation; F:nucleosomal histone binding; C:euchromatin; P:maintenance of DNA methylation; F:ubiquitin-protein ligase activity; F:identical protein binding; P:positive regulation of DNA topoisomerase (ATP-hydrolyzing) activity; C:heterochromatin; C:nuclear matrix; F:sequence-specific DNA binding transcription factor activity; C:nuclear chromatin; F:methyl-CpG binding; P:positive regulation of transcription from RNA polymerase II promoter; F:core promoter proximal region sequence-specific DNA binding; F:hemi-methylated DNA-
M4655f	HX954563	bag family molecular chaperone regulator 2-	2.58E-46	78.35%	F:kinase activity; P:phosphorylation
M4655r	HX954357	not applicable			-
M4656f	HX954564	low quality protein: regulator of chromosome	1.75E-14	54.80%	-
M4656r	HX954358	not applicable			-
M4737	AK442656	rpii140-upstream gene	2.28E-35	65.30%	P:multicellular organismal development
M4761f	HX954568	leptin receptor overlapping transcript-	1.62E-69	84.60%	-
M4761r	HX954361	not applicable			-
M4772	AK442658	atp synthase subunit 6	6.94E-38	75.25%	C:mitochondrion; C:membrane
M4778f	HX954570	rna polymerase ii subunit a c-terminal domain phosphatase	1.24E-45	85.80%	F:phosphoprotein phosphatase activity; P:mRNA processing; C:nucleus

* P: biological process, C: cellular component, F: molecular function

Table S4 continued

Sequence Name	AccessionNumber	Sequence Description	Min. eValue	Mean Similarity	Gene ontology terms*
primary morula-stage library					
M4778r	HX954363	rna polymerase ii subunit a c-terminal domain phosphatase	3.40E-67	85.10%	F:phosphoprotein phosphatase activity; P:mRNA processing; C:nucleus
M4786f	HX954571	not applicable			-
M4786r	HX954364	not applicable			-
M4789	AK442659	methyltransferase-like protein 2-a-like	0	72.55%	F:transferase activity
M4806f	HX954572	not applicable			-
M4806r	HX954365	ring finger protein 10-	2.92E-26	57.25%	F:metal ion binding; F:zinc ion binding
M4817	AK442661	not applicable			-
M4818	AK442662	glutaredoxin 3-like	3.71E-101	76.50%	P:regulation of biological quality; C:cytoplasmic part; P:regulation of system process
M4843f	HX954573	not applicable			-
M4843r	HX954366	histone-lysine n-methyltransferase nsd2	1.02E-35	68.46%	F:transferase activity
M4851f	HX954574	not applicable			-
M4851r	HX954367	not applicable			-
M4863f	HX954575	not applicable			-
M4863r	HX954368	cdc42-interacting protein 4-like	2.27E-110	90.30%	-
M4904f	HX954577	tubulin alpha-1c chain	1.12E-100	98.80%	P:protein polymerization; C:cytoplasmic microtubule; P:GTP catabolic process; P:microtubule-based process; F:GTPase activity; F:structural constituent of cytoskeleton; F:GTP
M4913	AK442664	high mobility group protein dsp1	2.71E-107	90.10%	P:negative regulation of antimicrobial humoral response; P:positive regulation of DNA binding; P:negative regulation of RNA polymerase II transcriptional preinitiation complex assembly; F:TBP-class protein binding; F:metal ion binding; P:imaginal disc-derived leg morphogenesis; F:DNA binding, bending; F:sequence-specific DNA binding; C:polytene chromosome; F:sequence-specific DNA binding transcription factor activity; P:segment
M4915	AK442665	cyclin-dependent kinases regulatory subunit 1-like	4.00E-42	88.45%	F:kinase activity; P:cell cycle; F:cyclin-dependent protein serine/threonine kinase regulator activity; P:phosphorylation
M4932	AK442666	not applicable			-
M4943	AK442670	not applicable			-
M4945f	HX954578	polyadenylate-binding protein 1-like isoform 1	7.46E-96	84.35%	F:nucleotide binding; F:RNA binding
M4946	AK442671	not applicable			-
M4950f	HX954579	abc transporter f family member 4-like isoform	1.07E-26	73.29%	F:DNA binding
M4950r	HX954370	not applicable			-
M4954	AK442673	replication protein a 14 kda subunit	1.79E-61	61.90%	C:cytoplasm
M4961f	HX954581	not applicable			-
M4961r	HX954372	not applicable			-
M4962	AK442674	60s ribosomal protein l35-like	3.40E-62	81.70%	C:ribosome; F:structural constituent of ribosome; P:translation
M4975f	HX954582	not applicable			-
M4975r	HX954373	integrase core domain protein	2.82E-47	51.55%	F:catalytic activity

* P: biological process, C: cellular component, F: molecular function

Table S4 continued

Sequence Name	AccessionNumber	Sequence Description	Min. eValue	Mean Similarity	Gene ontology terms*
primary morula-stage library					
M4976f	HX954583	fructose-bisphosphate aldolase	4.86E-101	89.10%	P:glycolysis; F:fructose-bisphosphate aldolase activity
M4976r	HX954374	fructose-bisphosphate aldolase	1.18E-31	81.95%	P:single-organism developmental process; P:nervous system development; P:glycolysis; P:anatomical structure morphogenesis; F:fructose-bisphosphate aldolase activity
M4988	AK442675	not applicable			-
M4995f	HX954585	lamin dm0-like	2.41E-94	84.95%	P:regulation of actin filament polymerization; C:lamin filament; C:spindle envelope; P:chromatin silencing; P:nuclear pore distribution; P:tendon development; P:chromatin organization; F:structural molecule activity; C:nuclear inner membrane; P:mitotic nuclear
M5005f	HX954586	not applicable			-
M5005r	HX954376	not applicable			-
M5010f	HX954587	protein max	7.64E-46	78.65%	F:sequence-specific DNA binding transcription factor activity; F:protein dimerization activity; C:nucleus; P:regulation of transcription, DNA-
M5017	AK442676	dna-directed rna polymerases and iii subunit rpabc5	4.97E-38	97.15%	P:transcription elongation from RNA polymerase III promoter; P:transcription initiation from RNA polymerase II promoter; P:7-methylguanosine mRNA capping; P:positive regulation of type I interferon production; F:DNA-directed RNA polymerase activity; F:zinc ion binding; F:protein kinase activity; P:transcription-coupled nucleotide-excision repair; P:termination of RNA polymerase III transcription; C:cytosol; P:protein phosphorylation; C:DNA-directed RNA polymerase II, core complex; F:DNA binding; P:positive regulation of viral transcription; P:viral process; P:mRNA splicing, via spliceosome; P:transcription elongation from
M5039f	HX954588	gmp synthase	9.25E-34	83.55%	F:GMP synthase (glutamine-hydrolyzing) activity; F:ATP binding; F:pyrophosphatase activity; P:GMP biosynthetic process
M5039r	HX954377	not applicable			-
M5060f	HX954589	not applicable			-
M5060r	HX954378	bromo adjacent-like proteiny domain-containing 1 protein	8.16E-75	81.75%	F:DNA binding
M5064f	HX954590	four and a half lim domains protein 2-like isoform 2	1.11E-116	93.90%	-
M5064r	HX954379	not applicable			-
M5088f	HX954591	heat shock 70 kda protein cognate 5-like	4.57E-75	81.95%	P:protein folding; P:response to stress; F:ATP binding; F:unfolded protein binding
M5088r	HX954380	heat shock 70 kda protein cognate 5-like	1.26E-118	93.90%	P:protein folding; P:response to stress; F:ATP binding; F:unfolded protein binding; F:2-alkenal reductase [NAD(P)] activity; P:oxidation-reduction process
M5097f	HX954592	histone acetyltransferase tip60-like	1.29E-43	82.05%	F:transferase activity, transferring acyl groups other than amino-acyl groups; C:nucleus; P:regulation of transcription, DNA-dependent

* P: biological process, C: cellular component, F: molecular function

Table S4 continued

Sequence Name	AccessionNumber	Sequence Description	Min. eValue	Mean Similarity	Gene ontology terms*
primary morula-stage library					
M5184f	HX954594	aldo-keto reductase	1.08E-135	78.10%	P:oxidation-reduction process; F:oxidoreductase activity
M5184r	HX954383	aldose reductase	3.09E-43	76.25%	P:daunorubicin metabolic process; C:cytosol; F:alditol:NADP+ 1-oxidoreductase activity; P:oxidation-reduction process; P:positive regulation of JAK-STAT cascade; P:sorbitol biosynthetic process; C:nucleolus; P:doxorubicin metabolic process; P:C21-steroid hormone biosynthetic process; C:extracellular space; F:electron carrier activity; F:glyceraldehyde oxidoreductase activity; P:stress-activated protein kinase signaling cascade; P:positive regulation of smooth muscle cell proliferation;
M5193f	HX954595	not applicable			-
M5193r	HX954384	not applicable			-
M5208	AK442678	28s ribosomal protein mitochondrial	8.74E-55	69.75%	C:ribosome
M5216f	HX954599	vacuolar atp synthase subunit e	3.04E-46	94.00%	P:ATP hydrolysis coupled proton transport; C:proton-transporting two-sector ATPase complex, catalytic domain; F:proton-transporting ATPase activity, rotational
M5216r	HX954388	vacuolar atp synthase subunit e	4.24E-91	81.65%	P:ATP hydrolysis coupled proton transport; C:proton-transporting two-sector ATPase complex, catalytic domain; F:proton-transporting ATPase activity, rotational
M5218	AK442679	60s ribosomal protein l34-like	3.96E-68	93.75%	C:ribosome; F:structural constituent of ribosome; P:translation
M5219f	HX954600	pre-mrna-processing factor 40-like protein a	1.43E-24	88.65%	P:RNA modification; F:catalytic activity; F:4 iron, 4 sulfur cluster binding
M5219r	HX954389	pre-mrna-processing factor 40 homolog a-	5.10E-113	88.35%	P:RNA modification; F:catalytic activity; F:4 iron, 4 sulfur cluster binding
M5246	AK442681	not applicable			-
M5267f	HX954601	PREDICTED: hypothetical protein LOC100114080	2.41E-17	56.50%	-
M5267r	HX954390	integrase core domain protein	1.95E-48	52.85%	F:RNA binding; F:nucleic acid binding; P:DNA integration; P:RNA-dependent DNA replication; F:RNA-directed DNA polymerase activity; F:nucleotidyltransferase activity; F:transferase activity; F:endonuclease activity; F:metal ion binding; F:zinc ion binding
M5304f	HX954604	tubulin alpha-1c chain	3.89E-97	98.75%	P:protein polymerization; C:cytoplasmic microtubule; P:GTP catabolic process; P:microtubule-based process; F:GTPase activity; F:structural constituent of cytoskeleton; F:GTP
M5320f	HX954605	not applicable			-
M5320r	HX954393	not applicable			-
M5333f	HX954606	heat shock 70 kda protein cognate 5-like	1.07E-46	76.45%	P:protein folding; P:response to stress; F:ATP binding; F:unfolded protein binding
M5333r	HX954394	heat shock 70 kda protein cognate 5-like	3.75E-93	91.75%	P:protein folding; P:response to stress; F:ATP binding; F:unfolded protein binding; F:2-alkenal reductase [NAD(P)] activity; P:oxidation-reduction process

* P: biological process, C: cellular component, F: molecular function

Table S4 continued

Sequence Name	AccessionNumber	Sequence Description	Min. eValue	Mean Similarity	Gene ontology terms*
primary morula-stage library					
M5358	AK442683	f-actin-capping protein subunit alpha-like	5.36E-21	92.50%	C:cytosol; C:cortical cytoskeleton; P:barbed-end actin filament capping; F:actin filament binding; P:innate immune response; P:protein complex assembly; P:actin cytoskeleton organization; C:WASH complex; P:cellular component movement; C:F-actin capping protein complex; C:extracellular region; C:membrane; P:blood
M5486r	HX954395	not applicable			-
M5506f	HX954608	u3 small nucleolar rna-associated protein 6 homolog	5.44E-34	68.15%	P:RNA processing; C:intracellular
M5506r	HX954397	u3 small nucleolar rna-associated protein 6 homolog	1.87E-36	61.45%	P:RNA processing; C:intracellular
M5564	AK442684	grpe protein mitochondrial-like	5.09E-111	80.80%	F:ribulose-phosphate 3-epimerase activity; P:protein folding; F:chaperone binding; C:mitochondrial matrix; F:adenyl-nucleotide exchange factor activity; F:protein
M5570f	HX954609	tudor and kh domain-containing	7.73E-07	63.00%	-
M5570r	HX954398	not applicable			-
M5573f	HX954610	serine threonine-protein kinase 3-like	2.27E-123	97.75%	P:protein phosphorylation; F:ATP binding; P:signal transduction; F:protein serine/threonine
M5573r	HX954399	serine threonine-protein kinase 3-like	2.35E-26	79.45%	P:protein phosphorylation; F:ATP binding; P:signal transduction; F:protein serine/threonine
M5662	AK442685	ras-related protein ral-a-like isoform x1	6.45E-125	86.10%	P:Ras protein signal transduction; P:regulation of cell morphogenesis; F:PDZ domain binding; P:negative regulation of JNK cascade; F:GTP binding; P:GTP catabolic process; P:R3/R4 cell fate commitment; P:dorsal closure; F:GTPase activity; C:plasma membrane; P:border follicle cell migration; P:innate immune response; P:regulation of Notch signaling pathway
M5721	AK442686	not applicable			-

* P: biological process, C: cellular component, F: molecular function
