# Supplementary material for: Gene Expression of Protein-Coding and Non-Coding RNAs Related to Polyembryogenesis in the Parasitic Wasp, Copidosoma floridanum
Source: PLoS One. 2014 Dec 3;9(12):e114372. doi: 10.1371/journal.pone.0114372 (PMC4255003; doi:10.1371/journal.pone.0114372)
Supplement: Table S5 — Classification of sequences derived from from C. floridanum embryos and duplicated with the formerly registered cDNA sequences derived from larvae. (PDF) [file pone.0114372.s009.pdf]

Table S5 Classification of sequences derived from from *C. floridanum* embryos and duplicated with the formerly registered cDNA sequences derived from larvae[*1].

Sequence Name	AccessionNumber	Sequence Description	Min. eValue	Mean Similarity	Gene ontology terms[*2]
primary morula-stage library					
M4728	AK442655	endocuticle structural glycoprotein bd-1-like	1.63E-39	70.95%	F:structural constituent of cuticle
M5281f	HX954602	sorting nexin-25-like	8.42E-42	61.30%	P:single-organism process
M5281r	HX954391	not applicable			-

*1 Accession: DV181803–DV182032 (Donnell and Strand, 2005)

*2 P: biological process, C: cellular component, F: molecular function
