# Supplementary material for: Gene Expression of Protein-Coding and Non-Coding RNAs Related to Polyembryogenesis in the Parasitic Wasp, Copidosoma floridanum
Source: PLoS One. 2014 Dec 3;9(12):e114372. doi: 10.1371/journal.pone.0114372 (PMC4255003; doi:10.1371/journal.pone.0114372)
Supplement: Table S6 — Classification of sequences derived from from C. floridanum embryos and duplicated with all of the cDNA collections used for comparison. (PDF) [file pone.0114372.s010.pdf]

Table S6 Classification of sequences derived from from *C. floridanum* embryos and duplicated with all of the cDNA collections[*1] used for comparison.

Sequence Name	AccessionNumber	Sequence Description	Min. eValue	Mean Similarity	Gene ontology terms[*2]
cleavage-stage library					
C0257	AK442480	pre-mrna-splicing factor spf27-like	6.31E-111	84.05%	P:mRNA processing
C0466	AK442495	60s ribosomal protein	1.14E-46	89.90%	C:ribosome; F:structural constituent of
primary morula-stage library					
M0239	AK442622	40s ribosomal protein s2-like	3.89E-132	92.15%	F:structural constituent of ribosome; F:RNA binding; C:small ribosomal subunit; P:translation
M3111	AK442639	60s ribosomal protein l36	6.09E-54	84.05%	C:ribosome; F:structural constituent of ribosome; P:translation
M4899f	HX954576	fatty acid binding	5.56E-53	75.95%	F:transporter activity; F:lipid binding;
M4899r	HX954369	fatty acid binding	2.28E-26	71.40%	F:lipid binding; P:transport; F:transporter
M4940	AK442669	ribosomal protein s20	4.85E-77	91.90%	F:structural constituent of ribosome; C:small ribosomal subunit; P:translation
M5242	AK442680	prohibitin	6.32E-80	63.45%	C:membrane

*1 Accession: JI831114–JI846296 (NCBI BioProject Accession: PRJNA65673) and Accession: DV181803–DV182032 (Donnell and Strand, 2005)

*2 P: biological process, C: cellular component, F: molecular function
