# Supplementary material for: Gene Expression of Protein-Coding and Non-Coding RNAs Related to Polyembryogenesis in the Parasitic Wasp, Copidosoma floridanum
Source: PLoS One. 2014 Dec 3;9(12):e114372. doi: 10.1371/journal.pone.0114372 (PMC4255003; doi:10.1371/journal.pone.0114372)
Supplement: Table S7 — Listing of clones in the clusters as a result of screening for frequency. (PDF) [file pone.0114372.s011.pdf]

Table S7 Listing of clones in the clusters as a result of screening for frequency.

Rank	Number of clones in	Sequence description	Clone name
1	58	not applicable (CflncRNA-1)	C0182, C0185, C0359, C0569, C0630, C0662, C0666, C0681, C0701, C0721, C0941, C1019, C1123, C1341, C1352, C1370, C1378, C1393, C1555, C1631, C2118, C2119, M0084, M0697, M4028, M4943, M5340, M5764
		not applicable (CflncRNA-2)	C0798, C0879, C1279, C1361, C1406, M4042, M4817
		not applicable (CflncRNA-3)	C0220, C0277, C0293, C0531, C0594, C0598, C0621, C0633, C0637, C0638, C0645, C0649, C0651, C0665, C0946, C1191, C1282, C1414, C1423, C2133, M2117, M5246
2	14	heat shock protein 70	C0451, C1438, M0350, M0636, M1338, M1500, M1839, M2111, M4075, M4115, M4230, M4303, M4338, M5486
3	7	not applicable	C0646, C0596, C0682, C0615, C0643, C0600, C0669,
4	5	tubulin alpha-1b chain-	C0170, C0217, M2006, M4904, M5304
5	4	polyadenylate-binding protein 1-like isoform 1	C0456, M4016, M4517, M4945
6	3	ferritin	C0324, C0809, C0844
6	3	atp synthase lipid-binding mitochondrial	C0430, M0240, M2640
6	3	atp synthase f0 subunit 6	C1289, M4772, M4080
6	3	regulator of chromosome condensation	C1182, M0176, M4656
6	3	PREDICTED: hypothetical protein	M0243, M2089, M0003
6	3	low quality protein: neogenin-like	M0333, M0664, M0954
6	3	not applicable	M1028, M1356, M1986
6	3	not applicable	C0586, C0587, C1317
7	2	replication protein a 14 kda subunit	C0329, M4954
7	2	PREDICTED: hypothetical protein	C0578, C0579
7	2	ribosomal protein l36e	C0466, M3111
7	2	zinc finger	C0544, C0640
7	2	46 kda fk506-binding nuclear protein	C0588, C1541
7	2	dna-directed rna polymerases and iii subunit	C0636, M5017
7	2	josephin-like	C0874, M5206
7	2	endothelial differentiation-related factor 1 homolog	C1272, M4417
7	2	ribosomal protein l39	C1277, C1346
7	2	ribosomal protein l34	C1291, M5218
7	2	bag family molecular chaperone regulator 2-like	C0908f, M4655
7	2	truncated actin-4	M1900, C2103

Table S7 continued

Rank	Number of clones in	Sequence description	Clone name
7	2	PREDICTED: hypothetical protein	M0074, M4014
7	2	PREDICTED: hypothetical protein	M0286, M2009
7	2	eukaryotic translation initiation factor 4e-binding protein 2	M0320, M0877
7	2	u3 small nucleolar rna-associated protein 6	M0425, M5506
7	2	pleckstrin homology domain-containing family f member 2-like	M0572, M1368
7	2	cation transport regulator-like protein 2-like	M0621, M1938
7	2	dna damage-binding	M1827, M1897
7	2	negative elongation factor	M1994, M2015
7	2	mannose-1-phosphate guanyltransferase beta-like	M2022, M2027
7	2	protein max-like	M4023, M5010
7	2	tudor and kh domain-containing	M4351, M5570
7	2	bromo adjacent-like proteiny domain-	M4591, M5060
7	2	28s ribosomal protein mitochondrial	M4610, M5208
7	2	odorant receptor 48	M4960, M5761
7	2	integrase core domain	M4975, M5267
7	2	heat shock 70 kda protein cognate 5-like	M5088, M5333
7	2	not applicable	C0380, M5320
7	2	not applicable	M0010, M0238
7	2	not applicable	C0052, C1507
7	2	not applicable	M0312, M0719
7	2	not applicable	M3156, M4988
7	2	not applicable	M4268, M4736
