# Supplementary material for: Gene Expression of Protein-Coding and Non-Coding RNAs Related to Polyembryogenesis in the Parasitic Wasp, Copidosoma floridanum
Source: PLoS One. 2014 Dec 3;9(12):e114372. doi: 10.1371/journal.pone.0114372 (PMC4255003; doi:10.1371/journal.pone.0114372)
Supplement: Supporting Information S1 — The run reports of RT-qPCR experiments for each target gene. (PDF) [file pone.0114372.s012.pdf]

Cftudor (C0619)

Run Setup

Run Information

Run Date: 2014/08/20 20:52
Run User: admin
Run Type: User-defined
Plate File: WR Plate_48 wells_SYBR_test.pltd
ID:
Notes:
Sample Volume: 10
Temperature Control Mode: Calculated
Lid Temperature: 100
Base Serial Number: MM010818
Optical Head Serial Number: MO005400

Protocol

1: 95.0°C for 1:00
2: 95.0°C for 0:15
3: 65.0°C for 0:15
4: 72.0°C for 0:30
Plate Read
5: GOTO 2, 39 more times
6: 95.0°C for 0:15
7: Melt Curve 65.0°C to 95.0°C: Increment 0.5°C 0:05
Plate Read

Plate Display

	1	2	3	4	5	6
A	Std-1<br>SYBR	Std-1<br>SYBR	Std-1<br>SYBR	Std-2<br>SYBR	Std-2<br>SYBR	Std-2<br>SYBR
B	Unk<br>SYBR<br>Male Head	Unk<br>SYBR<br>Male Abdomen	Unk<br>SYBR<br>Female Head	Unk<br>SYBR<br>Female Abdomen	Unk<br>SYBR<br>0h/1h cleavage	Unk<br>SYBR<br>0h/4h cleavage
C	Unk<br>SYBR<br>0h/8h cleavage	Unk<br>SYBR<br>0h/12h morula	Unk<br>SYBR<br>4h/12h morula	Unk<br>SYBR<br>8h/16h morula	Unk<br>SYBR<br>16h/24h morula	
D	Unk<br>SYBR<br>Male Head	Unk<br>SYBR<br>Male Abdomen	Unk<br>SYBR<br>Female Head	Unk<br>SYBR<br>Female Abdomen	Unk<br>SYBR<br>0h/1h cleavage	Unk<br>SYBR<br>0h/4h cleavage
E	Unk<br>SYBR<br>0h/8h cleavage	Unk<br>SYBR<br>0h/12h morula	Unk<br>SYBR<br>4h/12h morula	Unk<br>SYBR<br>8h/16h morula	Unk<br>SYBR<br>16h/24h morula	
F	Unk<br>SYBR<br>Male Head	Unk<br>SYBR<br>Male Abdomen	Unk<br>SYBR<br>Female Head	Unk<br>SYBR<br>Female Abdomen	Unk<br>SYBR<br>0h/1h cleavage	Unk<br>SYBR<br>0h/4h cleavage
G	Unk<br>SYBR<br>0h/8h cleavage	Unk<br>SYBR<br>0h/12h morula	Unk<br>SYBR<br>4h/12h morula	Unk<br>SYBR<br>8h/16h morula	Unk<br>SYBR<br>16h/24h morula	
H	Std-3<br>SYBR	Std-3<br>SYBR	Std-3<br>SYBR	Std-4<br>SYBR	Std-4<br>SYBR	Std-4<br>SYBR

Quantification

Step #: 4
Analysis Mode: Fluorophore
Cq Determination: Single Threshold
Baseline Method:
SYBR: Auto Calculated
Threshold Setting:
SYBR: .08, Auto Calculated

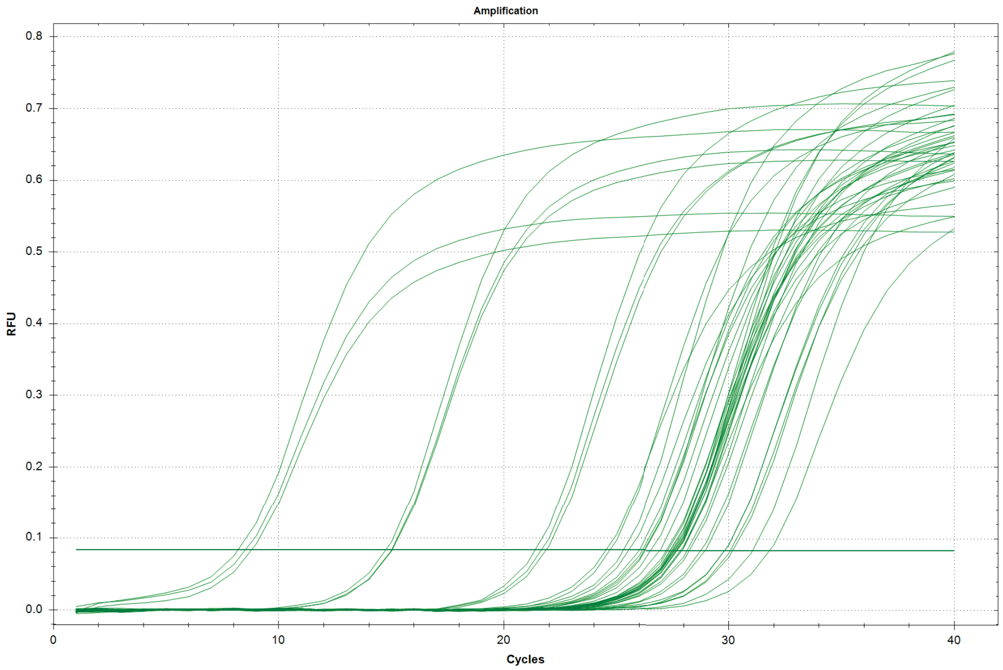

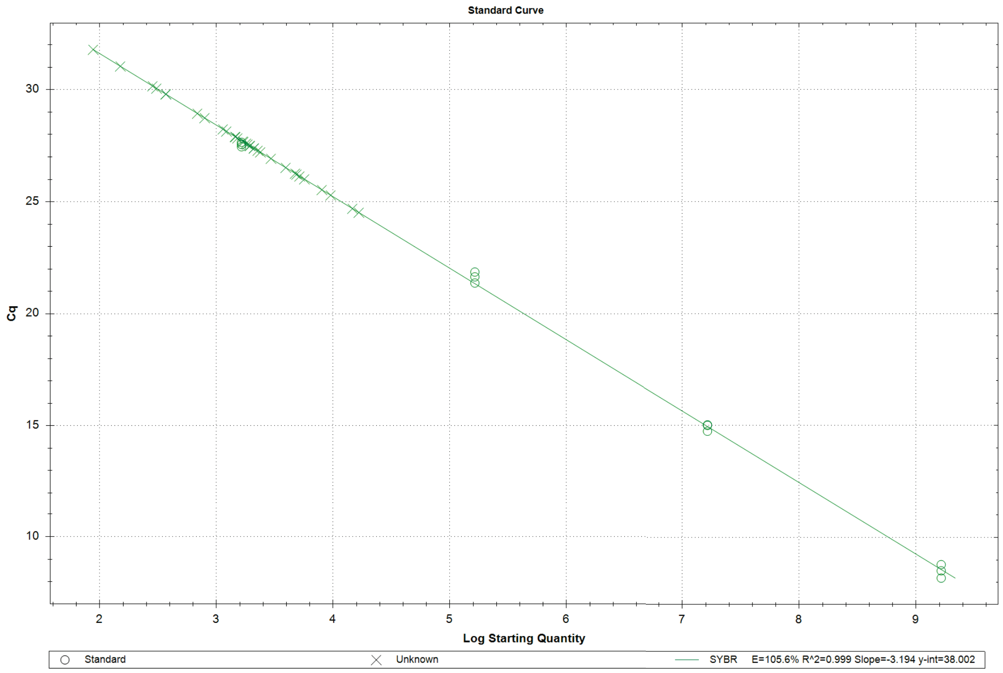

Quantification Data

Well	Fluor	Target	Content	Sample	Cq	Cq Mean	Cq Std. Dev	Starting Quantity (SQ)	Log Starting Quantity	SQ Mean	SQ Std. Dev
A01	SYBR		Std-1		8.19	8.50	0.301	1.640E+09	9.215	1.64E+09	0.00E+00
A02	SYBR		Std-1		8.79	8.50	0.301	1.640E+09	9.215	1.64E+09	0.00E+00
A03	SYBR		Std-1		8.51	8.50	0.301	1.640E+09	9.215	1.64E+09	0.00E+00
A04	SYBR		Std-2		15.01	14.93	0.159	1.640E+07	7.215	1.64E+07	0.00E+00
A05	SYBR		Std-2		15.03	14.93	0.159	1.640E+07	7.215	1.64E+07	0.00E+00
A06	SYBR		Std-2		14.75	14.93	0.159	1.640E+07	7.215	1.64E+07	0.00E+00
B01	SYBR		Unkn	Male Head	25.29	25.29	0.000	9.526E+03	3.979	9.53E+03	0.00E+00
B02	SYBR		Unkn	Male Abdomen	31.79	31.79	0.000	8.788E+01	1.944	8.79E+01	0.00E+00
B03	SYBR		Unkn	Female Head	24.52	24.52	0.000	1.663E+04	4.221	1.66E+04	0.00E+00
B04	SYBR		Unkn	Female Abdomen	27.39	27.39	0.000	2.103E+03	3.323	2.10E+03	0.00E+00
B05	SYBR		Unkn	0h/1h cleavage	27.84	27.84	0.000	1.520E+03	3.182	1.52E+03	0.00E+00
B06	SYBR		Unkn	0h/4h cleavage	24.70	24.70	0.000	1.466E+04	4.166	1.47E+04	0.00E+00
C01	SYBR		Unkn	0h/8h cleavage	26.26	26.26	0.000	4.733E+03	3.675	4.73E+03	0.00E+00
C02	SYBR		Unkn	0h/12h morula	27.22	27.22	0.000	2.383E+03	3.377	2.38E+03	0.00E+00
C03	SYBR		Unkn	4h/12h morula	27.39	27.39	0.000	2.105E+03	3.323	2.10E+03	0.00E+00
C04	SYBR		Unkn	8h/16h morula	26.14	26.14	0.000	5.171E+03	3.714	5.17E+03	0.00E+00
C05	SYBR		Unkn	16h/24h morula	27.90	27.90	0.000	1.459E+03	3.164	1.46E+03	0.00E+00
D01	SYBR		Unkn	Male Head	30.17	30.17	0.000	2.843E+02	2.454	2.84E+02	0.00E+00
D02	SYBR		Unkn	Male Abdomen	30.06	30.06	0.000	3.066E+02	2.487	3.07E+02	0.00E+00
D03	SYBR		Unkn	Female Head	27.56	27.56	0.000	1.855E+03	3.268	1.85E+03	0.00E+00
D04	SYBR		Unkn	Female Abdomen	28.24	28.24	0.000	1.139E+03	3.056	1.14E+03	0.00E+00
D05	SYBR		Unkn	0h/1h cleavage	28.94	28.94	0.000	6.869E+02	2.837	6.87E+02	0.00E+00
D06	SYBR		Unkn	0h/4h cleavage	27.29	27.29	0.000	2.264E+03	3.355	2.26E+03	0.00E+00
E01	SYBR		Unkn	0h/8h cleavage	27.50	27.50	0.000	1.946E+03	3.289	1.95E+03	0.00E+00
E02	SYBR		Unkn	0h/12h morula	27.51	27.51	0.000	1.934E+03	3.286	1.93E+03	0.00E+00
E03	SYBR		Unkn	4h/12h morula	28.74	28.74	0.000	7.930E+02	2.899	7.93E+02	0.00E+00
E04	SYBR		Unkn	8h/16h morula	29.80	29.80	0.000	3.693E+02	2.567	3.69E+02	0.00E+00
E05	SYBR		Unkn	16h/24h morula	25.53	25.53	0.000	8.014E+03	3.904	8.01E+03	0.00E+00
F01	SYBR		Unkn	Male Head	26.52	26.52	0.000	3.929E+03	3.594	3.93E+03	0.00E+00
F02	SYBR		Unkn	Male Abdomen	27.91	27.91	0.000	1.447E+03	3.161	1.45E+03	0.00E+00

Quantification Data

Well	Fluor	Target	Content	Sample	Cq	Cq Mean	Cq Std. Dev	Starting Quantity (SQ)	Log Starting Quantity	SQ Mean	SQ Std. Dev
F03	SYBR		Unkn	Female Head	27.68	27.68	0.000	1.710E+03	3.233	1.71E+03	0.00E+00
F04	SYBR		Unkn	Female Abdomen	26.93	26.93	0.000	2.934E+03	3.467	2.93E+03	0.00E+00
F05	SYBR		Unkn	0h/1h cleavage	26.01	26.01	0.000	5.679E+03	3.754	5.68E+03	0.00E+00
F06	SYBR		Unkn	0h/4h cleavage	26.22	26.22	0.000	4.870E+03	3.688	4.87E+03	0.00E+00
G01	SYBR		Unkn	0h/8h cleavage	27.70	27.70	0.000	1.684E+03	3.226	1.68E+03	0.00E+00
G02	SYBR		Unkn	0h/12h morula	31.05	31.05	0.000	1.502E+02	2.177	1.50E+02	0.00E+00
G03	SYBR		Unkn	4h/12h morula	28.14	28.14	0.000	1.220E+03	3.086	1.22E+03	0.00E+00
G04	SYBR		Unkn	8h/16h morula	27.60	27.60	0.000	1.802E+03	3.256	1.80E+03	0.00E+00
G05	SYBR		Unkn	16h/24h morula	29.80	29.80	0.000	3.687E+02	2.567	3.69E+02	0.00E+00
H01	SYBR		Std-3		21.65	21.63	0.245	1.640E+05	5.215	1.64E+05	0.00E+00
H02	SYBR		Std-3		21.37	21.63	0.245	1.640E+05	5.215	1.64E+05	0.00E+00
H03	SYBR		Std-3		21.86	21.63	0.245	1.640E+05	5.215	1.64E+05	0.00E+00
H04	SYBR		Std-4		27.46	27.56	0.099	1.640E+03	3.215	1.64E+03	0.00E+00
H05	SYBR		Std-4		27.65	27.56	0.099	1.640E+03	3.215	1.64E+03	0.00E+00
H06	SYBR		Std-4		27.56	27.56	0.099	1.640E+03	3.215	1.64E+03	0.00E+00

Melt Curve

Step #: 7

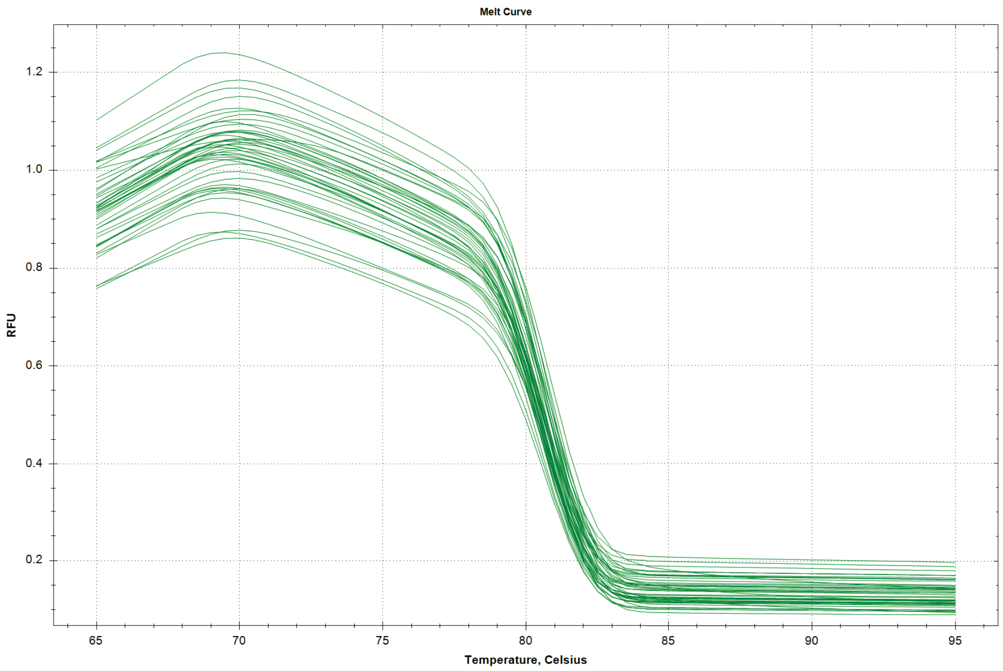

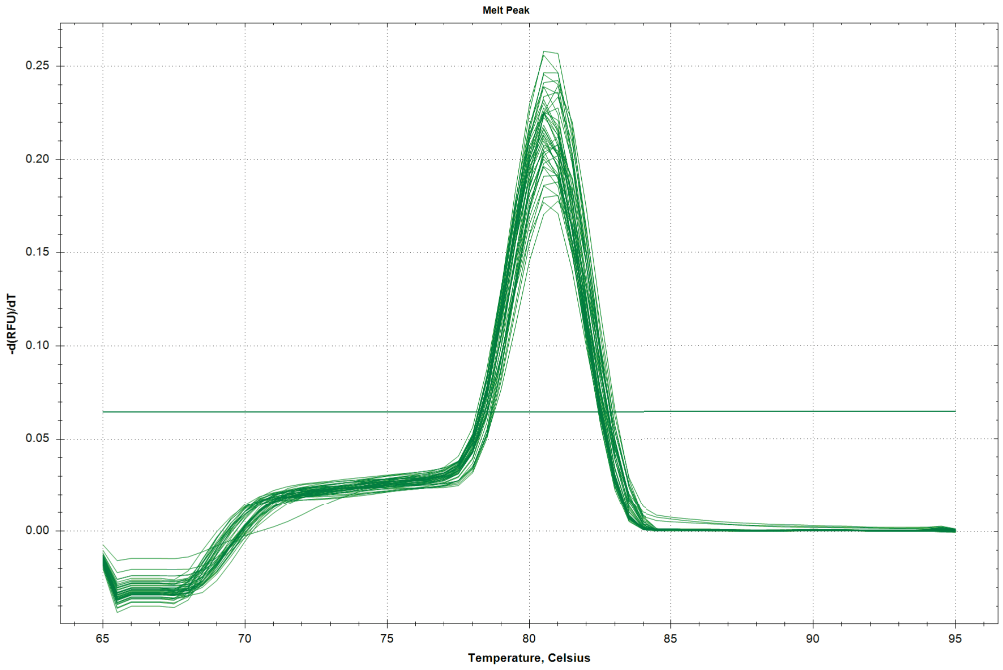

Melt Curve Data

Well	Fluor	Target	Content	Sample	Melt Temp
A01	SYBR		Std-1		81.00
A02	SYBR		Std-1		81.00
A03	SYBR		Std-1		81.00
A04	SYBR		Std-2		81.00
A05	SYBR		Std-2		81.00
A06	SYBR		Std-2		81.00
B01	SYBR		Unkn	Male Head	80.50
B02	SYBR		Unkn	Male Abdomen	80.50
B03	SYBR		Unkn	Female Head	81.00
B04	SYBR		Unkn	Female Abdomen	80.50
B05	SYBR		Unkn	0h/1h cleavage	80.50
B06	SYBR		Unkn	0h/4h cleavage	81.00
C01	SYBR		Unkn	0h/8h cleavage	80.50
C02	SYBR		Unkn	0h/12h morula	80.50
C03	SYBR		Unkn	4h/12h morula	81.00
C04	SYBR		Unkn	8h/16h morula	80.50
C05	SYBR		Unkn	16h/24h morula	80.50
D01	SYBR		Unkn	Male Head	80.50

Melt Curve Data

Well	Fluor	Target	Content	Sample	Melt Temp
D02	SYBR		Unkn	Male Abdomen	80.50
D03	SYBR		Unkn	Female Head	80.50
D04	SYBR		Unkn	Female Abdomen	81.00
D05	SYBR		Unkn	0h/1h cleavage	80.50
D06	SYBR		Unkn	0h/4h cleavage	80.50
E01	SYBR		Unkn	0h/8h cleavage	80.50
E02	SYBR		Unkn	0h/12h morula	80.50
E03	SYBR		Unkn	4h/12h morula	80.50
E04	SYBR		Unkn	8h/16h morula	80.50
E05	SYBR		Unkn	16h/24h morula	80.50
F01	SYBR		Unkn	Male Head	80.50
F02	SYBR		Unkn	Male Abdomen	80.50
F03	SYBR		Unkn	Female Head	80.50
F04	SYBR		Unkn	Female Abdomen	80.50
F05	SYBR		Unkn	0h/1h cleavage	80.50
F06	SYBR		Unkn	0h/4h cleavage	81.00
G01	SYBR		Unkn	0h/8h cleavage	80.50
G02	SYBR		Unkn	0h/12h morula	80.50
G03	SYBR		Unkn	4h/12h morula	80.50
G04	SYBR		Unkn	8h/16h morula	80.50
G05	SYBR		Unkn	16h/24h morula	80.50
H01	SYBR		Std-3		81.00
H02	SYBR		Std-3		81.00
H03	SYBR		Std-3		80.50
H04	SYBR		Std-4		80.50
H05	SYBR		Std-4		81.00
H06	SYBR		Std-4		80.50

End Point

Fluorophore/Target: SYBR

End Cycles to Average: 5
Mode: Percentage of Range - 10

Lowest RFU Value: 0.473
Highest RFU Value: 0.760
Cut Off Value:

SYBR End Point Data

Well	Fluor	Content	Sample	End RFU	Call
A01	SYBR	Std-1		0.668	
A02	SYBR	Std-1		0.529	
A03	SYBR	Std-1		0.551	
A04	SYBR	Std-2		0.639	
A05	SYBR	Std-2		0.627	
A06	SYBR	Std-2		0.705	
B01	SYBR	Unkn	Male Head	0.760	
B02	SYBR	Unkn	Male Abdomen	0.473	
B03	SYBR	Unkn	Female Head	0.559	
B04	SYBR	Unkn	Female Abdomen	0.595	
B05	SYBR	Unkn	0h/1h cleavage	0.531	
B06	SYBR	Unkn	0h/4h cleavage	0.682	
C01	SYBR	Unkn	0h/8h cleavage	0.712	
C02	SYBR	Unkn	0h/12h morula	0.585	
C03	SYBR	Unkn	4h/12h morula	0.633	
C04	SYBR	Unkn	8h/16h morula	0.602	
C05	SYBR	Unkn	16h/24h morula	0.643	
D01	SYBR	Unkn	Male Head	0.586	
D02	SYBR	Unkn	Male Abdomen	0.566	
D03	SYBR	Unkn	Female Head	0.616	
D04	SYBR	Unkn	Female Abdomen	0.601	
D05	SYBR	Unkn	0h/1h cleavage	0.622	
D06	SYBR	Unkn	0h/4h cleavage	0.640	
E01	SYBR	Unkn	0h/8h cleavage	0.570	
E02	SYBR	Unkn	0h/12h morula	0.613	
E03	SYBR	Unkn	4h/12h morula	0.594	
E04	SYBR	Unkn	8h/16h morula	0.587	
E05	SYBR	Unkn	16h/24h morula	0.588	
F01	SYBR	Unkn	Male Head	0.625	

SYBR End Point Data

Well	Fluor	Content	Sample	End RFU	Call
F02	SYBR	Unkn	Male Abdomen	0.659	
F03	SYBR	Unkn	Female Head	0.596	
F04	SYBR	Unkn	Female Abdomen	0.655	
F05	SYBR	Unkn	0h/1h cleavage	0.633	
F06	SYBR	Unkn	0h/4h cleavage	0.637	
G01	SYBR	Unkn	0h/8h cleavage	0.637	
G02	SYBR	Unkn	0h/12h morula	0.591	
G03	SYBR	Unkn	4h/12h morula	0.647	
G04	SYBR	Unkn	8h/16h morula	0.678	
G05	SYBR	Unkn	16h/24h morula	0.596	
H01	SYBR	Std-3		0.679	
H02	SYBR	Std-3		0.734	
H03	SYBR	Std-3		0.684	
H04	SYBR	Std-4		0.740	
H05	SYBR	Std-4		0.749	
H06	SYBR	Std-4		0.700	

QC Parameters

Data

Description	Value	Use	Results	Exclude Wells	All excluded wells
Negative control with a Cq less than	38	True		False	
NTC with a Cq less than	38	True		False	
NRT with a Cq less than	38	True		False	
Positive control with a Cq greater than	30	True		False	
Unknown without a Cq	N/A	True		False	
Standard without a Cq	N/A	True		False	
Efficiency greater than	110.0	True			

Data

Description	Value	Use	Results	Exclude Wells	All excluded wells
Efficiency less than	90.0	True			
Std Curve R^2 less than	0.980	True			
Replicate group Cq Std Dev greater than	0.20	True	SYBR:A1, A2, A3, H1, H2, H3.	False	

Cftp53i13 (C0663)

Run Setup

Run Information

Run Date: 2014/08/19 20:17
Run User: admin
Run Type: User-defined
Plate File: WR Plate_48 wells_SYBR_test.pltd
ID:
Notes:
Sample Volume: 10
Temperature Control Mode: Calculated
Lid Temperature: 100
Base Serial Number: MM010818
Optical Head Serial Number: MO005400

Protocol

1: 95.0°C for 1:00
2: 95.0°C for 0:15
3: 55.0°C for 0:15
4: 72.0°C for 0:30
Plate Read
5: GOTO 2, 39 more times
6: 95.0°C for 0:15
7: Melt Curve 65.0°C to 95.0°C: Increment 0.5°C 0:05
Plate Read

Plate Display

	1	2	3	4	5	6
A	Std-1<br>SYBR<br>C0663rtF2rtR1	Std-1<br>SYBR<br>C0663rtF2rtR1	Std-1<br>SYBR<br>C0663rtF2rtR1	Std-2<br>SYBR<br>C0663rtF2rtR1	Std-2<br>SYBR<br>C0663rtF2rtR1	Std-2<br>SYBR<br>C0663rtF2rtR1
B	Unk<br>SYBR<br>Male Head	Unk<br>SYBR<br>Male Abdomen	Unk<br>SYBR<br>Female Head	Unk<br>SYBR<br>Female Abdomen	Unk<br>SYBR<br>0h/1h cleavage	Unk<br>SYBR<br>0h/4h cleavage
C	Unk<br>SYBR<br>0h/8h cleavage	Unk<br>SYBR<br>0h/12h morula	Unk<br>SYBR<br>4h/12h morula	Unk<br>SYBR<br>8h/16h morula	Unk<br>SYBR<br>16h/24h morula	
D	Unk<br>SYBR<br>Male Head	Unk<br>SYBR<br>Male Abdomen	Unk<br>SYBR<br>Female Head	Unk<br>SYBR<br>Female Abdomen	Unk<br>SYBR<br>0h/1h cleavage	Unk<br>SYBR<br>0h/4h cleavage
E	Unk<br>SYBR<br>0h/8h cleavage	Unk<br>SYBR<br>0h/12h morula	Unk<br>SYBR<br>4h/12h morula	Unk<br>SYBR<br>8h/16h morula	Unk<br>SYBR<br>16h/24h morula	
F	Unk<br>SYBR<br>Male Head	Unk<br>SYBR<br>Male Abdomen	Unk<br>SYBR<br>Female Head	Unk<br>SYBR<br>Female Abdomen	Unk<br>SYBR<br>0h/1h cleavage	Unk<br>SYBR<br>0h/4h cleavage
G	Unk<br>SYBR<br>0h/8h cleavage	Unk<br>SYBR<br>0h/12h morula	Unk<br>SYBR<br>4h/12h morula	Unk<br>SYBR<br>8h/16h morula	Unk<br>SYBR<br>16h/24h morula	
H	Std-3<br>SYBR<br>C0663rtF2rtR1	Std-3<br>SYBR<br>C0663rtF2rtR1	Std-3<br>SYBR<br>C0663rtF2rtR1	Std-4<br>SYBR<br>C0663rtF2rtR1	Std-4<br>SYBR<br>C0663rtF2rtR1	Std-4<br>SYBR<br>C0663rtF2rtR1

Quantification

Step #: 4
Analysis Mode: Fluorophore
Cq Determination: Single Threshold
Baseline Method:
SYBR: Auto Calculated
Threshold Setting:
SYBR: .10, Auto Calculated

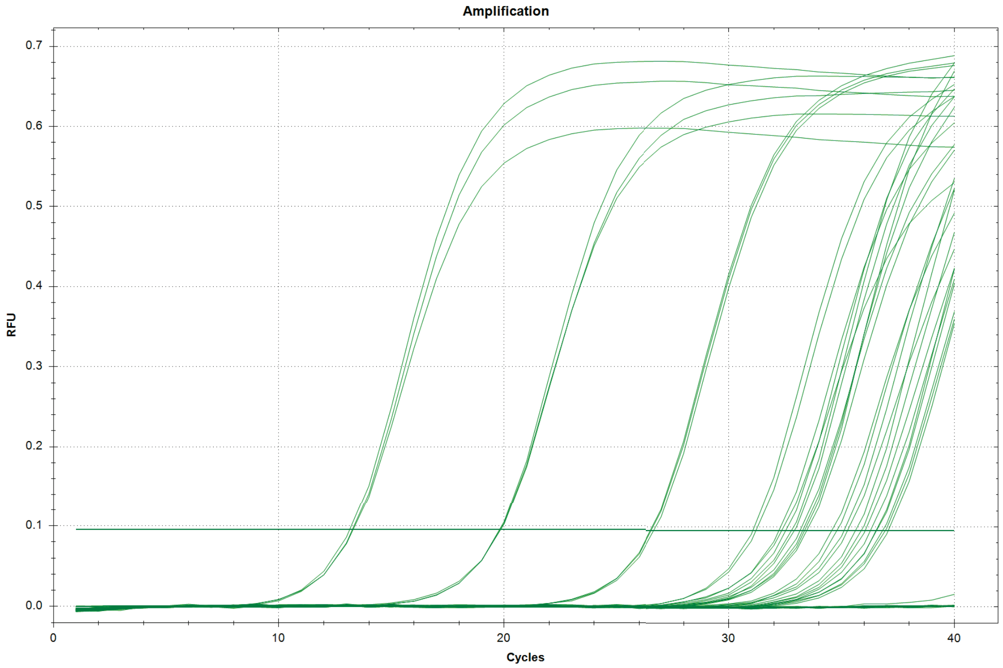

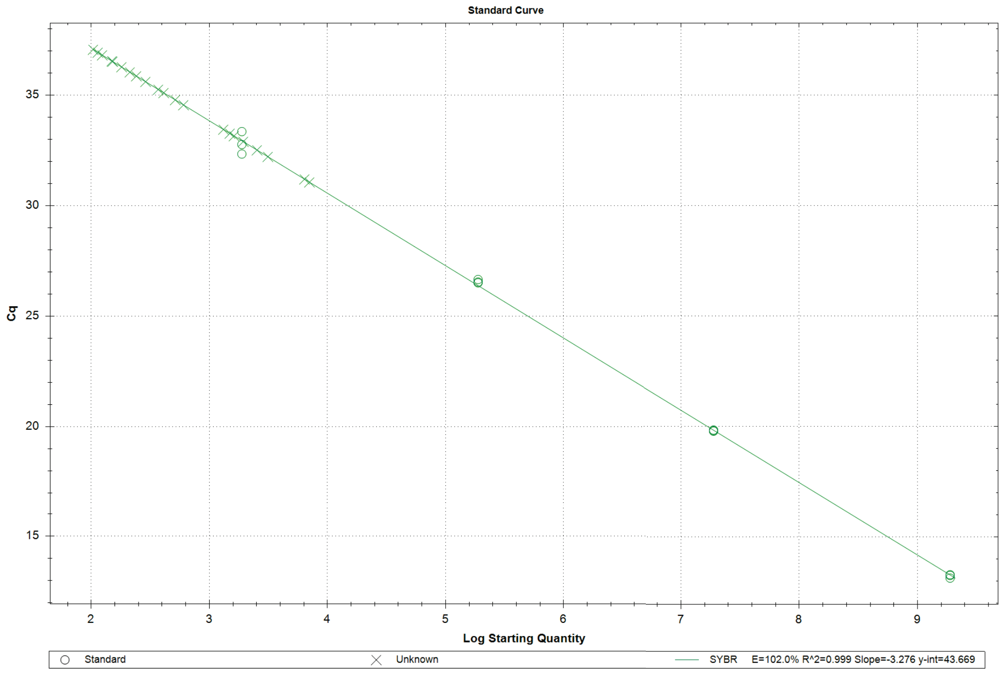

Quantification Data

Well	Fluor	Target	Content	Sample	Cq	Cq Mean	Cq Std. Dev	Starting Quantity (SQ)	Log Starting Quantity	SQ Mean	SQ Std. Dev
A01	SYBR		Std-1	C0663rtF2rtR1	13.14	13.23	0.080	1.885E+09	9.275	1.89E+09	0.00E+00
A02	SYBR		Std-1	C0663rtF2rtR1	13.26	13.23	0.080	1.885E+09	9.275	1.89E+09	0.00E+00
A03	SYBR		Std-1	C0663rtF2rtR1	13.30	13.23	0.080	1.885E+09	9.275	1.89E+09	0.00E+00
A04	SYBR		Std-2	C0663rtF2rtR1	19.82	19.82	0.027	1.885E+07	7.275	1.89E+07	0.00E+00
A05	SYBR		Std-2	C0663rtF2rtR1	19.79	19.82	0.027	1.885E+07	7.275	1.89E+07	0.00E+00
A06	SYBR		Std-2	C0663rtF2rtR1	19.84	19.82	0.027	1.885E+07	7.275	1.89E+07	0.00E+00
B01	SYBR		Unkn	Male Head	N/A	0.00	0.000	N/A	N/A	0.00E+00	0.00E+00
B02	SYBR		Unkn	Male Abdomen	36.93	36.93	0.000	1.140E+02	2.057	1.14E+02	0.00E+00
B03	SYBR		Unkn	Female Head	35.26	35.26	0.000	3.696E+02	2.568	3.70E+02	0.00E+00
B04	SYBR		Unkn	Female Abdomen	36.04	36.04	0.000	2.130E+02	2.328	2.13E+02	0.00E+00
B05	SYBR		Unkn	0h/1h cleavage	37.06	37.06	0.000	1.039E+02	2.017	1.04E+02	0.00E+00
B06	SYBR		Unkn	0h/4h cleavage	35.86	35.86	0.000	2.413E+02	2.382	2.41E+02	0.00E+00
C01	SYBR		Unkn	0h/8h cleavage	35.61	35.61	0.000	2.887E+02	2.460	2.89E+02	0.00E+00
C02	SYBR		Unkn	0h/12h morula	N/A	0.00	0.000	N/A	N/A	0.00E+00	0.00E+00
C03	SYBR		Unkn	4h/12h morula	32.22	32.22	0.000	3.133E+03	3.496	3.13E+03	0.00E+00
C04	SYBR		Unkn	8h/16h morula	36.81	36.81	0.000	1.241E+02	2.094	1.24E+02	0.00E+00
C05	SYBR		Unkn	16h/24h morula	N/A	0.00	0.000	N/A	N/A	0.00E+00	0.00E+00
D01	SYBR		Unkn	Male Head	32.90	32.90	0.000	1.939E+03	3.287	1.94E+03	0.00E+00
D02	SYBR		Unkn	Male Abdomen	31.06	31.06	0.000	7.047E+03	3.848	7.05E+03	0.00E+00
D03	SYBR		Unkn	Female Head	36.27	36.27	0.000	1.809E+02	2.257	1.81E+02	0.00E+00
D04	SYBR		Unkn	Female Abdomen	31.20	31.20	0.000	6.400E+03	3.806	6.40E+03	0.00E+00
D05	SYBR		Unkn	0h/1h cleavage	36.52	36.52	0.000	1.520E+02	2.182	1.52E+02	0.00E+00
D06	SYBR		Unkn	0h/4h cleavage	34.78	34.78	0.000	5.151E+02	2.712	5.15E+02	0.00E+00
E01	SYBR		Unkn	0h/8h cleavage	N/A	0.00	0.000	N/A	N/A	0.00E+00	0.00E+00
E02	SYBR		Unkn	0h/12h morula	N/A	0.00	0.000	N/A	N/A	0.00E+00	0.00E+00
E03	SYBR		Unkn	4h/12h morula	N/A	0.00	0.000	N/A	N/A	0.00E+00	0.00E+00
E04	SYBR		Unkn	8h/16h morula	36.54	36.54	0.000	1.497E+02	2.175	1.50E+02	0.00E+00
E05	SYBR		Unkn	16h/24h morula	N/A	0.00	0.000	N/A	N/A	0.00E+00	0.00E+00
F01	SYBR		Unkn	Male Head	N/A	0.00	0.000	N/A	N/A	0.00E+00	0.00E+00
F02	SYBR		Unkn	Male Abdomen	32.52	32.52	0.000	2.535E+03	3.404	2.54E+03	0.00E+00
F03	SYBR		Unkn	Female Head	34.56	34.56	0.000	6.049E+02	2.782	6.05E+02	0.00E+00
F04	SYBR		Unkn	Female Abdomen	33.27	33.27	0.000	1.493E+03	3.174	1.49E+03	0.00E+00
F05	SYBR		Unkn	0h/1h cleavage	33.16	33.16	0.000	1.619E+03	3.209	1.62E+03	0.00E+00
F06	SYBR		Unkn	0h/4h cleavage	35.11	35.11	0.000	4.111E+02	2.614	4.11E+02	0.00E+00
G01	SYBR		Unkn	0h/8h cleavage	33.45	33.45	0.000	1.317E+03	3.120	1.32E+03	0.00E+00
G02	SYBR		Unkn	0h/12h morula	N/A	0.00	0.000	N/A	N/A	0.00E+00	0.00E+00
G03	SYBR		Unkn	4h/12h morula	N/A	0.00	0.000	N/A	N/A	0.00E+00	0.00E+00
G04	SYBR		Unkn	8h/16h morula	N/A	0.00	0.000	N/A	N/A	0.00E+00	0.00E+00
G05	SYBR		Unkn	16h/24h morula	N/A	0.00	0.000	N/A	N/A	0.00E+00	0.00E+00
H01	SYBR		Std-3	C0663rtF2rtR1	26.51	26.57	0.078	1.885E+05	5.275	1.89E+05	0.00E+00
H02	SYBR		Std-3	C0663rtF2rtR1	26.55	26.57	0.078	1.885E+05	5.275	1.89E+05	0.00E+00
H03	SYBR		Std-3	C0663rtF2rtR1	26.66	26.57	0.078	1.885E+05	5.275	1.89E+05	0.00E+00
H04	SYBR		Std-4	C0663rtF2rtR1	32.77	32.82	0.509	1.885E+03	3.275	1.89E+03	0.00E+00

Quantification Data

Well	Fluor	Target	Content	Sample	Cq	Cq Mean	Cq Std. Dev	Starting Quantity (SQ)	Log Starting Quantity	SQ Mean	SQ Std. Dev
H05	SYBR		Std-4	C0663rtF2rtR1	33.35	32.82	0.509	1.885E+03	3.275	1.89E+03	0.00E+00
H06	SYBR		Std-4	C0663rtF2rtR1	32.34	32.82	0.509	1.885E+03	3.275	1.89E+03	0.00E+00

Melt Curve

Step #: 7

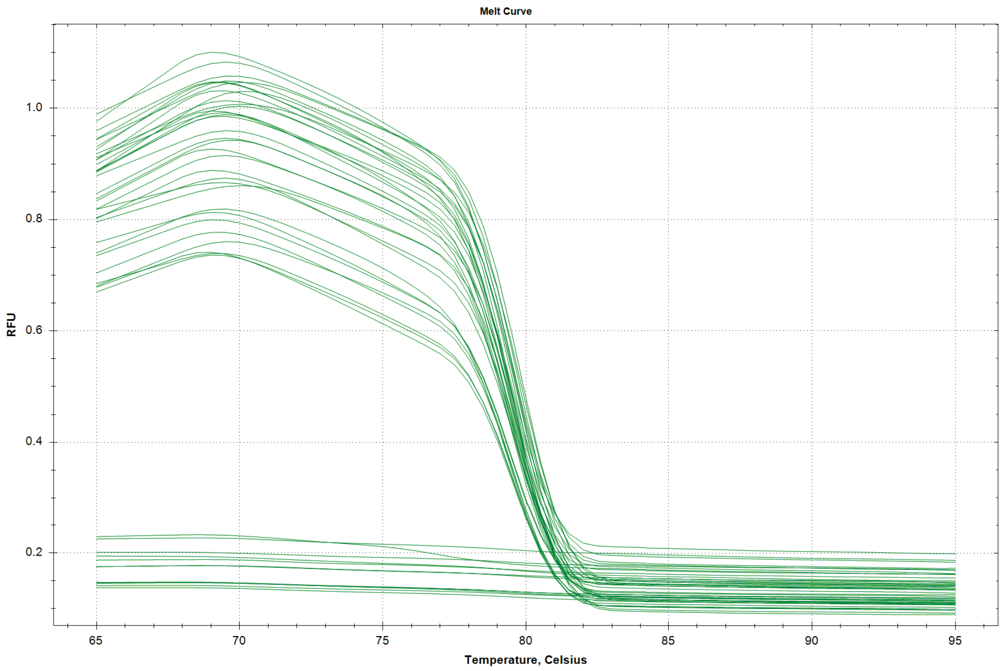

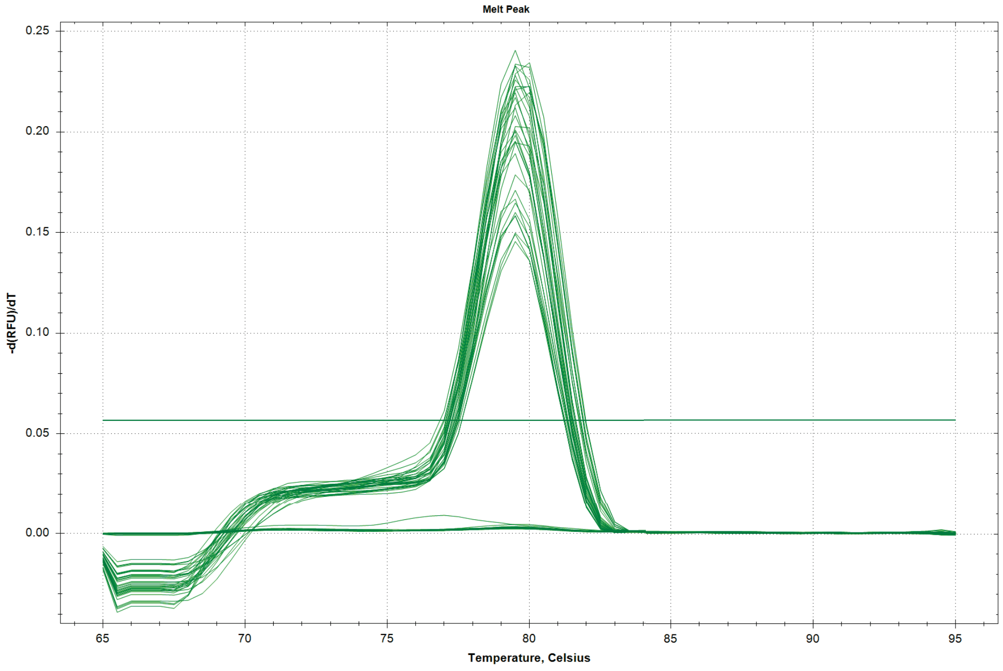

Melt Curve Data

Well	Fluor	Target	Content	Sample	Melt Temp
A01	SYBR		Std-1	C0663rtF2rtR1	80.00
A02	SYBR		Std-1	C0663rtF2rtR1	79.50
A03	SYBR		Std-1	C0663rtF2rtR1	79.50
A04	SYBR		Std-2	C0663rtF2rtR1	79.50
A05	SYBR		Std-2	C0663rtF2rtR1	80.00
A06	SYBR		Std-2	C0663rtF2rtR1	80.00
B01	SYBR		Unkn	Male Head	None
B02	SYBR		Unkn	Male Abdomen	79.50
B03	SYBR		Unkn	Female Head	79.50
B04	SYBR		Unkn	Female Abdomen	79.50
B05	SYBR		Unkn	0h/1h cleavage	79.50
B06	SYBR		Unkn	0h/4h cleavage	79.50
C01	SYBR		Unkn	0h/8h cleavage	79.50
C02	SYBR		Unkn	0h/12h morula	None
C03	SYBR		Unkn	4h/12h morula	79.50
C04	SYBR		Unkn	8h/16h morula	79.50
C05	SYBR		Unkn	16h/24h morula	None
D01	SYBR		Unkn	Male Head	79.50
D02	SYBR		Unkn	Male Abdomen	79.50
D03	SYBR		Unkn	Female Head	79.50
D04	SYBR		Unkn	Female Abdomen	79.50
D05	SYBR		Unkn	0h/1h cleavage	79.50
D06	SYBR		Unkn	0h/4h cleavage	79.50
E01	SYBR		Unkn	0h/8h cleavage	None
E02	SYBR		Unkn	0h/12h morula	None
E03	SYBR		Unkn	4h/12h morula	None
E04	SYBR		Unkn	8h/16h morula	79.50
E05	SYBR		Unkn	16h/24h morula	None
F01	SYBR		Unkn	Male Head	None
F02	SYBR		Unkn	Male Abdomen	79.50
F03	SYBR		Unkn	Female Head	79.50
F04	SYBR		Unkn	Female Abdomen	79.50
F05	SYBR		Unkn	0h/1h cleavage	79.50
F06	SYBR		Unkn	0h/4h cleavage	79.50
G01	SYBR		Unkn	0h/8h cleavage	79.50
G02	SYBR		Unkn	0h/12h morula	None
G03	SYBR		Unkn	4h/12h morula	None
G04	SYBR		Unkn	8h/16h morula	None
G05	SYBR		Unkn	16h/24h morula	None
H01	SYBR		Std-3	C0663rtF2rtR1	79.50
H02	SYBR		Std-3	C0663rtF2rtR1	79.50
H03	SYBR		Std-3	C0663rtF2rtR1	79.50
H04	SYBR		Std-4	C0663rtF2rtR1	79.50
H05	SYBR		Std-4	C0663rtF2rtR1	79.50

Melt Curve Data

Well	Fluor	Target	Content	Sample	Melt Temp
H06	SYBR		Std-4	C0663rtF2rtR1	79.50

End Point

Fluorophore/Target: SYBR

End Cycles to Average: 5
Mode: Percentage of Range **-** 10
Lowest RFU Value: -0.000390
Highest RFU Value: 0.677
Cut Off Value:

SYBR End Point Data

SYBR End Point Data

Well	Fluor	Content	Sample	End RFU	Call
A01	SYBR	Std-1	C0663rtF2rtR1	0.662	
A02	SYBR	Std-1	C0663rtF2rtR1	0.639	
A03	SYBR	Std-1	C0663rtF2rtR1	0.577	
A04	SYBR	Std-2	C0663rtF2rtR1	0.614	
A05	SYBR	Std-2	C0663rtF2rtR1	0.661	
A06	SYBR	Std-2	C0663rtF2rtR1	0.643	
B01	SYBR	Unkn	Male Head	-0.0000136	
B02	SYBR	Unkn	Male Abdomen	0.188	
B03	SYBR	Unkn	Female Head	0.299	
B04	SYBR	Unkn	Female Abdomen	0.251	
B05	SYBR	Unkn	0h/1h cleavage	0.180	
B06	SYBR	Unkn	0h/4h cleavage	0.279	
C01	SYBR	Unkn	0h/8h cleavage	0.312	
C02	SYBR	Unkn	0h/12h morula	0.000330	
C03	SYBR	Unkn	4h/12h morula	0.530	
C04	SYBR	Unkn	8h/16h morula	0.195	
C05	SYBR	Unkn	16h/24h morula	-0.000390	
D01	SYBR	Unkn	Male Head	0.530	
D02	SYBR	Unkn	Male Abdomen	0.602	
D03	SYBR	Unkn	Female Head	0.232	
D04	SYBR	Unkn	Female Abdomen	0.584	
D05	SYBR	Unkn	0h/1h cleavage	0.225	
D06	SYBR	Unkn	0h/4h cleavage	0.360	
E01	SYBR	Unkn	0h/8h cleavage	0.00128	
E02	SYBR	Unkn	0h/12h morula	0.00157	
E03	SYBR	Unkn	4h/12h morula	0.000648	
E04	SYBR	Unkn	8h/16h morula	0.217	
E05	SYBR	Unkn	16h/24h morula	0.00116	
F01	SYBR	Unkn	Male Head	0.00764	
F02	SYBR	Unkn	Male Abdomen	0.554	
F03	SYBR	Unkn	Female Head	0.356	
F04	SYBR	Unkn	Female Abdomen	0.502	
F05	SYBR	Unkn	0h/1h cleavage	0.474	
F06	SYBR	Unkn	0h/4h cleavage	0.348	
G01	SYBR	Unkn	0h/8h cleavage	0.458	
G02	SYBR	Unkn	0h/12h morula	0.00133	
G03	SYBR	Unkn	4h/12h morula	0.00123	
G04	SYBR	Unkn	8h/16h morula	0.00117	
G05	SYBR	Unkn	16h/24h morula	0.00111	
H01	SYBR	Std-3	C0663rtF2rtR1	0.677	
H02	SYBR	Std-3	C0663rtF2rtR1	0.670	
H03	SYBR	Std-3	C0663rtF2rtR1	0.667	
H04	SYBR	Std-4	C0663rtF2rtR1	0.564	
H05	SYBR	Std-4	C0663rtF2rtR1	0.525	
H06	SYBR	Std-4	C0663rtF2rtR1	0.465	

QC Parameters

Data

Description	Value	Use	Results	Exclude Wells	All excluded wells
Negative control with a Cq less than	38	True		False	
NTC with a Cq less than	38	True		False	
NRT with a Cq less than	38	True		False	
Positive control with a Cq greater than	30	True		False	
Unknown without a Cq	N/A	True	SYBR:B1, C2, C5, E1, E2, E3, E5, F1, G2, G3, G4, G5.	False	
Standard without a Cq	N/A	True		False	
Efficiency greater than	110.0	True			
Efficiency less than	90.0	True			
Std Curve R^2 less than	0.980	True			
Replicate group Cq Std Dev greater than	0.20	True	SYBR:H4, H5, H6.	False	

Cfdbr-1 (M2053)

Run Setup

Run Information

Run Date: 2014/08/21 13:23
Run User: admin
Run Type: User-defined
Plate File: WR Plate_48 wells_SYBR_test.pltd
ID:
Notes:
Sample Volume: 10
Temperature Control Mode: Calculated
Lid Temperature: 100
Base Serial Number: MM010818
Optical Head Serial Number: MO005400

Protocol

1: 95.0°C for 1:00
2: 95.0°C for 0:15
3: 65.0°C for 0:15
4: 72.0°C for 0:30
Plate Read
5: GOTO 2, 39 more times
6: 95.0°C for 0:15
7: Melt Curve 65.0°C to 95.0°C: Increment 0.5°C 0:05
Plate Read

Plate Display

	1	2	3	4	5	6
A	Std<br>SYBR<br>M2053rtF2rtR2	Std<br>SYBR<br>M2053rtF2rtR2	Std<br>SYBR<br>M2053rtF2rtR2	Std<br>SYBR<br>M2053rtF2rtR2	Std<br>SYBR<br>M2053rtF2rtR2	Std<br>SYBR<br>M2053rtF2rtR2
B	Unk<br>SYBR<br>Male Head	Unk<br>SYBR<br>Male Abdomen	Unk<br>SYBR<br>Female Head	Unk<br>SYBR<br>Female Abdomen	Unk<br>SYBR<br>0h/1h cleavage	Unk<br>SYBR<br>0h/4h cleavage
C	Unk<br>SYBR<br>0h/8h cleavage	Unk<br>SYBR<br>0h/12h morula	Unk<br>SYBR<br>4h/12h morula	Unk<br>SYBR<br>8h/16h morula	Unk<br>SYBR<br>16h/24h morula	
D	Unk<br>SYBR<br>Male Head	Unk<br>SYBR<br>Male Abdomen	Unk<br>SYBR<br>Female Head	Unk<br>SYBR<br>Female Abdomen	Unk<br>SYBR<br>0h/1h cleavage	Unk<br>SYBR<br>0h/4h cleavage
E	Unk<br>SYBR<br>0h/8h cleavage	Unk<br>SYBR<br>0h/12h morula	Unk<br>SYBR<br>4h/12h morula	Unk<br>SYBR<br>8h/16h morula	Unk<br>SYBR<br>16h/24h morula	
F	Unk<br>SYBR<br>Male Head	Unk<br>SYBR<br>Male Abdomen	Unk<br>SYBR<br>Female Head	Unk<br>SYBR<br>Female Abdomen	Unk<br>SYBR<br>0h/1h cleavage	Unk<br>SYBR<br>0h/4h cleavage
G	Unk<br>SYBR<br>0h/8h cleavage	Unk<br>SYBR<br>0h/12h morula	Unk<br>SYBR<br>4h/12h morula	Unk<br>SYBR<br>8h/16h morula	Unk<br>SYBR<br>16h/24h morula	
H	Std<br>SYBR<br>M2053rtF2rtR2	Std<br>SYBR<br>M2053rtF2rtR2	Std<br>SYBR<br>M2053rtF2rtR2	Std<br>SYBR<br>M2053rtF2rtR2	Std<br>SYBR<br>M2053rtF2rtR2	Std<br>SYBR<br>M2053rtF2rtR2

Quantification

Step #: 4
Analysis Mode: Fluorophore
Cq Determination: Single Threshold
Baseline Method:
SYBR: Auto Calculated
Threshold Setting:
SYBR: .04, Auto Calculated

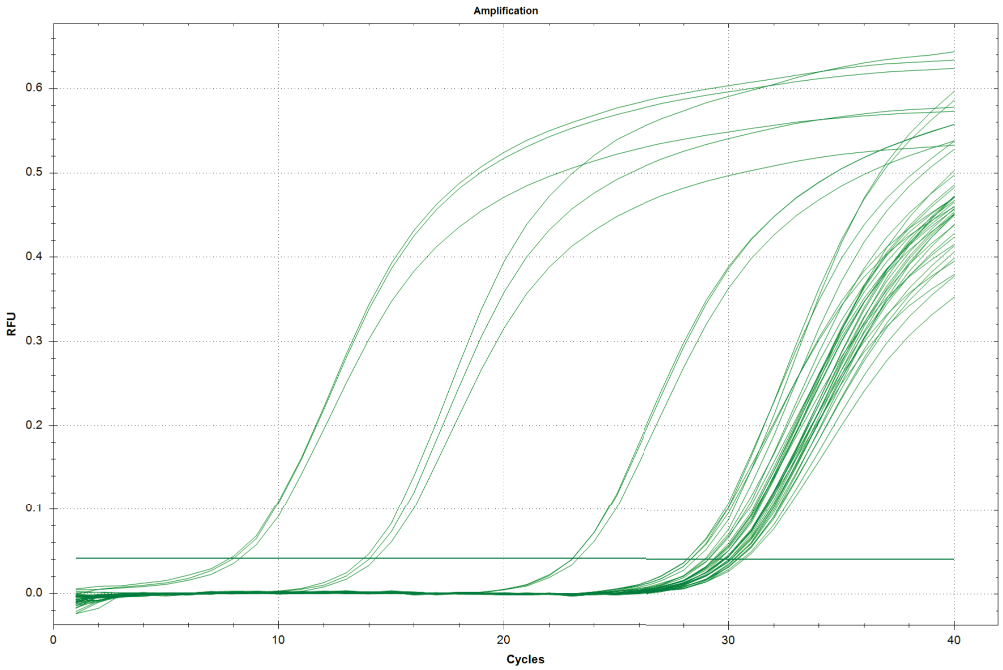

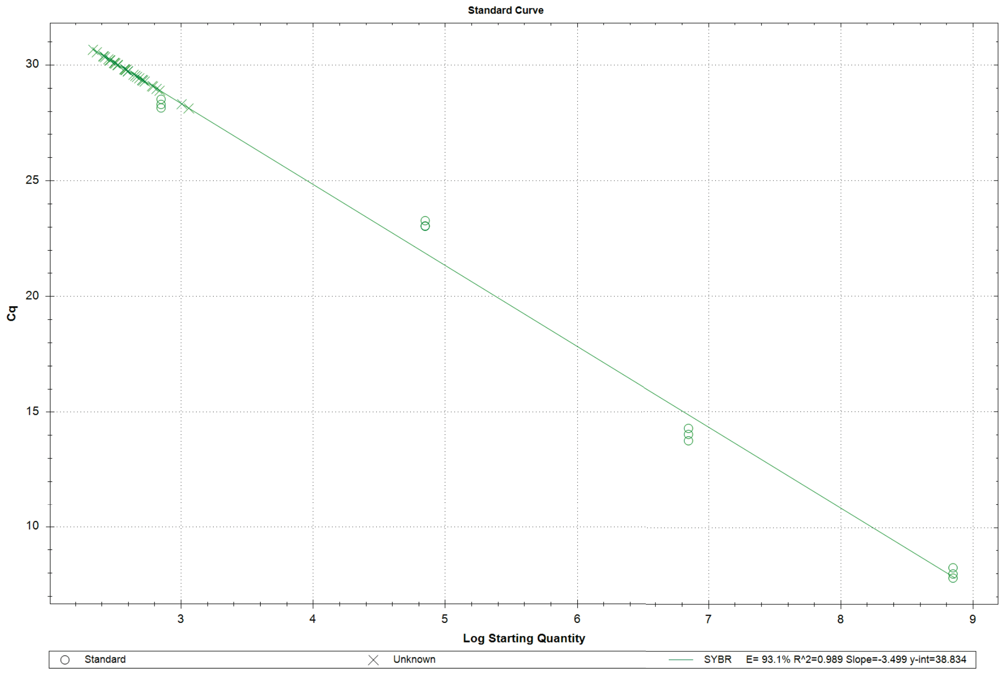

Quantification Data

Well	Fluor	Target	Content	Sample	Cq	Cq Mean	Cq Std. Dev	Starting Quantity (SQ)	Log Starting Quantity	SQ Mean	SQ Std. Dev
A01	SYBR		Std	M2053rtF2rtR2	7.82	7.82	0.000	7.040E+08	8.848	7.04E+08	0.00E+00
A02	SYBR		Std	M2053rtF2rtR2	8.00	8.00	0.000	7.040E+08	8.848	7.04E+08	0.00E+00
A03	SYBR		Std	M2053rtF2rtR2	8.27	8.27	0.000	7.040E+08	8.848	7.04E+08	0.00E+00
A04	SYBR		Std	M2053rtF2rtR2	14.29	14.29	0.000	7.040E+06	6.848	7.04E+06	0.00E+00
A05	SYBR		Std	M2053rtF2rtR2	14.03	14.03	0.000	7.040E+06	6.848	7.04E+06	0.00E+00
A06	SYBR		Std	M2053rtF2rtR2	13.76	13.76	0.000	7.040E+06	6.848	7.04E+06	0.00E+00
B01	SYBR		Unkn	Male Head	28.90	28.90	0.000	6.896E+02	2.839	6.90E+02	0.00E+00
B02	SYBR		Unkn	Male Abdomen	30.02	30.02	0.000	3.310E+02	2.520	3.31E+02	0.00E+00
B03	SYBR		Unkn	Female Head	29.80	29.80	0.000	3.810E+02	2.581	3.81E+02	0.00E+00
B04	SYBR		Unkn	Female Abdomen	29.74	29.74	0.000	3.978E+02	2.600	3.98E+02	0.00E+00
B05	SYBR		Unkn	0h/1h cleavage	29.36	29.36	0.000	5.093E+02	2.707	5.09E+02	0.00E+00
B06	SYBR		Unkn	0h/4h cleavage	29.59	29.59	0.000	4.380E+02	2.641	4.38E+02	0.00E+00
C01	SYBR		Unkn	0h/8h cleavage	29.30	29.30	0.000	5.318E+02	2.726	5.32E+02	0.00E+00
C02	SYBR		Unkn	0h/12h morula	29.44	29.44	0.000	4.843E+02	2.685	4.84E+02	0.00E+00
C03	SYBR		Unkn	4h/12h morula	30.22	30.22	0.000	2.895E+02	2.462	2.90E+02	0.00E+00
C04	SYBR		Unkn	8h/16h morula	29.36	29.36	0.000	5.089E+02	2.707	5.09E+02	0.00E+00
C05	SYBR		Unkn	16h/24h morula	30.21	30.21	0.000	2.919E+02	2.465	2.92E+02	0.00E+00
D01	SYBR		Unkn	Male Head	30.26	30.26	0.000	2.823E+02	2.451	2.82E+02	0.00E+00
D02	SYBR		Unkn	Male Abdomen	30.35	30.35	0.000	2.664E+02	2.426	2.66E+02	0.00E+00
D03	SYBR		Unkn	Female Head	30.38	30.38	0.000	2.610E+02	2.417	2.61E+02	0.00E+00
D04	SYBR		Unkn	Female Abdomen	29.79	29.79	0.000	3.837E+02	2.584	3.84E+02	0.00E+00
D05	SYBR		Unkn	0h/1h cleavage	30.09	30.09	0.000	3.147E+02	2.498	3.15E+02	0.00E+00
D06	SYBR		Unkn	0h/4h cleavage	29.34	29.34	0.000	5.175E+02	2.714	5.18E+02	0.00E+00
E01	SYBR		Unkn	0h/8h cleavage	30.67	30.67	0.000	2.156E+02	2.334	2.16E+02	0.00E+00
E02	SYBR		Unkn	0h/12h morula	30.00	30.00	0.000	3.339E+02	2.524	3.34E+02	0.00E+00
E03	SYBR		Unkn	4h/12h morula	30.40	30.40	0.000	2.580E+02	2.412	2.58E+02	0.00E+00
E04	SYBR		Unkn	8h/16h morula	30.11	30.11	0.000	3.119E+02	2.494	3.12E+02	0.00E+00
E05	SYBR		Unkn	16h/24h morula	29.08	29.08	0.000	6.143E+02	2.788	6.14E+02	0.00E+00
F01	SYBR		Unkn	Male Head	28.32	28.32	0.000	1.012E+03	3.005	1.01E+03	0.00E+00
F02	SYBR		Unkn	Male Abdomen	29.50	29.50	0.000	4.654E+02	2.668	4.65E+02	0.00E+00
F03	SYBR		Unkn	Female Head	30.56	30.56	0.000	2.309E+02	2.363	2.31E+02	0.00E+00
F04	SYBR		Unkn	Female Abdomen	29.82	29.82	0.000	3.760E+02	2.575	3.76E+02	0.00E+00
F05	SYBR		Unkn	0h/1h cleavage	28.14	28.14	0.000	1.141E+03	3.057	1.14E+03	0.00E+00
F06	SYBR		Unkn	0h/4h cleavage	30.07	30.07	0.000	3.190E+02	2.504	3.19E+02	0.00E+00
G01	SYBR		Unkn	0h/8h cleavage	29.10	29.10	0.000	6.039E+02	2.781	6.04E+02	0.00E+00
G02	SYBR		Unkn	0h/12h morula	28.98	28.98	0.000	6.548E+02	2.816	6.55E+02	0.00E+00
G03	SYBR		Unkn	4h/12h morula	29.74	29.74	0.000	3.965E+02	2.598	3.97E+02	0.00E+00
G04	SYBR		Unkn	8h/16h morula	29.54	29.54	0.000	4.519E+02	2.655	4.52E+02	0.00E+00
G05	SYBR		Unkn	16h/24h morula	29.84	29.84	0.000	3.732E+02	2.572	3.73E+02	0.00E+00
H01	SYBR		Std	M2053rtF2rtR2	23.03	23.03	0.000	7.040E+04	4.848	7.04E+04	0.00E+00
H02	SYBR		Std	M2053rtF2rtR2	23.05	23.05	0.000	7.040E+04	4.848	7.04E+04	0.00E+00
H03	SYBR		Std	M2053rtF2rtR2	23.28	23.28	0.000	7.040E+04	4.848	7.04E+04	0.00E+00
H04	SYBR		Std	M2053rtF2rtR2	28.30	28.30	0.000	7.040E+02	2.848	7.04E+02	0.00E+00
H05	SYBR		Std	M2053rtF2rtR2	28.52	28.52	0.000	7.040E+02	2.848	7.04E+02	0.00E+00
H06	SYBR		Std	M2053rtF2rtR2	28.15	28.15	0.000	7.040E+02	2.848	7.04E+02	0.00E+00

Melt Curve

Step #: 7

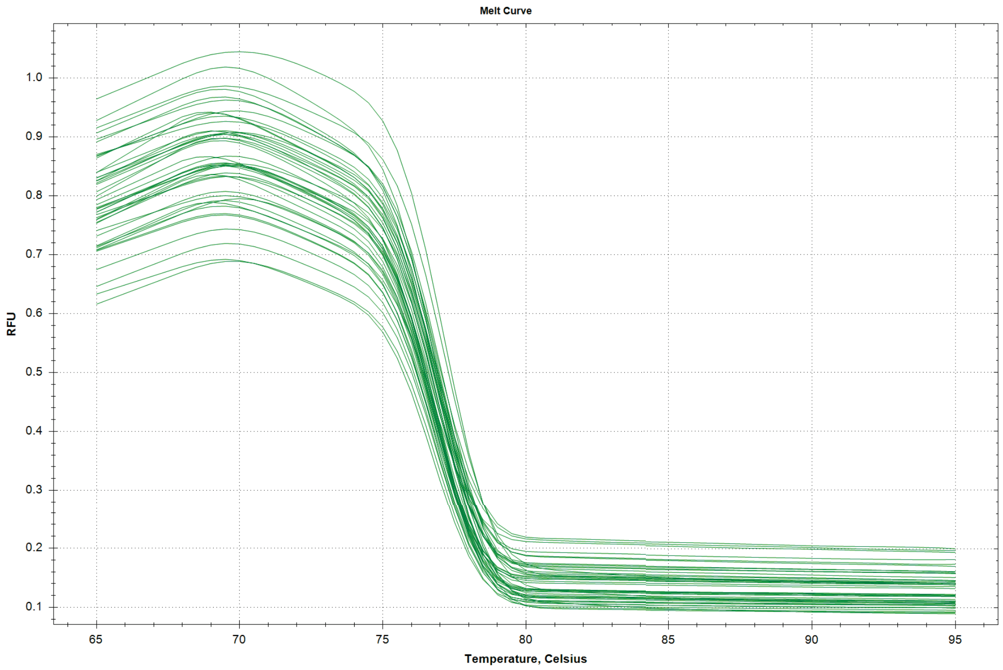

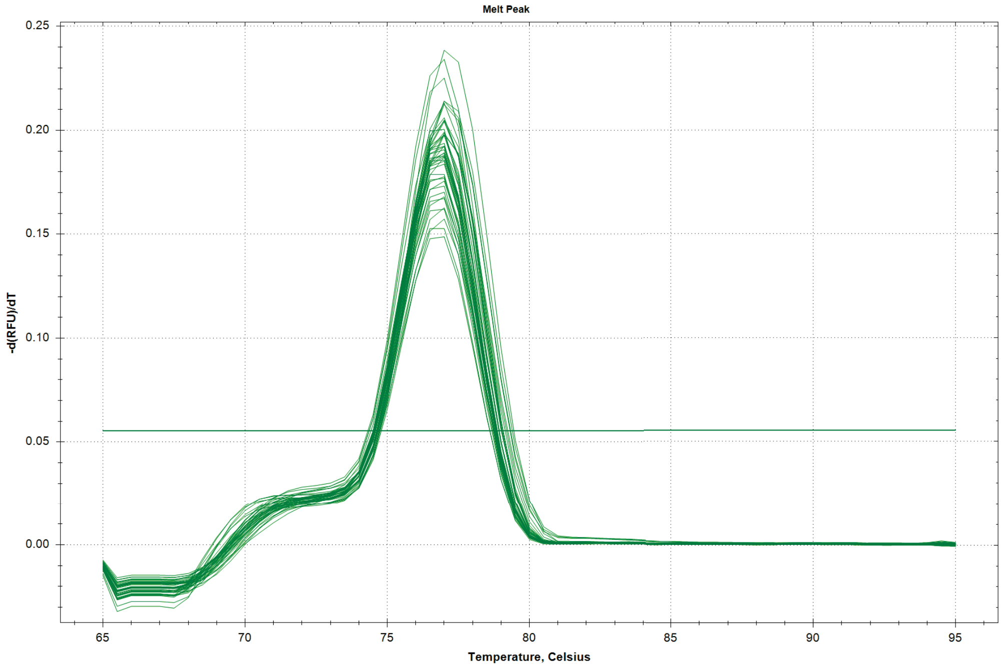

Melt Curve Data

Well	Fluor	Target	Content	Sample	Melt Temp
A01	SYBR		Std	M2053rtF2rtR2	77.00
A02	SYBR		Std	M2053rtF2rtR2	77.00
A03	SYBR		Std	M2053rtF2rtR2	77.00
A04	SYBR		Std	M2053rtF2rtR2	77.00

Melt Curve Data

Well	Fluor	Target	Content	Sample	Melt Temp
A05	SYBR		Std	M2053rtF2rtR2	77.00
A06	SYBR		Std	M2053rtF2rtR2	77.00
B01	SYBR		Unkn	Male Head	77.00
B02	SYBR		Unkn	Male Abdomen	77.00
B03	SYBR		Unkn	Female Head	77.00
B04	SYBR		Unkn	Female Abdomen	77.00
B05	SYBR		Unkn	0h/1h cleavage	77.00
B06	SYBR		Unkn	0h/4h cleavage	77.00
C01	SYBR		Unkn	0h/8h cleavage	77.00
C02	SYBR		Unkn	0h/12h morula	77.00
C03	SYBR		Unkn	4h/12h morula	77.00
C04	SYBR		Unkn	8h/16h morula	77.00
C05	SYBR		Unkn	16h/24h morula	77.00
D01	SYBR		Unkn	Male Head	77.00
D02	SYBR		Unkn	Male Abdomen	77.00
D03	SYBR		Unkn	Female Head	77.00
D04	SYBR		Unkn	Female Abdomen	77.00
D05	SYBR		Unkn	0h/1h cleavage	77.00
D06	SYBR		Unkn	0h/4h cleavage	77.00
E01	SYBR		Unkn	0h/8h cleavage	77.00
E02	SYBR		Unkn	0h/12h morula	77.00
E03	SYBR		Unkn	4h/12h morula	77.00
E04	SYBR		Unkn	8h/16h morula	77.00
E05	SYBR		Unkn	16h/24h morula	77.00
F01	SYBR		Unkn	Male Head	77.00
F02	SYBR		Unkn	Male Abdomen	77.00
F03	SYBR		Unkn	Female Head	77.00
F04	SYBR		Unkn	Female Abdomen	77.00
F05	SYBR		Unkn	0h/1h cleavage	77.00
F06	SYBR		Unkn	0h/4h cleavage	77.00
G01	SYBR		Unkn	0h/8h cleavage	77.00
G02	SYBR		Unkn	0h/12h morula	77.00
G03	SYBR		Unkn	4h/12h morula	77.00
G04	SYBR		Unkn	8h/16h morula	77.00
G05	SYBR		Unkn	16h/24h morula	77.00
H01	SYBR		Std	M2053rtF2rtR2	77.00
H02	SYBR		Std	M2053rtF2rtR2	77.00
H03	SYBR		Std	M2053rtF2rtR2	77.00
H04	SYBR		Std	M2053rtF2rtR2	77.00
H05	SYBR		Std	M2053rtF2rtR2	77.00
H06	SYBR		Std	M2053rtF2rtR2	77.00

End Point

Fluorophore/Target: SYBR

End Cycles to Average: 5
Mode: Percentage of Range - 10
Lowest RFU Value: 0.302
Highest RFU Value: 0.637
Cut Off Value:

SYBR End Point Data

SYBR End Point Data

Well	Fluor	Content	Sample	End RFU	Call
A01	SYBR	Std	M2053rtF2rtR2	0.631	
A02	SYBR	Std	M2053rtF2rtR2	0.621	
A03	SYBR	Std	M2053rtF2rtR2	0.571	
A04	SYBR	Std	M2053rtF2rtR2	0.529	
A05	SYBR	Std	M2053rtF2rtR2	0.575	
A06	SYBR	Std	M2053rtF2rtR2	0.637	
B01	SYBR	Unkn	Male Head	0.479	
B02	SYBR	Unkn	Male Abdomen	0.413	
B03	SYBR	Unkn	Female Head	0.338	
B04	SYBR	Unkn	Female Abdomen	0.353	
B05	SYBR	Unkn	0h/1h cleavage	0.404	
B06	SYBR	Unkn	0h/4h cleavage	0.429	
C01	SYBR	Unkn	0h/8h cleavage	0.418	
C02	SYBR	Unkn	0h/12h morula	0.410	
C03	SYBR	Unkn	4h/12h morula	0.358	
C04	SYBR	Unkn	8h/16h morula	0.372	
C05	SYBR	Unkn	16h/24h morula	0.392	
D01	SYBR	Unkn	Male Head	0.387	
D02	SYBR	Unkn	Male Abdomen	0.384	
D03	SYBR	Unkn	Female Head	0.349	
D04	SYBR	Unkn	Female Abdomen	0.373	
D05	SYBR	Unkn	0h/1h cleavage	0.407	
D06	SYBR	Unkn	0h/4h cleavage	0.431	
E01	SYBR	Unkn	0h/8h cleavage	0.302	
E02	SYBR	Unkn	0h/12h morula	0.395	
E03	SYBR	Unkn	4h/12h morula	0.343	
E04	SYBR	Unkn	8h/16h morula	0.378	
E05	SYBR	Unkn	16h/24h morula	0.409	
F01	SYBR	Unkn	Male Head	0.431	
F02	SYBR	Unkn	Male Abdomen	0.412	
F03	SYBR	Unkn	Female Head	0.324	
F04	SYBR	Unkn	Female Abdomen	0.410	
F05	SYBR	Unkn	0h/1h cleavage	0.422	
F06	SYBR	Unkn	0h/4h cleavage	0.372	
G01	SYBR	Unkn	0h/8h cleavage	0.447	
G02	SYBR	Unkn	0h/12h morula	0.424	
G03	SYBR	Unkn	4h/12h morula	0.386	
G04	SYBR	Unkn	8h/16h morula	0.441	
G05	SYBR	Unkn	16h/24h morula	0.400	
H01	SYBR	Std	M2053rtF2rtR2	0.539	
H02	SYBR	Std	M2053rtF2rtR2	0.539	
H03	SYBR	Std	M2053rtF2rtR2	0.519	
H04	SYBR	Std	M2053rtF2rtR2	0.533	
H05	SYBR	Std	M2053rtF2rtR2	0.540	
H06	SYBR	Std	M2053rtF2rtR2	0.492	

QC Parameters

Data

Description	Value	Use	Results	Exclude Wells	All excluded wells
Negative control with a Cq less than	38	True		False	
NTC with a Cq less than	38	True		False	
NRT with a Cq less than	38	True		False	
Positive control with a Cq greater than	30	True		False	
Unknown without a Cq	N/A	True		False	
Standard without a Cq	N/A	True		False	
Efficiency greater than	110.0	True			
Efficiency less than	90.0	True			
Std Curve R^2 less than	0.980	True			
Replicate group Cq Std Dev greater than	0.20	True		False	

Cfmemo-1 (M4902)

Run Setup

Run Information

Run Date: 2014/08/15 14:03
Run User: admin
Run Type: User-defined
Plate File: WR Plate_48 wells_SYBR_test.pltd
ID:
Notes:
Sample Volume: 10
Temperature Control Mode: Calculated
Lid Temperature: 100
Base Serial Number: MM010818
Optical Head Serial Number: MO005400

Protocol

1: 95.0°C for 1:00
2: 95.0°C for 0:15
3: 60.0°C for 0:30
    Plate Read
4: GOTO 2, 39 more times
5: 95.0°C for 0:15
6: Melt Curve 65.0°C to 95.0°C: Increment 0.5°C 0:05
Plate Read

Plate Display

	1	2	3	4	5	6
A	Std-4<br>SYBR	Std-4<br>SYBR	Std-4<br>SYBR	Std-3<br>SYBR	Std-3<br>SYBR	Std-3<br>SYBR
B		Unk<br>SYBR<br>16h/24h morula	Unk<br>SYBR<br>8h/16h morula	Unk<br>SYBR<br>4h/12h morula	Unk<br>SYBR<br>0h/12h morula	Unk<br>SYBR<br>0h/8h cleavage
C	Unk<br>SYBR<br>0h/4h cleavage	Unk<br>SYBR<br>0h/1h cleavage	Unk<br>SYBR<br>Female Abdomen	Unk<br>SYBR<br>Female Head	Unk<br>SYBR<br>Male Abdomen	Unk<br>SYBR<br>Male Head
D		Unk<br>SYBR<br>16h/24h morula	Unk<br>SYBR<br>8h/16h morula	Unk<br>SYBR<br>4h/12h morula	Unk<br>SYBR<br>0h/12h morula	Unk<br>SYBR<br>0h/8h cleavage
E	Unk<br>SYBR<br>0h/4h cleavage	Unk<br>SYBR<br>0h/1h cleavage	Unk<br>SYBR<br>Female Abdomen	Unk<br>SYBR<br>Female Head	Unk<br>SYBR<br>Male Abdomen	Unk<br>SYBR<br>Male Head
F		Unk<br>SYBR<br>16h/24h morula	Unk<br>SYBR<br>8h/16h morula	Unk<br>SYBR<br>4h/12h morula	Unk<br>SYBR<br>0h/12h morula	Unk<br>SYBR<br>0h/8h cleavage
G	Unk<br>SYBR<br>0h/4h cleavage	Unk<br>SYBR<br>0h/1h cleavage	Unk<br>SYBR<br>Female Abdomen	Unk<br>SYBR<br>Female Head	Unk<br>SYBR<br>Male Abdomen	Unk<br>SYBR<br>Male Head
H	Std-2<br>SYBR	Std-2<br>SYBR	Std-2<br>SYBR	Std-1<br>SYBR	Std-1<br>SYBR	Std-1<br>SYBR

Quantification

Step #: 3
Analysis Mode: Fluorophore
Cq Determination: Single Threshold
Baseline Method:
SYBR: Auto Calculated
Threshold Setting:
SYBR: .12, Auto Calculated

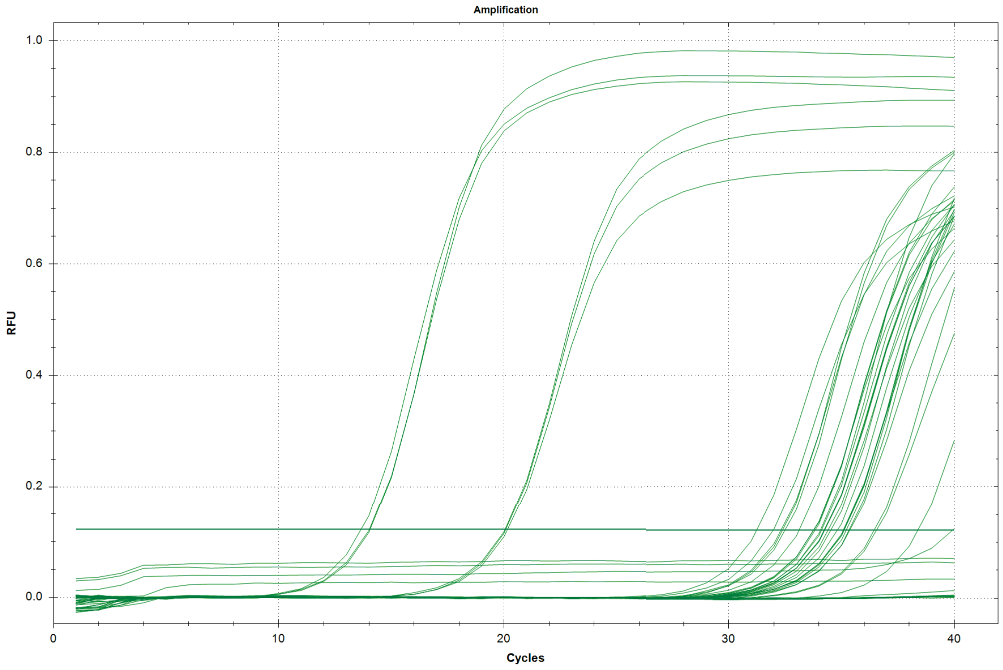

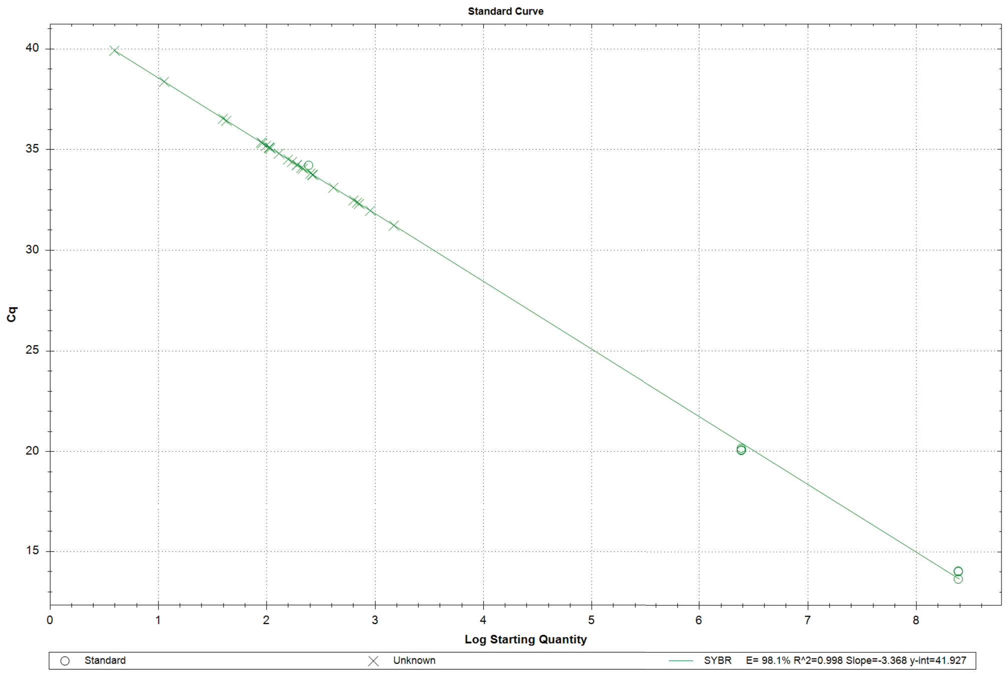

Quantification Data

Well	Fluor	Target	Content	Sample	Cq	Cq Mean	Cq Std. Dev	Starting Quantity (SQ)	Log Starting Quantity	SQ Mean	SQ Std. Dev
A01	SYBR		Std-4		N/A	0.00	0.000	2.430E+02	2.386	0.00E+00	0.00E+00

Quantification Data

Well	Fluor	Target	Content	Sample	Cq	Cq Mean	Cq Std. Dev	Starting Quantity (SQ)	Log Starting Quantity	SQ Mean	SQ Std. Dev
A02	SYBR		Std-4		34.23	34.23	0.000	2.430E+02	2.386	2.43E+02	0.00E+00
A03	SYBR		Std-4		N/A	0.00	0.000	2.430E+02	2.386	0.00E+00	0.00E+00
A04	SYBR		Std-3		N/A	0.00	0.000	2.430E+04	4.386	0.00E+00	0.00E+00
A05	SYBR		Std-3		N/A	0.00	0.000	2.430E+04	4.386	0.00E+00	0.00E+00
A06	SYBR		Std-3		N/A	0.00	0.000	2.430E+04	4.386	0.00E+00	0.00E+00
B02	SYBR		Unkn	16h/24h morula	N/A	0.00	0.000	N/A	N/A	0.00E+00	0.00E+00
B03	SYBR		Unkn	8h/16h morula	36.55	36.55	0.000	3.958E+01	1.598	3.96E+01	0.00E+00
B04	SYBR		Unkn	4h/12h morula	34.52	34.52	0.000	1.580E+02	2.199	1.58E+02	0.00E+00
B05	SYBR		Unkn	0h/12h morula	38.38	38.38	0.000	1.131E+01	1.054	1.13E+01	0.00E+00
B06	SYBR		Unkn	0h/8h cleavage	N/A	0.00	0.000	N/A	N/A	0.00E+00	0.00E+00
C01	SYBR		Unkn	0h/4h cleavage	34.81	34.81	0.000	1.297E+02	2.113	1.30E+02	0.00E+00
C02	SYBR		Unkn	0h/1h cleavage	34.40	34.40	0.000	1.720E+02	2.236	1.72E+02	0.00E+00
C03	SYBR		Unkn	Female Abdomen	34.25	34.25	0.000	1.908E+02	2.281	1.91E+02	0.00E+00
C04	SYBR		Unkn	Female Head	33.75	33.75	0.000	2.673E+02	2.427	2.67E+02	0.00E+00
C05	SYBR		Unkn	Male Abdomen	32.38	32.38	0.000	6.837E+02	2.835	6.84E+02	0.00E+00
C06	SYBR		Unkn	Male Head	35.21	35.21	0.000	9.895E+01	1.995	9.90E+01	0.00E+00
D02	SYBR		Unkn	16h/24h morula	34.25	34.25	0.000	1.908E+02	2.281	1.91E+02	0.00E+00
D03	SYBR		Unkn	8h/16h morula	35.08	35.08	0.000	1.082E+02	2.034	1.08E+02	0.00E+00
D04	SYBR		Unkn	4h/12h morula	35.11	35.11	0.000	1.054E+02	2.023	1.05E+02	0.00E+00
D05	SYBR		Unkn	0h/12h morula	N/A	0.00	0.000	N/A	N/A	0.00E+00	0.00E+00
D06	SYBR		Unkn	0h/8h cleavage	35.10	35.10	0.000	1.061E+02	2.026	1.06E+02	0.00E+00
E01	SYBR		Unkn	0h/4h cleavage	31.97	31.97	0.000	9.030E+02	2.956	9.03E+02	0.00E+00
E02	SYBR		Unkn	0h/1h cleavage	33.12	33.12	0.000	4.130E+02	2.616	4.13E+02	0.00E+00
E03	SYBR		Unkn	Female Abdomen	31.24	31.24	0.000	1.494E+03	3.174	1.49E+03	0.00E+00
E04	SYBR		Unkn	Female Head	34.11	34.11	0.000	2.095E+02	2.321	2.09E+02	0.00E+00
E05	SYBR		Unkn	Male Abdomen	32.49	32.49	0.000	6.342E+02	2.802	6.34E+02	0.00E+00
E06	SYBR		Unkn	Male Head	32.32	32.32	0.000	7.145E+02	2.854	7.14E+02	0.00E+00
F02	SYBR		Unkn	16h/24h morula	N/A	0.00	0.000	N/A	N/A	0.00E+00	0.00E+00
F03	SYBR		Unkn	8h/16h morula	35.36	35.36	0.000	8.889E+01	1.949	8.89E+01	0.00E+00
F04	SYBR		Unkn	4h/12h morula	36.44	36.44	0.000	4.249E+01	1.628	4.25E+01	0.00E+00
F05	SYBR		Unkn	0h/12h morula	N/A	0.00	0.000	N/A	N/A	0.00E+00	0.00E+00

Quantification Data

Well	Fluor	Target	Content	Sample	Cq	Cq Mean	Cq Std. Dev	Starting Quantity (SQ)	Log Starting Quantity	SQ Mean	SQ Std. Dev
F06	SYBR		Unkn	0h/8h cleavage	33.76	33.76	0.000	2.652E+02	2.423	2.65E+02	0.00E+00
G01	SYBR		Unkn	0h/4h cleavage	34.06	34.06	0.000	2.163E+02	2.335	2.16E+02	0.00E+00
G02	SYBR		Unkn	0h/1h cleavage	35.33	35.33	0.000	9.113E+01	1.960	9.11E+01	0.00E+00
G03	SYBR		Unkn	Female Abdomen	N/A	0.00	0.000	N/A	N/A	0.00E+00	0.00E+00
G04	SYBR		Unkn	Female Head	39.92	39.92	0.000	3.936E+00	0.595	3.94E+00	0.00E+00
G05	SYBR		Unkn	Male Abdomen	33.83	33.83	0.000	2.544E+02	2.406	2.54E+02	0.00E+00
G06	SYBR		Unkn	Male Head	N/A	0.00	0.000	N/A	N/A	0.00E+00	0.00E+00
H01	SYBR		Std-2		20.04	20.09	0.055	2.430E+06	6.386	2.43E+06	0.00E+00
H02	SYBR		Std-2		20.07	20.09	0.055	2.430E+06	6.386	2.43E+06	0.00E+00
H03	SYBR		Std-2		20.15	20.09	0.055	2.430E+06	6.386	2.43E+06	0.00E+00
H04	SYBR		Std-1		14.02	13.91	0.224	2.430E+08	8.386	2.43E+08	0.00E+00
H05	SYBR		Std-1		14.05	13.91	0.224	2.430E+08	8.386	2.43E+08	0.00E+00
H06	SYBR		Std-1		13.65	13.91	0.224	2.430E+08	8.386	2.43E+08	0.00E+00

Melt Curve

Step #: 6

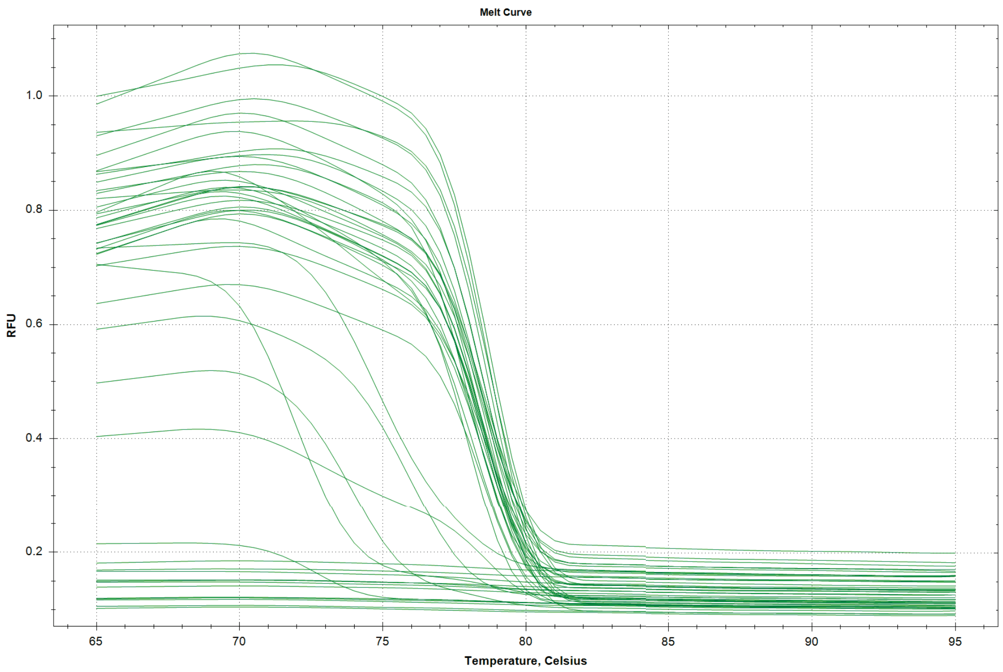

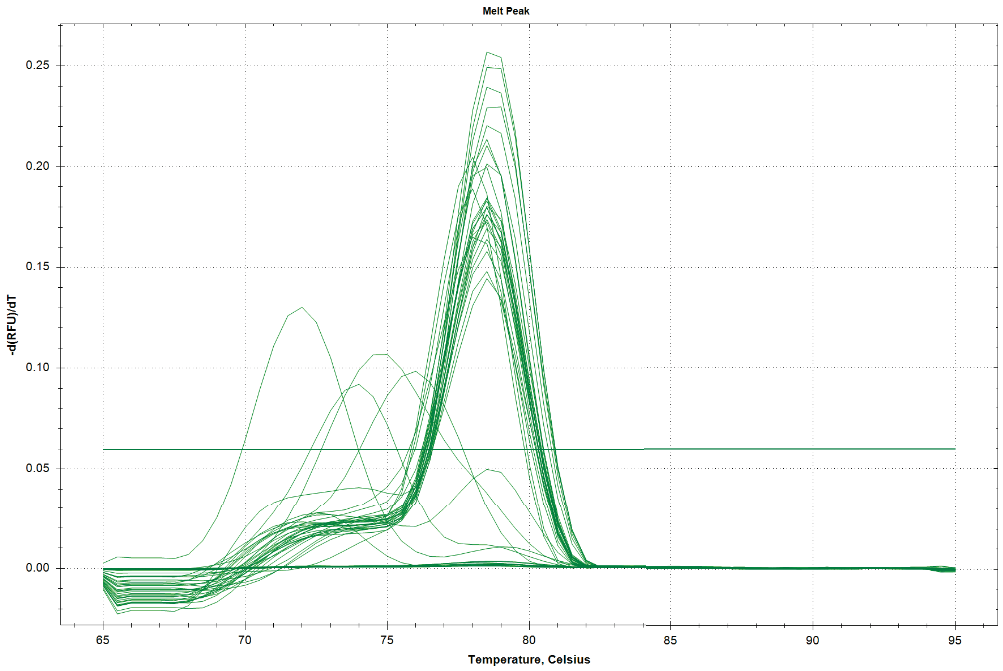

Melt Curve Data

Well	Fluor	Target	Content	Sample	Melt Temp
A01	SYBR		Std-4		None
A02	SYBR		Std-4		78.50
A03	SYBR		Std-4		None
A04	SYBR		Std-3		None
A05	SYBR		Std-3		None
A06	SYBR		Std-3		None
B02	SYBR		Unkn	16h/24h morula	None
B03	SYBR		Unkn	8h/16h morula	74.00
B04	SYBR		Unkn	4h/12h morula	78.50
B05	SYBR		Unkn	0h/12h morula	None
B06	SYBR		Unkn	0h/8h cleavage	None
C01	SYBR		Unkn	0h/4h cleavage	75.00
C02	SYBR		Unkn	0h/1h cleavage	78.50
C03	SYBR		Unkn	Female Abdomen	78.50
C04	SYBR		Unkn	Female Head	78.00
C05	SYBR		Unkn	Male Abdomen	78.50
C06	SYBR		Unkn	Male Head	78.50
D02	SYBR		Unkn	16h/24h morula	78.50
D03	SYBR		Unkn	8h/16h morula	78.50

Melt Curve Data

Well	Fluor	Target	Content	Sample	Melt Temp
D04	SYBR		Unkn	4h/12h morula	78.50
D05	SYBR		Unkn	0h/12h morula	None
D06	SYBR		Unkn	0h/8h cleavage	78.50
E01	SYBR		Unkn	0h/4h cleavage	78.50
E02	SYBR		Unkn	0h/1h cleavage	78.50
E03	SYBR		Unkn	Female Abdomen	78.50
E04	SYBR		Unkn	Female Head	78.50
E05	SYBR		Unkn	Male Abdomen	78.50
E06	SYBR		Unkn	Male Head	78.50
F02	SYBR		Unkn	16h/24h morula	None
F03	SYBR		Unkn	8h/16h morula	78.50
F04	SYBR		Unkn	4h/12h morula	76.00
F05	SYBR		Unkn	0h/12h morula	None
F06	SYBR		Unkn	0h/8h cleavage	78.00
G01	SYBR		Unkn	0h/4h cleavage	78.50
G02	SYBR		Unkn	0h/1h cleavage	72.00
G03	SYBR		Unkn	Female Abdomen	None
G04	SYBR		Unkn	Female Head	None
G05	SYBR		Unkn	Male Abdomen	78.00
G06	SYBR		Unkn	Male Head	None
H01	SYBR		Std-2		79.00
H02	SYBR		Std-2		78.50
H03	SYBR		Std-2		78.50
H04	SYBR		Std-1		78.50
H05	SYBR		Std-1		78.50
H06	SYBR		Std-1		78.50

End Point

Fluorophore/Target: SYBR

End Cycles to Average: 5
Mode: Percentage of Range - 10
Lowest RFU Value: 0.000408
Highest RFU Value: 0.973

Cut Off Value:

SYBR End Point Data

Well	Fluor	Content	Sample	End RFU	Call
A01	SYBR	Std-4		0.00283	
A02	SYBR	Std-4		0.529	
A03	SYBR	Std-4		0.00142	
A04	SYBR	Std-3		0.00159	
A05	SYBR	Std-3		0.00152	
A06	SYBR	Std-3		0.00875	
B02	SYBR	Unkn	16h/24h morula	0.0329	
B03	SYBR	Unkn	8h/16h morula	0.268	
B04	SYBR	Unkn	4h/12h morula	0.513	
B05	SYBR	Unkn	0h/12h morula	0.123	
B06	SYBR	Unkn	0h/8h cleavage	0.00232	
C01	SYBR	Unkn	0h/4h cleavage	0.479	
C02	SYBR	Unkn	0h/1h cleavage	0.491	
C03	SYBR	Unkn	Female Abdomen	0.527	
C04	SYBR	Unkn	Female Head	0.546	
C05	SYBR	Unkn	Male Abdomen	0.716	
C06	SYBR	Unkn	Male Head	0.465	
D02	SYBR	Unkn	16h/24h morula	0.537	
D03	SYBR	Unkn	8h/16h morula	0.435	
D04	SYBR	Unkn	4h/12h morula	0.461	
D05	SYBR	Unkn	0h/12h morula	0.00285	
D06	SYBR	Unkn	0h/8h cleavage	0.466	
E01	SYBR	Unkn	0h/4h cleavage	0.624	
E02	SYBR	Unkn	0h/1h cleavage	0.611	
E03	SYBR	Unkn	Female Abdomen	0.661	
E04	SYBR	Unkn	Female Head	0.551	
E05	SYBR	Unkn	Male Abdomen	0.708	
E06	SYBR	Unkn	Male Head	0.651	
F02	SYBR	Unkn	16h/24h morula	0.0707	
F03	SYBR	Unkn	8h/16h morula	0.391	

SYBR End Point Data

Well	Fluor	Content	Sample	End RFU	Call
F04	SYBR	Unkn	4h/12h morula	0.302	
F05	SYBR	Unkn	0h/12h morula	0.000408	
F06	SYBR	Unkn	0h/8h cleavage	0.581	
G01	SYBR	Unkn	0h/4h cleavage	0.610	
G02	SYBR	Unkn	0h/1h cleavage	0.440	
G03	SYBR	Unkn	Female Abdomen	0.0635	
G04	SYBR	Unkn	Female Head	0.0798	
G05	SYBR	Unkn	Male Abdomen	0.589	
G06	SYBR	Unkn	Male Head	0.00171	
H01	SYBR	Std-2		0.893	
H02	SYBR	Std-2		0.847	
H03	SYBR	Std-2		0.767	
H04	SYBR	Std-1		0.915	
H05	SYBR	Std-1		0.973	
H06	SYBR	Std-1		0.935	

QC Parameters

Data

Description	Value	Use	Results	Exclude Wells	All excluded wells
Negative control with a Cq less than	38	True		False	
NTC with a Cq less than	38	True		False	
NRT with a Cq less than	38	True		False	
Positive control with a Cq greater than	30	True		False	
Unknown without a Cq	N/A	True	SYBR:B2, B6, D5, F2, F5, G3, G6.	False	
Standard without a Cq	N/A	True	SYBR:A1, A3, A4, A5, A6.	False	
Efficiency greater than	110.0	True			
Efficiency less than	90.0	True			

Data

Description	Value	Use	Results	Exclude Wells	All excluded wells
Std Curve R^2 less than	0.980	True			
Replicate group Cq Std Dev greater than	0.20	True	SYBR:H4, H5, H6.	False	

CflncRNA-1

Run Setup

Run Information

Run Date: 2014/08/20 17:42
Run User: admin
Run Type: User-defined
Plate File: WR Plate_48 wells_SYBR_test.pltd
ID:
Notes:
Sample Volume: 10
Temperature Control Mode: Calculated
Lid Temperature: 100
Base Serial Number: MM010818
Optical Head Serial Number: MO005400

Protocol

1: 95.0°C for 1:00
2: 95.0°C for 0:15
3: 65.0°C for 0:15
4: 72.0°C for 0:30
Plate Read
5: GOTO 2, 39 more times
6: 95.0°C for 0:15
7: Melt Curve 65.0°C to 95.0°C: Increment 0.5°C 0:05
Plate Read

Plate Display

	1	2	3	4	5	6
A	Std-1<br>SYBR<br>lnc1rtF1rtR1	Std-1<br>SYBR<br>lnc1rtF1rtR1	Std-1<br>SYBR<br>lnc1rtF1rtR1	Std-2<br>SYBR<br>lnc1rtF1rtR1	Std-2<br>SYBR<br>lnc1rtF1rtR1	Std-2<br>SYBR<br>lnc1rtF1rtR1
B	Unk<br>SYBR<br>Male Head	Unk<br>SYBR<br>Male Abdomen	Unk<br>SYBR<br>Female Head	Unk<br>SYBR<br>Female Abdomen	Unk<br>SYBR<br>0h/1h cleavage	Unk<br>SYBR<br>0h/4h cleavage
C	Unk<br>SYBR<br>0h/8h cleavage	Unk<br>SYBR<br>0h/12h morula	Unk<br>SYBR<br>4h/12h morula	Unk<br>SYBR<br>8h/16h morula	Unk<br>SYBR<br>16h/24h morula	
D	Unk<br>SYBR<br>Male Head	Unk<br>SYBR<br>Male Abdomen	Unk<br>SYBR<br>Female Head	Unk<br>SYBR<br>Female Abdomen	Unk<br>SYBR<br>0h/1h cleavage	Unk<br>SYBR<br>0h/4h cleavage
E	Unk<br>SYBR<br>0h/8h cleavage	Unk<br>SYBR<br>0h/12h morula	Unk<br>SYBR<br>4h/12h morula	Unk<br>SYBR<br>8h/16h morula	Unk<br>SYBR<br>16h/24h morula	
F	Unk<br>SYBR<br>Male Head	Unk<br>SYBR<br>Male Abdomen	Unk<br>SYBR<br>Female Head	Unk<br>SYBR<br>Female Abdomen	Unk<br>SYBR<br>0h/1h cleavage	Unk<br>SYBR<br>0h/4h cleavage
G	Unk<br>SYBR<br>0h/8h cleavage	Unk<br>SYBR<br>0h/12h morula	Unk<br>SYBR<br>4h/12h morula	Unk<br>SYBR<br>8h/16h morula	Unk<br>SYBR<br>16h/24h morula	
H	Std-3<br>SYBR<br>lnc1rtF1rtR1	Std-3<br>SYBR<br>lnc1rtF1rtR1	Std-3<br>SYBR<br>lnc1rtF1rtR1	Std-4<br>SYBR<br>lnc1rtF1rtR1	Std-4<br>SYBR<br>lnc1rtF1rtR1	Std-4<br>SYBR<br>lnc1rtF1rtR1

Quantification

Step #: 4
Analysis Mode: Fluorophore
Cq Determination: Single Threshold
Baseline Method:
SYBR: Auto Calculated
Threshold Setting:
SYBR: .03, Auto Calculated

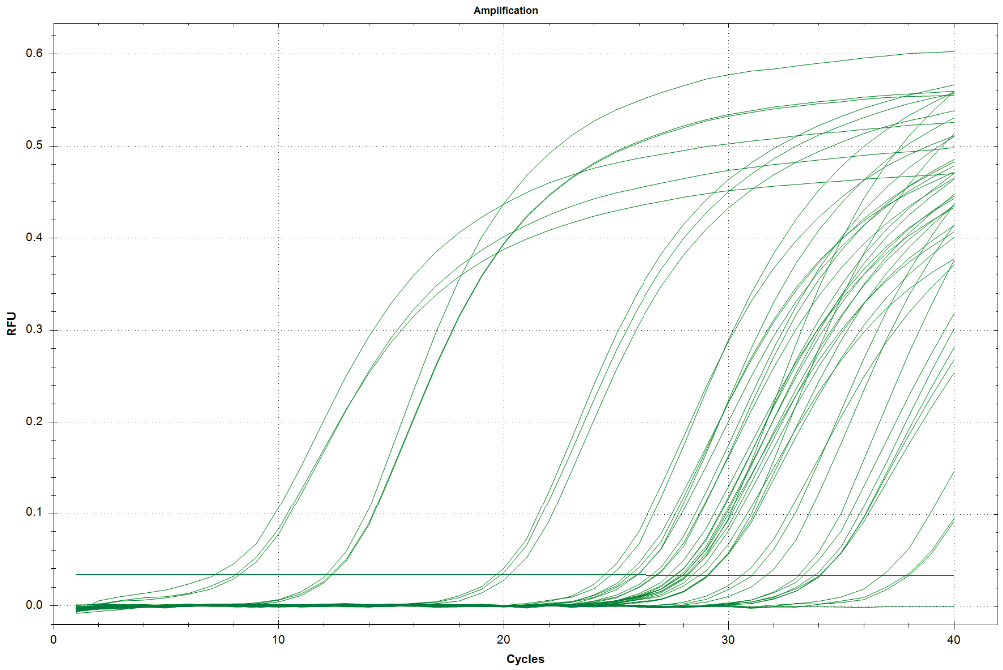

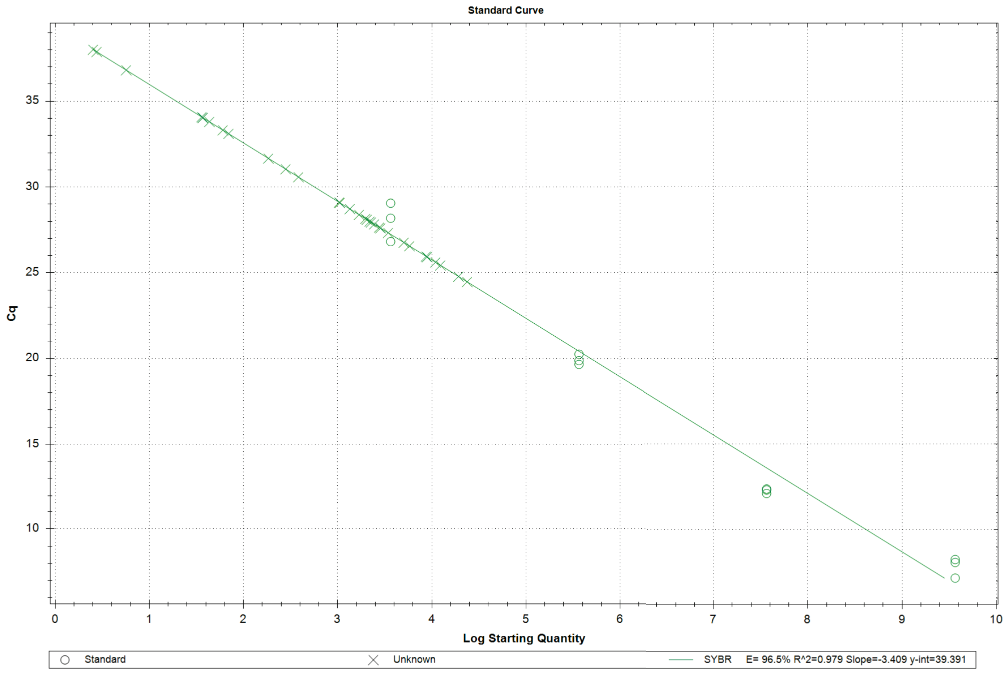

Quantification Data

Well	Fluor	Target	Content	Sample	Cq	Cq Mean	Cq Std. Dev	Starting Quantity (SQ)	Log Starting Quantity	SQ Mean	SQ Std. Dev
A01	SYBR		Std-1	lnc1rtF1rtR1	7.18	7.84	0.583	3.660E+09	9.563	3.66E+09	0.00E+00
A02	SYBR		Std-1	lnc1rtF1rtR1	8.26	7.84	0.583	3.660E+09	9.563	3.66E+09	0.00E+00
A03	SYBR		Std-1	lnc1rtF1rtR1	8.09	7.84	0.583	3.660E+09	9.563	3.66E+09	0.00E+00
A04	SYBR		Std-2	lnc1rtF1rtR1	12.32	12.27	0.138	3.660E+07	7.563	3.66E+07	0.00E+00
A05	SYBR		Std-2	lnc1rtF1rtR1	12.38	12.27	0.138	3.660E+07	7.563	3.66E+07	0.00E+00
A06	SYBR		Std-2	lnc1rtF1rtR1	12.11	12.27	0.138	3.660E+07	7.563	3.66E+07	0.00E+00
B01	SYBR		Unkn	Male Head	33.11	33.11	0.000	6.953E+01	1.842	6.95E+01	0.00E+00
B02	SYBR		Unkn	Male Abdomen	N/A	0.00	0.000	N/A	N/A	0.00E+00	0.00E+00
B03	SYBR		Unkn	Female Head	34.09	34.09	0.000	3.599E+01	1.556	3.60E+01	0.00E+00
B04	SYBR		Unkn	Female Abdomen	34.04	34.04	0.000	3.704E+01	1.569	3.70E+01	0.00E+00
B05	SYBR		Unkn	0h/1h cleavage	27.65	27.65	0.000	2.777E+03	3.444	2.78E+03	0.00E+00
B06	SYBR		Unkn	0h/4h cleavage	25.96	25.96	0.000	8.715E+03	3.940	8.72E+03	0.00E+00
C01	SYBR		Unkn	0h/8h cleavage	31.67	31.67	0.000	1.840E+02	2.265	1.84E+02	0.00E+00
C02	SYBR		Unkn	0h/12h morula	30.58	30.58	0.000	3.836E+02	2.584	3.84E+02	0.00E+00
C03	SYBR		Unkn	4h/12h morula	29.11	29.11	0.000	1.040E+03	3.017	1.04E+03	0.00E+00
C04	SYBR		Unkn	8h/16h morula	29.08	29.08	0.000	1.057E+03	3.024	1.06E+03	0.00E+00
C05	SYBR		Unkn	16h/24h morula	31.04	31.04	0.000	2.807E+02	2.448	2.81E+02	0.00E+00
D01	SYBR		Unkn	Male Head	38.01	38.01	0.000	2.534E+00	0.404	2.53E+00	0.00E+00
D02	SYBR		Unkn	Male Abdomen	36.82	36.82	0.000	5.681E+00	0.754	5.68E+00	0.00E+00
D03	SYBR		Unkn	Female Head	37.88	37.88	0.000	2.766E+00	0.442	2.77E+00	0.00E+00
D04	SYBR		Unkn	Female Abdomen	25.44	25.44	0.000	1.237E+04	4.092	1.24E+04	0.00E+00
D05	SYBR		Unkn	0h/1h cleavage	24.78	24.78	0.000	1.927E+04	4.285	1.93E+04	0.00E+00
D06	SYBR		Unkn	0h/4h cleavage	24.47	24.47	0.000	2.377E+04	4.376	2.38E+04	0.00E+00
E01	SYBR		Unkn	0h/8h cleavage	28.72	28.72	0.000	1.349E+03	3.130	1.35E+03	0.00E+00
E02	SYBR		Unkn	0h/12h morula	28.39	28.39	0.000	1.691E+03	3.228	1.69E+03	0.00E+00
E03	SYBR		Unkn	4h/12h morula	27.95	27.95	0.000	2.273E+03	3.357	2.27E+03	0.00E+00
E04	SYBR		Unkn	8h/16h morula	27.84	27.84	0.000	2.453E+03	3.390	2.45E+03	0.00E+00
E05	SYBR		Unkn	16h/24h morula	27.33	27.33	0.000	3.450E+03	3.538	3.45E+03	0.00E+00
F01	SYBR		Unkn	Male Head	33.32	33.32	0.000	6.049E+01	1.782	6.05E+01	0.00E+00
F02	SYBR		Unkn	Male Abdomen	33.81	33.81	0.000	4.348E+01	1.638	4.35E+01	0.00E+00
F03	SYBR		Unkn	Female Head	34.05	34.05	0.000	3.697E+01	1.568	3.70E+01	0.00E+00
F04	SYBR		Unkn	Female Abdomen	28.15	28.15	0.000	1.978E+03	3.296	1.98E+03	0.00E+00

Quantification Data

Well	Fluor	Target	Content	Sample	Cq	Cq Mean	Cq Std. Dev	Starting Quantity (SQ)	Log Starting Quantity	SQ Mean	SQ Std. Dev
F05	SYBR		Unkn	0h/1h cleavage	25.92	25.92	0.000	8.937E+03	3.951	8.94E+03	0.00E+00
F06	SYBR		Unkn	0h/4h cleavage	25.62	25.62	0.000	1.096E+04	4.040	1.10E+04	0.00E+00
G01	SYBR		Unkn	0h/8h cleavage	26.76	26.76	0.000	5.055E+03	3.704	5.06E+03	0.00E+00
G02	SYBR		Unkn	0h/12h morula	26.56	26.56	0.000	5.794E+03	3.763	5.79E+03	0.00E+00
G03	SYBR		Unkn	4h/12h morula	28.10	28.10	0.000	2.048E+03	3.311	2.05E+03	0.00E+00
G04	SYBR		Unkn	8h/16h morula	27.61	27.61	0.000	2.859E+03	3.456	2.86E+03	0.00E+00
G05	SYBR		Unkn	16h/24h morula	28.00	28.00	0.000	2.198E+03	3.342	2.20E+03	0.00E+00
H01	SYBR		Std-3	lnc1rtF1rtR1	19.66	19.93	0.301	3.660E+05	5.563	3.66E+05	0.00E+00
H02	SYBR		Std-3	lnc1rtF1rtR1	20.25	19.93	0.301	3.660E+05	5.563	3.66E+05	0.00E+00
H03	SYBR		Std-3	lnc1rtF1rtR1	19.88	19.93	0.301	3.660E+05	5.563	3.66E+05	0.00E+00
H04	SYBR		Std-4	lnc1rtF1rtR1	29.06	28.02	1.129	3.660E+03	3.563	3.66E+03	0.00E+00
H05	SYBR		Std-4	lnc1rtF1rtR1	28.18	28.02	1.129	3.660E+03	3.563	3.66E+03	0.00E+00
H06	SYBR		Std-4	lnc1rtF1rtR1	26.82	28.02	1.129	3.660E+03	3.563	3.66E+03	0.00E+00

Melt Curve

Step #: 7

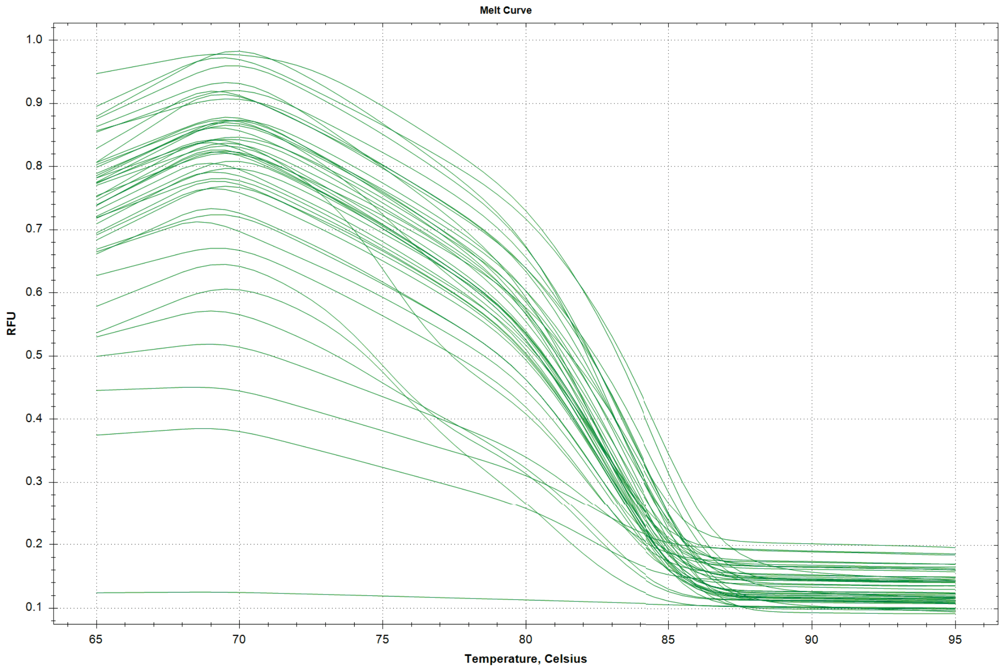

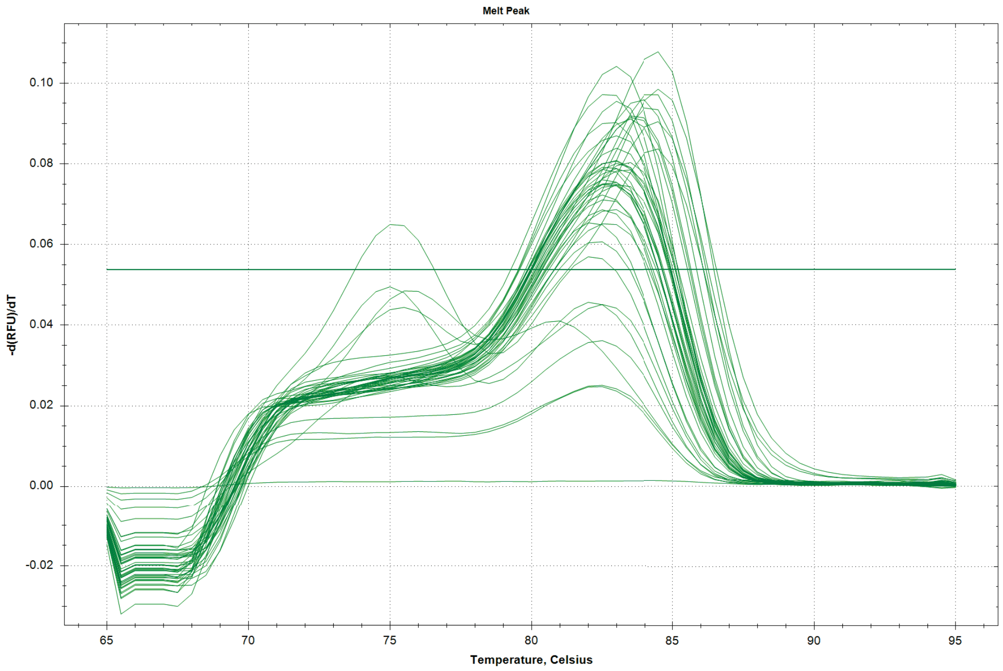

Melt Curve Data

Well	Fluor	Target	Content	Sample	Melt Temp
A01	SYBR		Std-1	lnc1rtF1rtR1	84.50
A02	SYBR		Std-1	lnc1rtF1rtR1	84.50
A03	SYBR		Std-1	lnc1rtF1rtR1	84.50
A04	SYBR		Std-2	lnc1rtF1rtR1	84.00
A05	SYBR		Std-2	lnc1rtF1rtR1	84.50
A06	SYBR		Std-2	lnc1rtF1rtR1	84.50
B01	SYBR		Unkn	Male Head	82.00
B01	SYBR		Unkn	Male Head	75.00
B02	SYBR		Unkn	Male Abdomen	None
B03	SYBR		Unkn	Female Head	None
B04	SYBR		Unkn	Female Abdomen	None
B05	SYBR		Unkn	0h/1h cleavage	83.00
B06	SYBR		Unkn	0h/4h cleavage	83.50
C01	SYBR		Unkn	0h/8h cleavage	82.50
C02	SYBR		Unkn	0h/12h morula	82.50
C03	SYBR		Unkn	4h/12h morula	83.00
C04	SYBR		Unkn	8h/16h morula	83.00
C05	SYBR		Unkn	16h/24h morula	82.50
D01	SYBR		Unkn	Male Head	None
D02	SYBR		Unkn	Male Abdomen	None
D03	SYBR		Unkn	Female Head	None

Melt Curve Data

Well	Fluor	Target	Content	Sample	Melt Temp
D04	SYBR		Unkn	Female Abdomen	83.50
D05	SYBR		Unkn	0h/1h cleavage	83.00
D06	SYBR		Unkn	0h/4h cleavage	83.00
E01	SYBR		Unkn	0h/8h cleavage	82.50
E02	SYBR		Unkn	0h/12h morula	82.50
E03	SYBR		Unkn	4h/12h morula	82.50
E04	SYBR		Unkn	8h/16h morula	82.50
E05	SYBR		Unkn	16h/24h morula	82.50
F01	SYBR		Unkn	Male Head	82.00
F02	SYBR		Unkn	Male Abdomen	82.50
F03	SYBR		Unkn	Female Head	None
F04	SYBR		Unkn	Female Abdomen	82.50
F05	SYBR		Unkn	0h/1h cleavage	83.00
F06	SYBR		Unkn	0h/4h cleavage	83.50
G01	SYBR		Unkn	0h/8h cleavage	83.00
G02	SYBR		Unkn	0h/12h morula	83.00
G03	SYBR		Unkn	4h/12h morula	83.00
G04	SYBR		Unkn	8h/16h morula	83.00
G05	SYBR		Unkn	16h/24h morula	83.00
H01	SYBR		Std-3	lnc1rtF1rtR1	84.00
H02	SYBR		Std-3	lnc1rtF1rtR1	83.50
H03	SYBR		Std-3	lnc1rtF1rtR1	83.50
H04	SYBR		Std-4	lnc1rtF1rtR1	83.00
H05	SYBR		Std-4	lnc1rtF1rtR1	82.50
H06	SYBR		Std-4	lnc1rtF1rtR1	83.00

End Point

Fluorophore/Target: SYBR

End Cycles to Average: 5
Mode: Percentage of Range - 10
Lowest RFU Value: -0.000451
Highest RFU Value: 0.600
Cut Off Value:

SYBR End Point Data

Well	Fluor	Content	Sample	End RFU	Call
A01	SYBR	Std-1	lnc1rtF1rtR1	0.522	
A02	SYBR	Std-1	lnc1rtF1rtR1	0.494	
A03	SYBR	Std-1	lnc1rtF1rtR1	0.467	
A04	SYBR	Std-2	lnc1rtF1rtR1	0.557	
A05	SYBR	Std-2	lnc1rtF1rtR1	0.553	
A06	SYBR	Std-2	lnc1rtF1rtR1	0.600	
B01	SYBR	Unkn	Male Head	0.271	
B02	SYBR	Unkn	Male Abdomen	-0.000451	
B03	SYBR	Unkn	Female Head	0.184	
B04	SYBR	Unkn	Female Abdomen	0.191	
B05	SYBR	Unkn	0h/1h cleavage	0.408	
B06	SYBR	Unkn	0h/4h cleavage	0.500	
C01	SYBR	Unkn	0h/8h cleavage	0.331	
C02	SYBR	Unkn	0h/12h morula	0.315	
C03	SYBR	Unkn	4h/12h morula	0.386	
C04	SYBR	Unkn	8h/16h morula	0.357	
C05	SYBR	Unkn	16h/24h morula	0.359	
D01	SYBR	Unkn	Male Head	0.0419	
D02	SYBR	Unkn	Male Abdomen	0.0739	
D03	SYBR	Unkn	Female Head	0.0446	
D04	SYBR	Unkn	Female Abdomen	0.454	
D05	SYBR	Unkn	0h/1h cleavage	0.531	
D06	SYBR	Unkn	0h/4h cleavage	0.489	
E01	SYBR	Unkn	0h/8h cleavage	0.341	
E02	SYBR	Unkn	0h/12h morula	0.375	
E03	SYBR	Unkn	4h/12h morula	0.371	
E04	SYBR	Unkn	8h/16h morula	0.407	
E05	SYBR	Unkn	16h/24h morula	0.400	
F01	SYBR	Unkn	Male Head	0.227	
F02	SYBR	Unkn	Male Abdomen	0.206	
F03	SYBR	Unkn	Female Head	0.175	
F04	SYBR	Unkn	Female Abdomen	0.402	
F05	SYBR	Unkn	0h/1h cleavage	0.449	

SYBR End Point Data

Well	Fluor	Content	Sample	End RFU	Call
F06	SYBR	Unkn	0h/4h cleavage	0.447	
G01	SYBR	Unkn	0h/8h cleavage	0.432	
G02	SYBR	Unkn	0h/12h morula	0.473	
G03	SYBR	Unkn	4h/12h morula	0.395	
G04	SYBR	Unkn	8h/16h morula	0.429	
G05	SYBR	Unkn	16h/24h morula	0.422	
H01	SYBR	Std-3	lnc1rtF1rtR1	0.555	
H02	SYBR	Std-3	lnc1rtF1rtR1	0.527	
H03	SYBR	Std-3	lnc1rtF1rtR1	0.545	
H04	SYBR	Std-4	lnc1rtF1rtR1	0.454	
H05	SYBR	Std-4	lnc1rtF1rtR1	0.506	
H06	SYBR	Std-4	lnc1rtF1rtR1	0.453	

QC Parameters

Data

Description	Value	Use	Results	Exclude Wells	All excluded wells
Negative control with a Cq less than	38	True		False	
NTC with a Cq less than	38	True		False	
NRT with a Cq less than	38	True		False	
Positive control with a Cq greater than	30	True		False	
Unknown without a Cq	N/A	True	SYBR:B2.	False	
Standard without a Cq	N/A	True		False	
Efficiency greater than	110.0	True			
Efficiency less than	90.0	True			
Std Curve R^2 less than	0.980	True	SYBR		
Replicate group Cq Std Dev greater than	0.20	True	SYBR:A1, A2, A3, H1, H2, H3, H4, H5, H6.	False	

CflncRNA-2

Run Setup

Run Information

Run Date: 2014/08/22 19:20
Run User: admin
Run Type: User-defined
Plate File: WR Plate_48 wells_SYBR_test.pltd
ID:
Notes:
Sample Volume: 10
Temperature Control Mode: Calculated
Lid Temperature: 100
Base Serial Number: MM010818
Optical Head Serial Number: MO005400

Protocol

1: 95.0°C for 1:00
2: 95.0°C for 0:15
3: 65.0°C for 0:15
4: 72.0°C for 0:30
Plate Read
5: GOTO 2, 39 more times
6: 95.0°C for 0:15
7: Melt Curve 65.0°C to 95.0°C: Increment 0.5°C 0:05
Plate Read

Plate Display

	1	2	3	4	5	6
A	Std-1<br>SYBR<br>lnc2rtF1R1	Std-1<br>SYBR<br>lnc2rtF1R1	Std-1<br>SYBR<br>lnc2rtF1R1	Std-2<br>SYBR<br>lnc2rtF1R1	Std-2<br>SYBR<br>lnc2rtF1R1	Std-2<br>SYBR<br>lnc2rtF1R1
B	Unk<br>SYBR<br>Male Head	Unk<br>SYBR<br>Male Abdomen	Unk<br>SYBR<br>Female Head	Unk<br>SYBR<br>Female Abdomen	Unk<br>SYBR<br>0h/1h cleavage	Unk<br>SYBR<br>0h/4h cleavage
C	Unk<br>SYBR<br>0h/8h cleavage	Unk<br>SYBR<br>0h/12h morula	Unk<br>SYBR<br>4h/12h morula	Unk<br>SYBR<br>8h/16h morula	Unk<br>SYBR<br>8h/24h morula	
D	Unk<br>SYBR<br>Male Head	Unk<br>SYBR<br>Male Abdomen	Unk<br>SYBR<br>Female Head	Unk<br>SYBR<br>Female Abdomen	Unk<br>SYBR<br>0h/1h cleavage	Unk<br>SYBR<br>0h/4h cleavage
E	Unk<br>SYBR<br>0h/8h cleavage	Unk<br>SYBR<br>0h/12h morula	Unk<br>SYBR<br>4h/12h morula	Unk<br>SYBR<br>8h/16h morula	Unk<br>SYBR<br>8h/24h morula	
F	Unk<br>SYBR<br>Male Head	Unk<br>SYBR<br>Male Abdomen	Unk<br>SYBR<br>Female Head	Unk<br>SYBR<br>Female Abdomen	Unk<br>SYBR<br>0h/1h cleavage	Unk<br>SYBR<br>0h/4h cleavage
G	Unk<br>SYBR<br>0h/8h cleavage	Unk<br>SYBR<br>0h/12h morula	Unk<br>SYBR<br>4h/12h morula	Unk<br>SYBR<br>8h/16h morula	Unk<br>SYBR<br>8h/24h morula	
H	Std-3<br>SYBR<br>lnc2rtF1R1	Std-3<br>SYBR<br>lnc2rtF1R1	Std-3<br>SYBR<br>lnc2rtF1R1	Std-4<br>SYBR<br>lnc2rtF1R1	Std-4<br>SYBR<br>lnc2rtF1R1	Std-4<br>SYBR<br>lnc2rtF1R1

Quantification

Step #: 4
Analysis Mode: Fluorophore
Cq Determination: Single Threshold
Baseline Method:
SYBR: Auto Calculated
Threshold Setting:
SYBR: .08, Auto Calculated

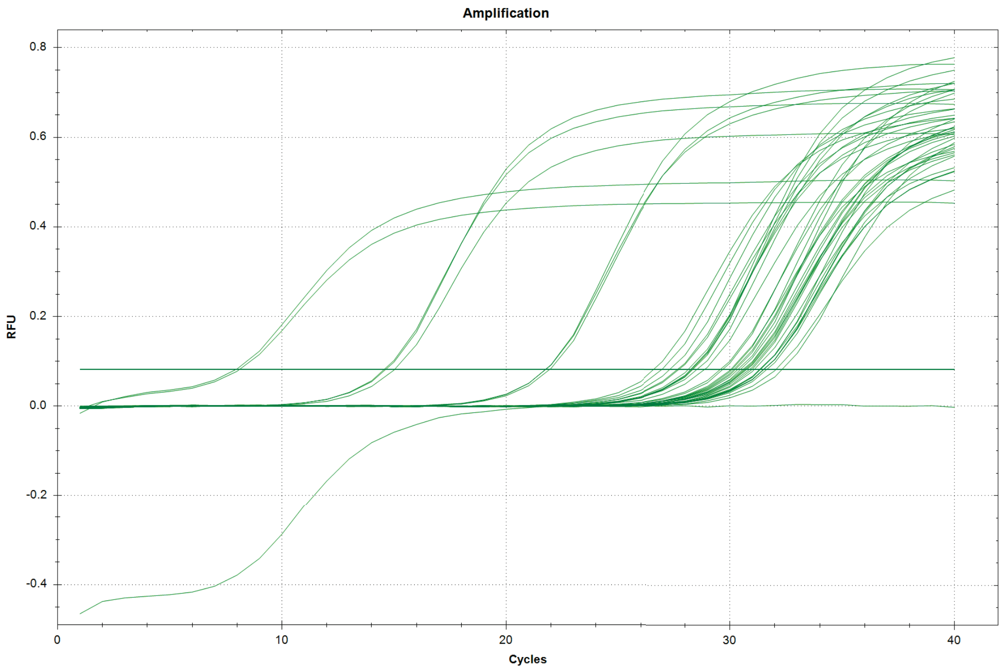

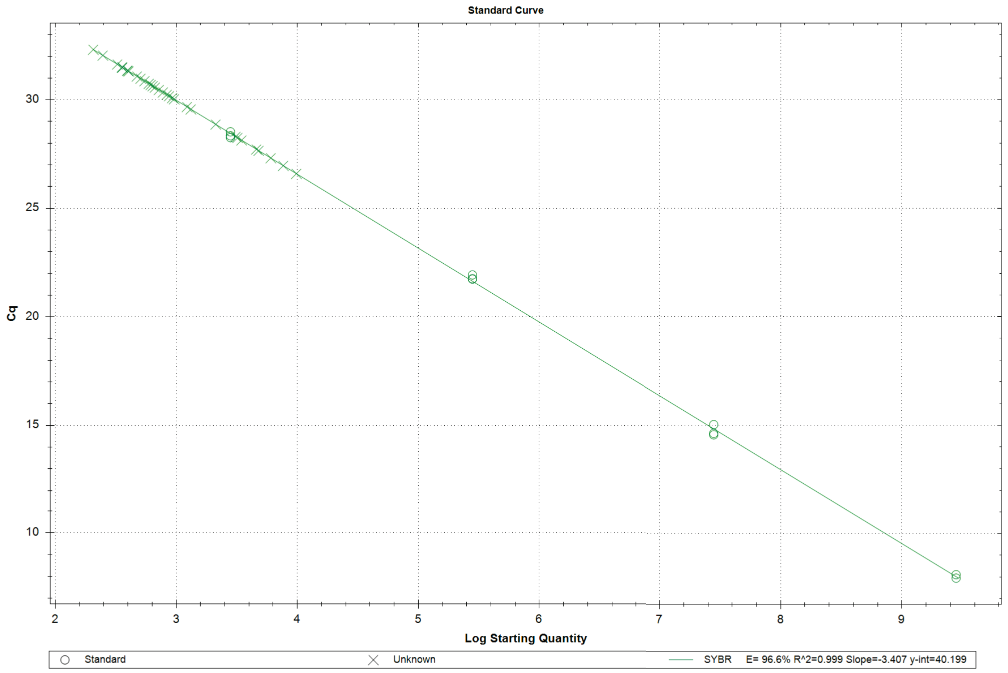

Quantification Data

Well	Fluor	Target	Content	Sample	Cq	Cq Mean	Cq Std. Dev	Starting Quantity (SQ)	Log Starting Quantity	SQ Mean	SQ Std. Dev
A01	SYBR		Std-1	lnc2rtF1R1	N/A	0.00	0.000	2.800E+09	9.447	0.00E+00	0.00E+00
A02	SYBR		Std-1	lnc2rtF1R1	7.95	8.03	0.112	2.800E+09	9.447	2.80E+09	0.00E+00
A03	SYBR		Std-1	lnc2rtF1R1	8.11	8.03	0.112	2.800E+09	9.447	2.80E+09	0.00E+00
A04	SYBR		Std-2	lnc2rtF1R1	15.02	14.73	0.253	2.800E+07	7.447	2.80E+07	0.00E+00
A05	SYBR		Std-2	lnc2rtF1R1	14.55	14.73	0.253	2.800E+07	7.447	2.80E+07	0.00E+00
A06	SYBR		Std-2	lnc2rtF1R1	14.62	14.73	0.253	2.800E+07	7.447	2.80E+07	0.00E+00
B01	SYBR		Unkn	Male Head	32.31	32.31	0.000	2.062E+02	2.314	2.06E+02	0.00E+00
B02	SYBR		Unkn	Male Abdomen	31.32	31.32	0.000	4.050E+02	2.608	4.05E+02	0.00E+00
B03	SYBR		Unkn	Female Head	32.05	32.05	0.000	2.471E+02	2.393	2.47E+02	0.00E+00
B04	SYBR		Unkn	Female Abdomen	31.50	31.50	0.000	3.567E+02	2.552	3.57E+02	0.00E+00
B05	SYBR		Unkn	0h/1h cleavage	30.04	30.04	0.000	9.619E+02	2.983	9.62E+02	0.00E+00
B06	SYBR		Unkn	0h/4h cleavage	28.25	28.25	0.000	3.211E+03	3.507	3.21E+03	0.00E+00
C01	SYBR		Unkn	0h/8h cleavage	30.46	30.46	0.000	7.208E+02	2.858	7.21E+02	0.00E+00
C02	SYBR		Unkn	0h/12h morula	31.08	31.08	0.000	4.737E+02	2.676	4.74E+02	0.00E+00
C03	SYBR		Unkn	4h/12h morula	30.75	30.75	0.000	5.947E+02	2.774	5.95E+02	0.00E+00
C04	SYBR		Unkn	8h/16h morula	30.64	30.64	0.000	6.401E+02	2.806	6.40E+02	0.00E+00
C05	SYBR		Unkn	8h/24h morula	31.35	31.35	0.000	3.949E+02	2.597	3.95E+02	0.00E+00
D01	SYBR		Unkn	Male Head	31.50	31.50	0.000	3.573E+02	2.553	3.57E+02	0.00E+00
D02	SYBR		Unkn	Male Abdomen	30.98	30.98	0.000	5.079E+02	2.706	5.08E+02	0.00E+00
D03	SYBR		Unkn	Female Head	31.49	31.49	0.000	3.599E+02	2.556	3.60E+02	0.00E+00
D04	SYBR		Unkn	Female Abdomen	27.66	27.66	0.000	4.800E+03	3.681	4.80E+03	0.00E+00
D05	SYBR		Unkn	0h/1h cleavage	26.97	26.97	0.000	7.658E+03	3.884	7.66E+03	0.00E+00
D06	SYBR		Unkn	0h/4h cleavage	26.60	26.60	0.000	9.830E+03	3.993	9.83E+03	0.00E+00
E01	SYBR		Unkn	0h/8h cleavage	31.34	31.34	0.000	3.982E+02	2.600	3.98E+02	0.00E+00
E02	SYBR		Unkn	0h/12h morula	30.87	30.87	0.000	5.480E+02	2.739	5.48E+02	0.00E+00
E03	SYBR		Unkn	4h/12h morula	30.69	30.69	0.000	6.165E+02	2.790	6.16E+02	0.00E+00
E04	SYBR		Unkn	8h/16h morula	30.24	30.24	0.000	8.377E+02	2.923	8.38E+02	0.00E+00
E05	SYBR		Unkn	8h/24h morula	28.87	28.87	0.000	2.118E+03	3.326	2.12E+03	0.00E+00
F01	SYBR		Unkn	Male Head	27.32	27.32	0.000	6.040E+03	3.781	6.04E+03	0.00E+00
F02	SYBR		Unkn	Male Abdomen	30.09	30.09	0.000	9.279E+02	2.968	9.28E+02	0.00E+00
F03	SYBR		Unkn	Female Head	31.64	31.64	0.000	3.260E+02	2.513	3.26E+02	0.00E+00

Quantification Data

Well	Fluor	Target	Content	Sample	Cq	Cq Mean	Cq Std. Dev	Starting Quantity (SQ)	Log Starting Quantity	SQ Mean	SQ Std. Dev
F04	SYBR		Unkn	Female Abdomen	29.57	29.57	0.000	1.321E+03	3.121	1.32E+03	0.00E+00
F05	SYBR		Unkn	0h/1h cleavage	27.72	27.72	0.000	4.596E+03	3.662	4.60E+03	0.00E+00
F06	SYBR		Unkn	0h/4h cleavage	28.14	28.14	0.000	3.468E+03	3.540	3.47E+03	0.00E+00
G01	SYBR		Unkn	0h/8h cleavage	28.30	28.30	0.000	3.101E+03	3.491	3.10E+03	0.00E+00
G02	SYBR		Unkn	0h/12h morula	29.68	29.68	0.000	1.227E+03	3.089	1.23E+03	0.00E+00
G03	SYBR		Unkn	4h/12h morula	30.18	30.18	0.000	8.754E+02	2.942	8.75E+02	0.00E+00
G04	SYBR		Unkn	8h/16h morula	30.35	30.35	0.000	7.783E+02	2.891	7.78E+02	0.00E+00
G05	SYBR		Unkn	8h/24h morula	30.58	30.58	0.000	6.684E+02	2.825	6.68E+02	0.00E+00
H01	SYBR		Std-3	lnc2rtF1R1	21.74	21.81	0.107	2.800E+05	5.447	2.80E+05	0.00E+00
H02	SYBR		Std-3	lnc2rtF1R1	21.93	21.81	0.107	2.800E+05	5.447	2.80E+05	0.00E+00
H03	SYBR		Std-3	lnc2rtF1R1	21.75	21.81	0.107	2.800E+05	5.447	2.80E+05	0.00E+00
H04	SYBR		Std-4	lnc2rtF1R1	28.26	28.38	0.137	2.800E+03	3.447	2.80E+03	0.00E+00
H05	SYBR		Std-4	lnc2rtF1R1	28.34	28.38	0.137	2.800E+03	3.447	2.80E+03	0.00E+00
H06	SYBR		Std-4	lnc2rtF1R1	28.53	28.38	0.137	2.800E+03	3.447	2.80E+03	0.00E+00

Melt Curve

Step #: 7

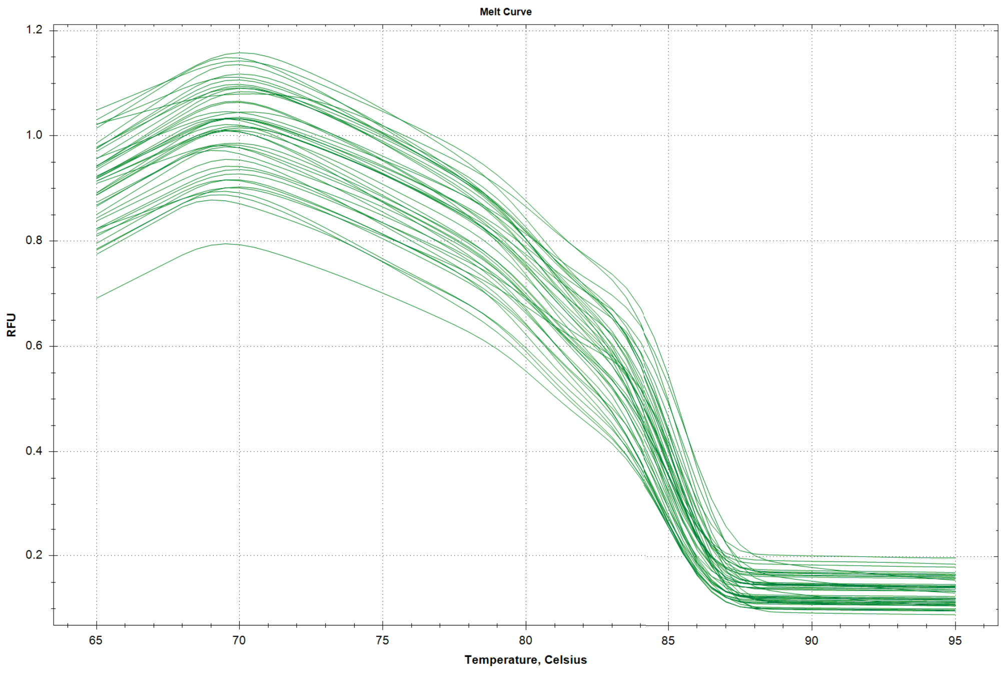

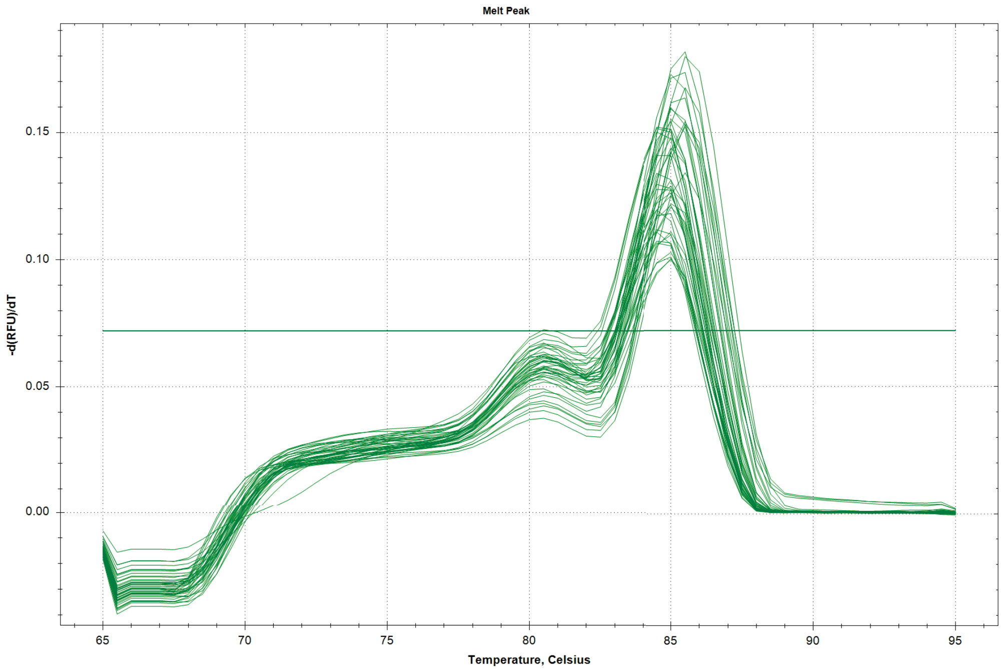

Melt Curve Data

Well	Fluor	Target	Content	Sample	Melt Temp
A01	SYBR		Std-1	lnc2rtF1R1	85.50
A02	SYBR		Std-1	lnc2rtF1R1	85.50
A03	SYBR		Std-1	lnc2rtF1R1	85.50
A04	SYBR		Std-2	lnc2rtF1R1	85.50
A05	SYBR		Std-2	lnc2rtF1R1	85.50
A06	SYBR		Std-2	lnc2rtF1R1	85.50
B01	SYBR		Unkn	Male Head	85.00
B02	SYBR		Unkn	Male Abdomen	85.00
B03	SYBR		Unkn	Female Head	85.00
B04	SYBR		Unkn	Female Abdomen	85.00
B05	SYBR		Unkn	0h/1h cleavage	85.00
B06	SYBR		Unkn	0h/4h cleavage	85.00
C01	SYBR		Unkn	0h/8h cleavage	85.00
C02	SYBR		Unkn	0h/12h morula	85.00
C03	SYBR		Unkn	4h/12h morula	84.50
C04	SYBR		Unkn	8h/16h morula	84.50
C05	SYBR		Unkn	8h/24h morula	85.00
D01	SYBR		Unkn	Male Head	85.00
D02	SYBR		Unkn	Male Abdomen	85.00
D03	SYBR		Unkn	Female Head	85.00

Melt Curve Data

Well	Fluor	Target	Content	Sample	Melt Temp
D04	SYBR		Unkn	Female Abdomen	85.00
D05	SYBR		Unkn	0h/1h cleavage	85.00
D06	SYBR		Unkn	0h/4h cleavage	85.00
E01	SYBR		Unkn	0h/8h cleavage	85.00
E02	SYBR		Unkn	0h/12h morula	84.50
E03	SYBR		Unkn	4h/12h morula	84.50
E04	SYBR		Unkn	8h/16h morula	84.50
E05	SYBR		Unkn	8h/24h morula	85.00
F01	SYBR		Unkn	Male Head	85.00
F02	SYBR		Unkn	Male Abdomen	85.00
F03	SYBR		Unkn	Female Head	85.00
F04	SYBR		Unkn	Female Abdomen	85.00
F05	SYBR		Unkn	0h/1h cleavage	84.50
F06	SYBR		Unkn	0h/4h cleavage	85.00
G01	SYBR		Unkn	0h/8h cleavage	84.50
G02	SYBR		Unkn	0h/12h morula	84.50
G03	SYBR		Unkn	4h/12h morula	84.50
G04	SYBR		Unkn	8h/16h morula	80.50
G04	SYBR		Unkn	8h/16h morula	84.50
G05	SYBR		Unkn	8h/24h morula	84.50
H01	SYBR		Std-3	lnc2rtF1R1	85.50
H02	SYBR		Std-3	lnc2rtF1R1	85.50
H03	SYBR		Std-3	lnc2rtF1R1	85.50
H04	SYBR		Std-4	lnc2rtF1R1	85.00
H05	SYBR		Std-4	lnc2rtF1R1	85.00
H06	SYBR		Std-4	lnc2rtF1R1	85.00

End Point

Fluorophore/Target: SYBR

End Cycles to Average: 5
Mode: Percentage of Range - 10
Lowest RFU Value: -0.000267
Highest RFU Value: 0.760
Cut Off Value:

SYBR End Point Data

Well	Fluor	Content	Sample	End RFU	Call
A01	SYBR	Std-1	lnc2rtF1R1	-0.000267	
A02	SYBR	Std-1	lnc2rtF1R1	0.504	
A03	SYBR	Std-1	lnc2rtF1R1	0.455	
A04	SYBR	Std-2	lnc2rtF1R1	0.608	
A05	SYBR	Std-2	lnc2rtF1R1	0.675	
A06	SYBR	Std-2	lnc2rtF1R1	0.707	
B01	SYBR	Unkn	Male Head	0.498	
B02	SYBR	Unkn	Male Abdomen	0.517	
B03	SYBR	Unkn	Female Head	0.426	
B04	SYBR	Unkn	Female Abdomen	0.473	
B05	SYBR	Unkn	0h/1h cleavage	0.537	
B06	SYBR	Unkn	0h/4h cleavage	0.640	
C01	SYBR	Unkn	0h/8h cleavage	0.558	
C02	SYBR	Unkn	0h/12h morula	0.487	
C03	SYBR	Unkn	4h/12h morula	0.536	
C04	SYBR	Unkn	8h/16h morula	0.524	
C05	SYBR	Unkn	8h/24h morula	0.573	
D01	SYBR	Unkn	Male Head	0.518	
D02	SYBR	Unkn	Male Abdomen	0.566	
D03	SYBR	Unkn	Female Head	0.530	
D04	SYBR	Unkn	Female Abdomen	0.629	
D05	SYBR	Unkn	0h/1h cleavage	0.648	
D06	SYBR	Unkn	0h/4h cleavage	0.629	
E01	SYBR	Unkn	0h/8h cleavage	0.473	
E02	SYBR	Unkn	0h/12h morula	0.567	
E03	SYBR	Unkn	4h/12h morula	0.518	
E04	SYBR	Unkn	8h/16h morula	0.567	
E05	SYBR	Unkn	8h/24h morula	0.586	
F01	SYBR	Unkn	Male Head	0.667	
F02	SYBR	Unkn	Male Abdomen	0.647	
F03	SYBR	Unkn	Female Head	0.496	
F04	SYBR	Unkn	Female Abdomen	0.600	

SYBR End Point Data

Well	Fluor	Content	Sample	End RFU	Call
F05	SYBR	Unkn	0h/1h cleavage	0.601	
F06	SYBR	Unkn	0h/4h cleavage	0.619	
G01	SYBR	Unkn	0h/8h cleavage	0.681	
G02	SYBR	Unkn	0h/12h morula	0.661	
G03	SYBR	Unkn	4h/12h morula	0.572	
G04	SYBR	Unkn	8h/16h morula	0.665	
G05	SYBR	Unkn	8h/24h morula	0.567	
H01	SYBR	Std-3	lnc2rtF1R1	0.760	
H02	SYBR	Std-3	lnc2rtF1R1	0.716	
H03	SYBR	Std-3	lnc2rtF1R1	0.700	
H04	SYBR	Std-4	lnc2rtF1R1	0.748	
H05	SYBR	Std-4	lnc2rtF1R1	0.720	
H06	SYBR	Std-4	lnc2rtF1R1	0.689	

QC Parameters

Data

Description	Value	Use	Results	Exclude Wells	All excluded wells
Negative control with a Cq less than	38	True		False	
NTC with a Cq less than	38	True		False	
NRT with a Cq less than	38	True		False	
Positive control with a Cq greater than	30	True		False	
Unknown without a Cq	N/A	True		False	
Standard without a Cq	N/A	True	SYBR:A1.	False	
Efficiency greater than	110.0	True			
Efficiency less than	90.0	True			
Std Curve R^2 less than	0.980	True			

Data

Description	Value	Use	Results	Exclude Wells	All excluded wells
Replicate group Cq Std Dev greater than	0.20	True	SYBR:A4, A5, A6.	False	

CflncRNA-3

Run Setup

Run Information

Run Date: 2014/08/21 18:30
Run User: admin
Run Type: User-defined
Plate File: WR Plate_48 wells_SYBR_test.pltd
ID:
Notes:
Sample Volume: 10
Temperature Control Mode: Calculated
Lid Temperature: 100
Base Serial Number: MM010818
Optical Head Serial Number: MO005400

Protocol

1: 95.0°C for 1:00
2: 95.0°C for 0:15
3: 65.0°C for 0:15
4: 72.0°C for 0:30
Plate Read
5: GOTO 2, 39 more times
6: 95.0°C for 0:15
7: Melt Curve 65.0°C to 95.0°C: Increment 0.5°C 0:05
Plate Read

Plate Display

	1	2	3	4	5	6
A	Std<br>SYBR<br>lnc3rtF1R1	Std<br>SYBR<br>lnc3rtF1R1	Std<br>SYBR<br>lnc3rtF1R1	Std<br>SYBR<br>lnc3rtF1R1	Std<br>SYBR<br>lnc3rtF1R1	Std<br>SYBR<br>lnc3rtF1R1
B	Unk<br>SYBR<br>Male Head 1st	Unk<br>SYBR<br>Male Abdomen	Unk<br>SYBR<br>Female Head	Unk<br>SYBR<br>Female Abdomen	Unk<br>SYBR<br>0h/1h cleavage	Unk<br>SYBR<br>0h/4h cleavage
C	Unk<br>SYBR<br>0h/8h cleavage	Unk<br>SYBR<br>0h/12h morula	Unk<br>SYBR<br>4h/12h morula	Unk<br>SYBR<br>8h/16h morula	Unk<br>SYBR<br>16h/24h morula	
D	Unk<br>SYBR<br>Male Head 2nd	Unk<br>SYBR<br>Male Abdomen	Unk<br>SYBR<br>Female Head	Unk<br>SYBR<br>Female Abdomen	Unk<br>SYBR<br>0h/1h cleavage	Unk<br>SYBR<br>0h/4h cleavage
E	Unk<br>SYBR<br>0h/8h cleavage	Unk<br>SYBR<br>0h/12h morula	Unk<br>SYBR<br>4h/12h morula	Unk<br>SYBR<br>8h/16h morula	Unk<br>SYBR<br>16h/24h morula	
F	Unk<br>SYBR<br>Male Head 3rd	Unk<br>SYBR<br>Male Abdomen	Unk<br>SYBR<br>Female Head	Unk<br>SYBR<br>Female Abdomen	Unk<br>SYBR<br>0h/1h cleavage	Unk<br>SYBR<br>0h/4h cleavage
G	Unk<br>SYBR<br>0h/8h cleavage	Unk<br>SYBR<br>0h/12h morula	Unk<br>SYBR<br>4h/12h morula	Unk<br>SYBR<br>8h/16h morula	Unk<br>SYBR<br>16h/24h morula	
H	Std<br>SYBR<br>lnc3rtF1R1	Std<br>SYBR<br>lnc3rtF1R1	Std<br>SYBR<br>lnc3rtF1R1	Std<br>SYBR<br>lnc3rtF1R1	Std<br>SYBR<br>lnc3rtF1R1	Std<br>SYBR<br>lnc3rtF1R1

Quantification

Step #: 4
Analysis Mode: Fluorophore
Cq Determination: Single Threshold
Baseline Method:
SYBR: Auto Calculated
Threshold Setting:
SYBR: .05, Auto Calculated

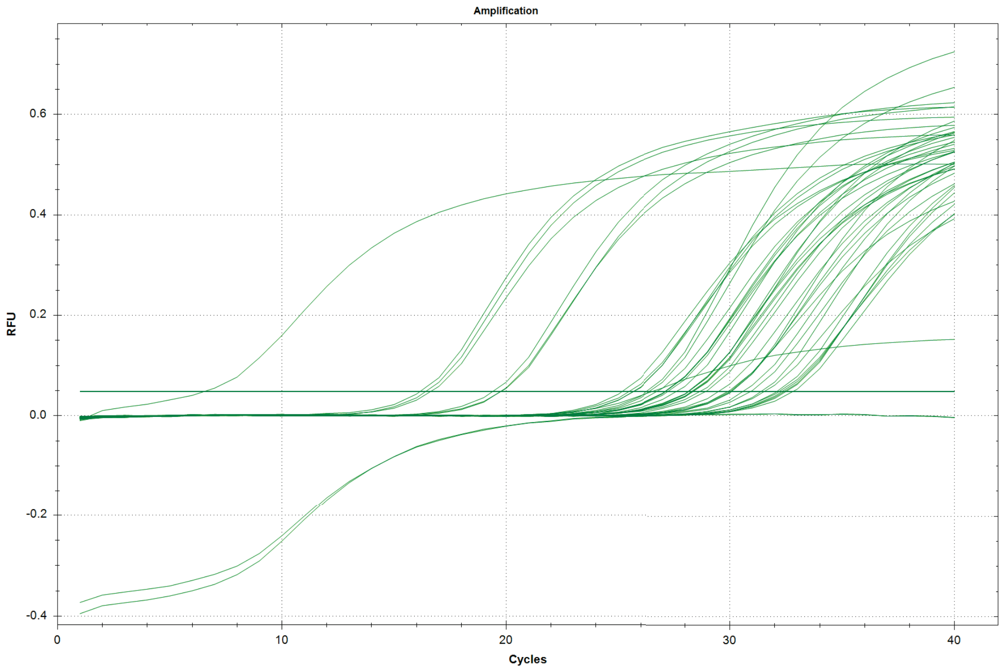

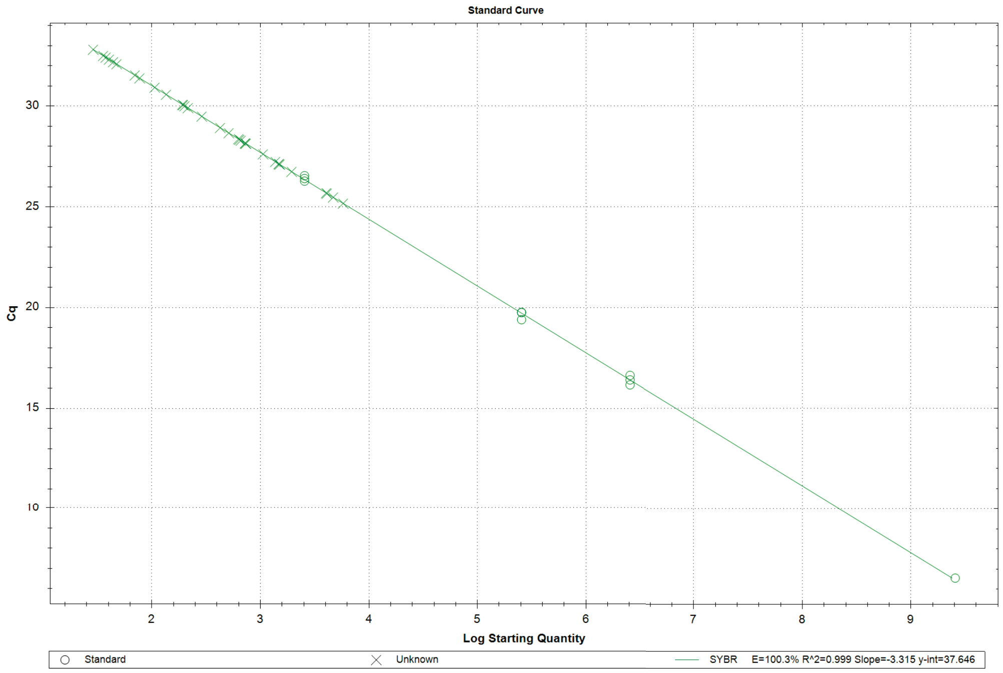

Quantification Data

Well	Fluor	Target	Content	Sample	Cq	Cq Mean	Cq Std. Dev	Starting Quantity (SQ)	Log Starting Quantity	SQ Mean	SQ Std. Dev
A01	SYBR		Std	lnc3rtF1R1	6.56	6.56	0.000	2.550E+09	9.407	2.55E+09	0.00E+00
A02	SYBR		Std	lnc3rtF1R1	N/A	0.00	0.000	2.550E+09	9.407	0.00E+00	0.00E+00
A03	SYBR		Std	lnc3rtF1R1	N/A	0.00	0.000	2.550E+09	9.407	0.00E+00	0.00E+00
A04	SYBR		Std	lnc3rtF1R1	16.41	16.41	0.000	2.550E+06	6.407	2.55E+06	0.00E+00
A05	SYBR		Std	lnc3rtF1R1	16.64	16.64	0.000	2.550E+06	6.407	2.55E+06	0.00E+00
A06	SYBR		Std	lnc3rtF1R1	16.16	16.16	0.000	2.550E+06	6.407	2.55E+06	0.00E+00
B01	SYBR		Unkn	Male Head 1st	31.39	31.39	0.000	7.714E+01	1.887	7.71E+01	0.00E+00
B02	SYBR		Unkn	Male Abdomen	32.50	32.50	0.000	3.564E+01	1.552	3.56E+01	0.00E+00
B03	SYBR		Unkn	Female Head	31.54	31.54	0.000	6.971E+01	1.843	6.97E+01	0.00E+00
B04	SYBR		Unkn	Female Abdomen	32.81	32.81	0.000	2.876E+01	1.459	2.88E+01	0.00E+00
B05	SYBR		Unkn	0h/1h cleavage	28.17	28.17	0.000	7.230E+02	2.859	7.23E+02	0.00E+00
B06	SYBR		Unkn	0h/4h cleavage	25.69	25.69	0.000	4.038E+03	3.606	4.04E+03	0.00E+00
C01	SYBR		Unkn	0h/8h cleavage	30.06	30.06	0.000	1.942E+02	2.288	1.94E+02	0.00E+00
C02	SYBR		Unkn	0h/12h morula	30.08	30.08	0.000	1.916E+02	2.282	1.92E+02	0.00E+00
C03	SYBR		Unkn	4h/12h morula	29.49	29.49	0.000	2.880E+02	2.459	2.88E+02	0.00E+00
C04	SYBR		Unkn	8h/16h morula	28.30	28.30	0.000	6.612E+02	2.820	6.61E+02	0.00E+00
C05	SYBR		Unkn	16h/24h morula	30.93	30.93	0.000	1.063E+02	2.026	1.06E+02	0.00E+00
D01	SYBR		Unkn	Male Head 2nd	32.09	32.09	0.000	4.735E+01	1.675	4.74E+01	0.00E+00
D02	SYBR		Unkn	Male Abdomen	32.42	32.42	0.000	3.768E+01	1.576	3.77E+01	0.00E+00
D03	SYBR		Unkn	Female Head	32.32	32.32	0.000	4.039E+01	1.606	4.04E+01	0.00E+00
D04	SYBR		Unkn	Female Abdomen	25.17	25.17	0.000	5.791E+03	3.763	5.79E+03	0.00E+00
D05	SYBR		Unkn	0h/1h cleavage	27.14	27.14	0.000	1.480E+03	3.170	1.48E+03	0.00E+00
D06	SYBR		Unkn	0h/4h cleavage	25.67	25.67	0.000	4.106E+03	3.613	4.11E+03	0.00E+00
E01	SYBR		Unkn	0h/8h cleavage	29.91	29.91	0.000	2.152E+02	2.333	2.15E+02	0.00E+00
E02	SYBR		Unkn	0h/12h morula	30.01	30.01	0.000	2.008E+02	2.303	2.01E+02	0.00E+00
E03	SYBR		Unkn	4h/12h morula	28.37	28.37	0.000	6.281E+02	2.798	6.28E+02	0.00E+00
E04	SYBR		Unkn	8h/16h morula	28.14	28.14	0.000	7.365E+02	2.867	7.37E+02	0.00E+00
E05	SYBR		Unkn	16h/24h morula	27.13	27.13	0.000	1.483E+03	3.171	1.48E+03	0.00E+00
F01	SYBR		Unkn	Male Head 3rd	28.93	28.93	0.000	4.262E+02	2.630	4.26E+02	0.00E+00
F02	SYBR		Unkn	Male Abdomen	30.58	30.58	0.000	1.355E+02	2.132	1.36E+02	0.00E+00

Quantification Data

Well	Fluor	Target	Content	Sample	Cq	Cq Mean	Cq Std. Dev	Starting Quantity (SQ)	Log Starting Quantity	SQ Mean	SQ Std. Dev
F03	SYBR		Unkn	Female Head	32.20	32.20	0.000	4.408E+01	1.644	4.41E+01	0.00E+00
F04	SYBR		Unkn	Female Abdomen	27.24	27.24	0.000	1.376E+03	3.138	1.38E+03	0.00E+00
F05	SYBR		Unkn	0h/1h cleavage	25.48	25.48	0.000	4.690E+03	3.671	4.69E+03	0.00E+00
F06	SYBR		Unkn	0h/4h cleavage	26.75	26.75	0.000	1.940E+03	3.288	1.94E+03	0.00E+00
G01	SYBR		Unkn	0h/8h cleavage	27.62	27.62	0.000	1.058E+03	3.024	1.06E+03	0.00E+00
G02	SYBR		Unkn	0h/12h morula	27.11	27.11	0.000	1.508E+03	3.178	1.51E+03	0.00E+00
G03	SYBR		Unkn	4h/12h morula	28.16	28.16	0.000	7.268E+02	2.861	7.27E+02	0.00E+00
G04	SYBR		Unkn	8h/16h morula	28.67	28.67	0.000	5.114E+02	2.709	5.11E+02	0.00E+00
G05	SYBR		Unkn	16h/24h morula	28.34	28.34	0.000	6.420E+02	2.808	6.42E+02	0.00E+00
H01	SYBR		Std	lnc3rtF1R1	19.40	19.40	0.000	2.550E+05	5.407	2.55E+05	0.00E+00
H02	SYBR		Std	lnc3rtF1R1	19.75	19.75	0.000	2.550E+05	5.407	2.55E+05	0.00E+00
H03	SYBR		Std	lnc3rtF1R1	19.77	19.77	0.000	2.550E+05	5.407	2.55E+05	0.00E+00
H04	SYBR		Std	lnc3rtF1R1	26.41	26.41	0.000	2.550E+03	3.407	2.55E+03	0.00E+00
H05	SYBR		Std	lnc3rtF1R1	26.28	26.28	0.000	2.550E+03	3.407	2.55E+03	0.00E+00
H06	SYBR		Std	lnc3rtF1R1	26.55	26.55	0.000	2.550E+03	3.407	2.55E+03	0.00E+00

Melt Curve

Step #: 7

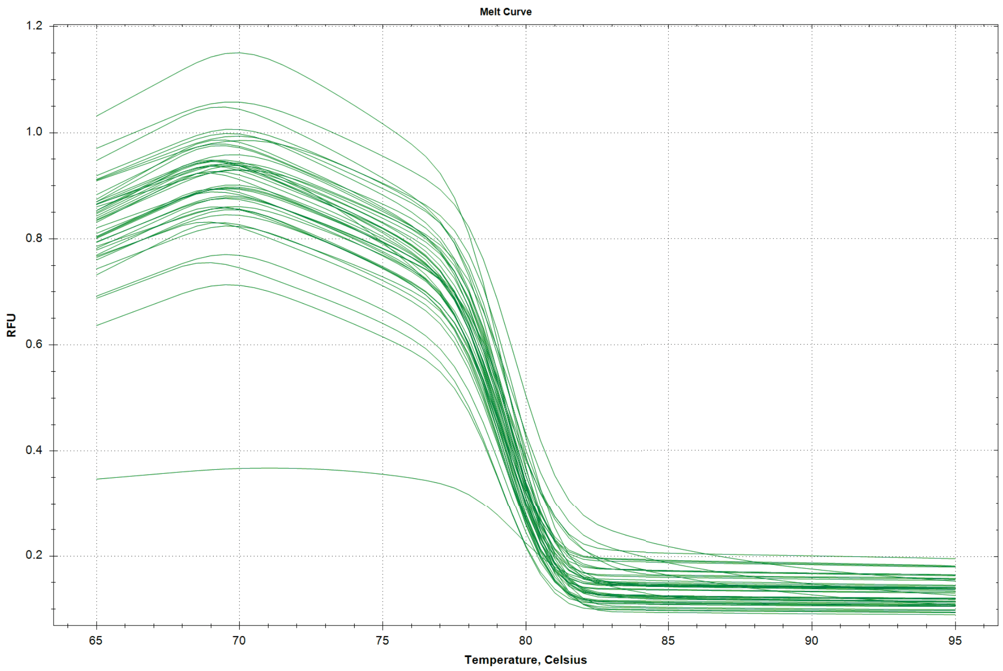

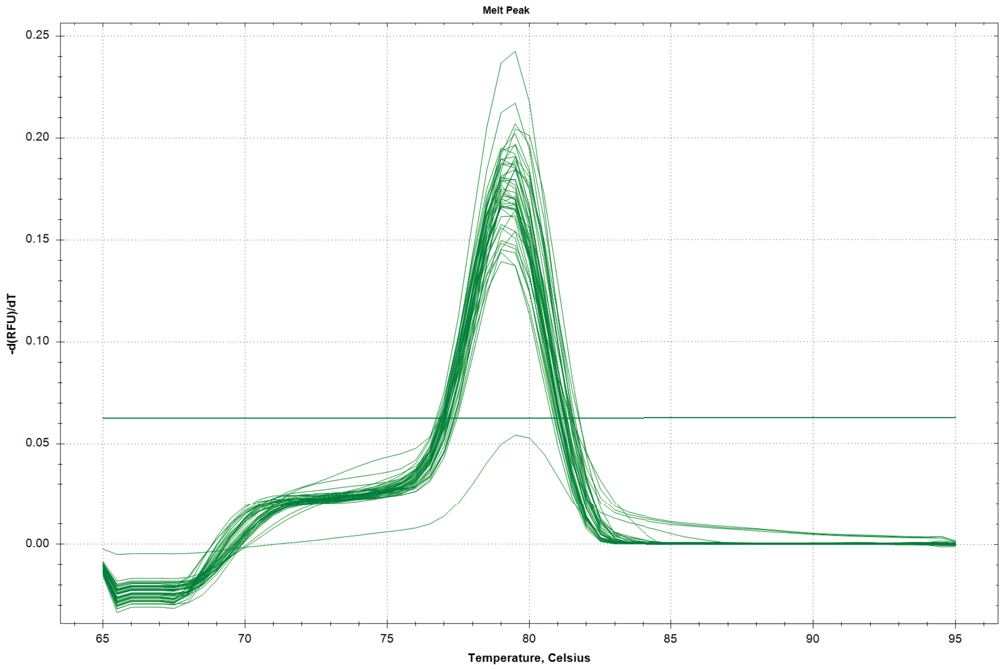

Melt Curve Data

Well	Fluor	Target	Content	Sample	Melt Temp
A01	SYBR		Std	lnc3rtF1R1	79.50
A02	SYBR		Std	lnc3rtF1R1	79.50
A03	SYBR		Std	lnc3rtF1R1	79.50
A04	SYBR		Std	lnc3rtF1R1	79.50
A05	SYBR		Std	lnc3rtF1R1	79.50
A06	SYBR		Std	lnc3rtF1R1	79.50
B01	SYBR		Unkn	Male Head 1st	79.50
B02	SYBR		Unkn	Male Abdomen	79.00
B03	SYBR		Unkn	Female Head	79.00
B04	SYBR		Unkn	Female Abdomen	79.00
B05	SYBR		Unkn	0h/1h cleavage	79.50
B06	SYBR		Unkn	0h/4h cleavage	79.50
C01	SYBR		Unkn	0h/8h cleavage	79.50
C02	SYBR		Unkn	0h/12h morula	79.00
C03	SYBR		Unkn	4h/12h morula	79.00
C04	SYBR		Unkn	8h/16h morula	79.00
C05	SYBR		Unkn	16h/24h morula	79.00
D01	SYBR		Unkn	Male Head 2nd	79.00

Melt Curve Data

Well	Fluor	Target	Content	Sample	Melt Temp
D02	SYBR		Unkn	Male Abdomen	79.00
D03	SYBR		Unkn	Female Head	79.00
D04	SYBR		Unkn	Female Abdomen	79.50
D05	SYBR		Unkn	0h/1h cleavage	79.50
D06	SYBR		Unkn	0h/4h cleavage	79.50
E01	SYBR		Unkn	0h/8h cleavage	79.00
E02	SYBR		Unkn	0h/12h morula	79.00
E03	SYBR		Unkn	4h/12h morula	79.00
E04	SYBR		Unkn	8h/16h morula	79.00
E05	SYBR		Unkn	16h/24h morula	79.00
F01	SYBR		Unkn	Male Head 3rd	79.00
F02	SYBR		Unkn	Male Abdomen	79.00
F03	SYBR		Unkn	Female Head	79.00
F04	SYBR		Unkn	Female Abdomen	79.00
F05	SYBR		Unkn	0h/1h cleavage	79.50
F06	SYBR		Unkn	0h/4h cleavage	79.50
G01	SYBR		Unkn	0h/8h cleavage	79.50
G02	SYBR		Unkn	0h/12h morula	79.00
G03	SYBR		Unkn	4h/12h morula	79.00
G04	SYBR		Unkn	8h/16h morula	79.00
G05	SYBR		Unkn	16h/24h morula	79.00
H01	SYBR		Std	lnc3rtF1R1	79.50
H02	SYBR		Std	lnc3rtF1R1	79.50
H03	SYBR		Std	lnc3rtF1R1	79.50
H04	SYBR		Std	lnc3rtF1R1	79.50
H05	SYBR		Std	lnc3rtF1R1	79.50
H06	SYBR		Std	lnc3rtF1R1	None

End Point

Fluorophore/Target: SYBR

End Cycles to Average: 5
Mode: Percentage of Range - 10

Lowest RFU Value: -0.000950
Highest RFU Value: 0.689
Cut Off Value:

SYBR End Point Data

Well	Fluor	Content	Sample	End RFU	Call
A01	SYBR	Std	lnc3rtF1R1	0.501	
A02	SYBR	Std	lnc3rtF1R1	-0.000335	
A03	SYBR	Std	lnc3rtF1R1	-0.000950	
A04	SYBR	Std	lnc3rtF1R1	0.591	
A05	SYBR	Std	lnc3rtF1R1	0.556	
A06	SYBR	Std	lnc3rtF1R1	0.611	
B01	SYBR	Unkn	Male Head 1st	0.419	
B02	SYBR	Unkn	Male Abdomen	0.355	
B03	SYBR	Unkn	Female Head	0.332	
B04	SYBR	Unkn	Female Abdomen	0.313	
B05	SYBR	Unkn	0h/1h cleavage	0.459	
B06	SYBR	Unkn	0h/4h cleavage	0.542	
C01	SYBR	Unkn	0h/8h cleavage	0.486	
C02	SYBR	Unkn	0h/12h morula	0.447	
C03	SYBR	Unkn	4h/12h morula	0.449	
C04	SYBR	Unkn	8h/16h morula	0.458	
C05	SYBR	Unkn	16h/24h morula	0.399	
D01	SYBR	Unkn	Male Head 2nd	0.369	
D02	SYBR	Unkn	Male Abdomen	0.349	
D03	SYBR	Unkn	Female Head	0.334	
D04	SYBR	Unkn	Female Abdomen	0.509	
D05	SYBR	Unkn	0h/1h cleavage	0.523	
D06	SYBR	Unkn	0h/4h cleavage	0.537	
E01	SYBR	Unkn	0h/8h cleavage	0.383	
E02	SYBR	Unkn	0h/12h morula	0.429	
E03	SYBR	Unkn	4h/12h morula	0.467	
E04	SYBR	Unkn	8h/16h morula	0.486	
E05	SYBR	Unkn	16h/24h morula	0.495	
F01	SYBR	Unkn	Male Head 3rd	0.464	

SYBR End Point Data

Well	Fluor	Content	Sample	End RFU	Call
F02	SYBR	Unkn	Male Abdomen	0.436	
F03	SYBR	Unkn	Female Head	0.322	
F04	SYBR	Unkn	Female Abdomen	0.509	
F05	SYBR	Unkn	0h/1h cleavage	0.511	
F06	SYBR	Unkn	0h/4h cleavage	0.517	
G01	SYBR	Unkn	0h/8h cleavage	0.540	
G02	SYBR	Unkn	0h/12h morula	0.534	
G03	SYBR	Unkn	4h/12h morula	0.523	
G04	SYBR	Unkn	8h/16h morula	0.483	
G05	SYBR	Unkn	16h/24h morula	0.541	
H01	SYBR	Std	lnc3rtF1R1	0.616	
H02	SYBR	Std	lnc3rtF1R1	0.573	
H03	SYBR	Std	lnc3rtF1R1	0.607	
H04	SYBR	Std	lnc3rtF1R1	0.621	
H05	SYBR	Std	lnc3rtF1R1	0.689	
H06	SYBR	Std	lnc3rtF1R1	0.148	

QC Parameters

Data

Description	Value	Use	Results	Exclude Wells	All excluded wells
Negative control with a Cq less than	38	True		False	
NTC with a Cq less than	38	True		False	
NRT with a Cq less than	38	True		False	
Positive control with a Cq greater than	30	True		False	
Unknown without a Cq	N/A	True		False	
Standard without a Cq	N/A	True	SYBR:A2, A3.	False	
Efficiency greater than	110.0	True			
Efficiency less than	90.0	True			

Data

Description	Value	Use	Results	Exclude Wells	All excluded wells
Std Curve R^2 less than	0.980	True			
Replicate group Cq Std Dev greater than	0.20	True		False	
